# Assessing sterility techniques in bronchodilator responsiveness testing by practicing allergists in North America

**DOI:** 10.1016/j.jacig.2024.100325

**Published:** 2024-08-22

**Authors:** Kabir Chhabra, Dhruva Gupta, Neel Singh, Naba Sharif, Sudhir Sekhsaria

**Affiliations:** a University of Maryland Hospital, Baltimore, Md; b Harvard Medical School, Boston, Mass; c MedStar Union Memorial Hospital, Baltimore, Md; d Penn Medicine-Becker ENT & Allergy, Princeton, NJ

**Keywords:** Bronchodilator reversibility testing, sterility, allergists, asthma

## Abstract

**Background:**

The American Thoracic Society has published general guidelines for sterility when testing for bronchodilator responsiveness. However, the extent to which practicing allergists implement sterility measures is currently unknown.

**Objective:**

This study aims to understand the adherence to the American Thoracic Society guidelines for sterility among practicing allergists.

**Methods:**

In 2015, a questionnaire was approved and distributed by the American Academy of Allergy, Asthma & Immunology to all its members. The anonymous responses were recorded and tabulated after a 3-week period.

**Results:**

Of the 6800 allergists who received surveys from the American Academy of Allergy, Asthma & Immunology members, 496 participated in the survey (response rate 7.3%). Using metered dose inhalers with a spacer and nebulizers were the most common bronchodilator administration techniques, as indicated by 59.35% and 58.52% of responses, respectively. Whereas 69.25% of the allergists considered their bronchodilator administration techniques to be sterile, 14.05% did not consider their administration technique to be sterile and 16.70% were unsure. For maintaining sterility, 38.75% of the respondents indicated using a new disposable attachment for reused inhalers, 18.71% indicated using a new inhaler for each patient, and 9.13% reported wiping inhalers with a cleaning agent.

**Conclusions:**

When asked about the sterility of the techniques used by them, nearly one-third of the allergists either stated that the measures used by them were unsterile or stated that they were were unsure. To increase adherence to sterility measures among North American allergists, promoting guideline awareness and proposing updated guidelines focused on the most common bronchodilator administration techniques is essential.

## Introduction

Implementing bronchodilator responsiveness testing is crucial in the diagnosis and management of asthma. Given the test’s utility, the American Thoracic Society (ATS) has previously published recommended guidelines to standardize the assessment of bronchodilator responsiveness for all clinicians.^1^ Prior studies have shown a deviation from established ATS guidelines for bronchodilator responsiveness testing based on type and dosage of bronchodilator used, wait time before measuring postbronchodilator spirometry, and threshold to designate as positive bronchodilator response.^2^^,^^3^ To our knowledge, no study thus far has evaluated deviation from the ATS guidelines specifically for the practice of sterility.

Per the 2005 version of the ATS guidelines, the technical statement discusses sterility under the section Cross-contamination.^1^ Providers must ensure that reusable spirometry components, including mouthpieces, breathing tubes, valves, and manifolds, are regularly disinfected and/or sterilized.^1^ Given the risk of certain sterilization procedures in damaging spirometry equipment, a provider should follow the recommendations by the manufacturer or an institution’s infection control department.^1^ We found that the 2005 version of ATS guidelines did not substantially differ from the 2019 version in terms of sterility practices.^1^^,^^4^

Failure to practice sterility can have detrimental consequences. The repeated use of metered dose inhalers (MDIs) without implementing sterility practices can increase the risk of infection.^5^^,^^6^ However, following proper procedures can mitigate infectious spread and reduce hospitalizations.^7^, ^8^, ^9^, ^10^, ^11^, ^12^, ^13^, ^14^, ^15^, ^16^ Although there are reports of bacteria remaining despite wiping a inhaler’s canister with an alcohol prep pad, sterilization protocols can reduce the risk even if it cannot be completely eliminated.^9^^,^^17^^,^^18^ Similar studies have also been conducted to assess the risk of infection for nebulizers.^19^, ^20^, ^21^

During the coronavirus disease 2019 (COVID-19) pandemic, a drastic shortage required hospitals to reuse MDIs for patients with asthma and other respiratory illnesses.^22^ Furthermore, nebulizers were thought to increase the risk of transmission by aerosolizing viral particles from patients with COVID-19.^21^ Guidelines were therefore proposed to sterilize MDIs and nebulizers to mitigate the spread while also ensuring that patients could access and administer their medications.^18^^,^^21^^,^^22^ Although the practice of sterility may have changed in response to the pandemic, our goal was to understand prepandemic adherence to sterility among allergists in North America. To this end, our study provides a baseline to better understand how sterility practices have changed over time.

A questionnaire regarding bronchodilator testing was approved by the American Academy of Allergy, Asthma, & Immunology (AAAAI) and distributed in 2015. We chose to analyze 3 questions related to sterility practices as follows: (1) what method do you use for bronchodilator administration in the office? (2) do you think that your asthma medication administration is sterile? and (3) how do you maintain inhaler sterility between patients? Because the study used data from an anonymous survey that did not ask for personal health information, it was considered compliant with the Health Insurance Portability and Accountability Act (HIPAA) and did not require institutional review board approval.

## Results and discussion

Of the 6800 allergists belonging to the AAAAI to whom surveys were sent in 2015, a total of 496 partially or fully completed the survey, yielding a response rate of 7.3%. The survey results demonstrated that 96.31% of allergists administer bronchodilator testing in their offices. The 3.69% of allergists surveyed (n = 18) who responded that they do not use bronchodilator testing as a part of their screening explained their decision to not use bronchodilator testing by stating that such testing is performed separately in either a hospital or a specialized pulmonary laboratory.

**Fig 1:**
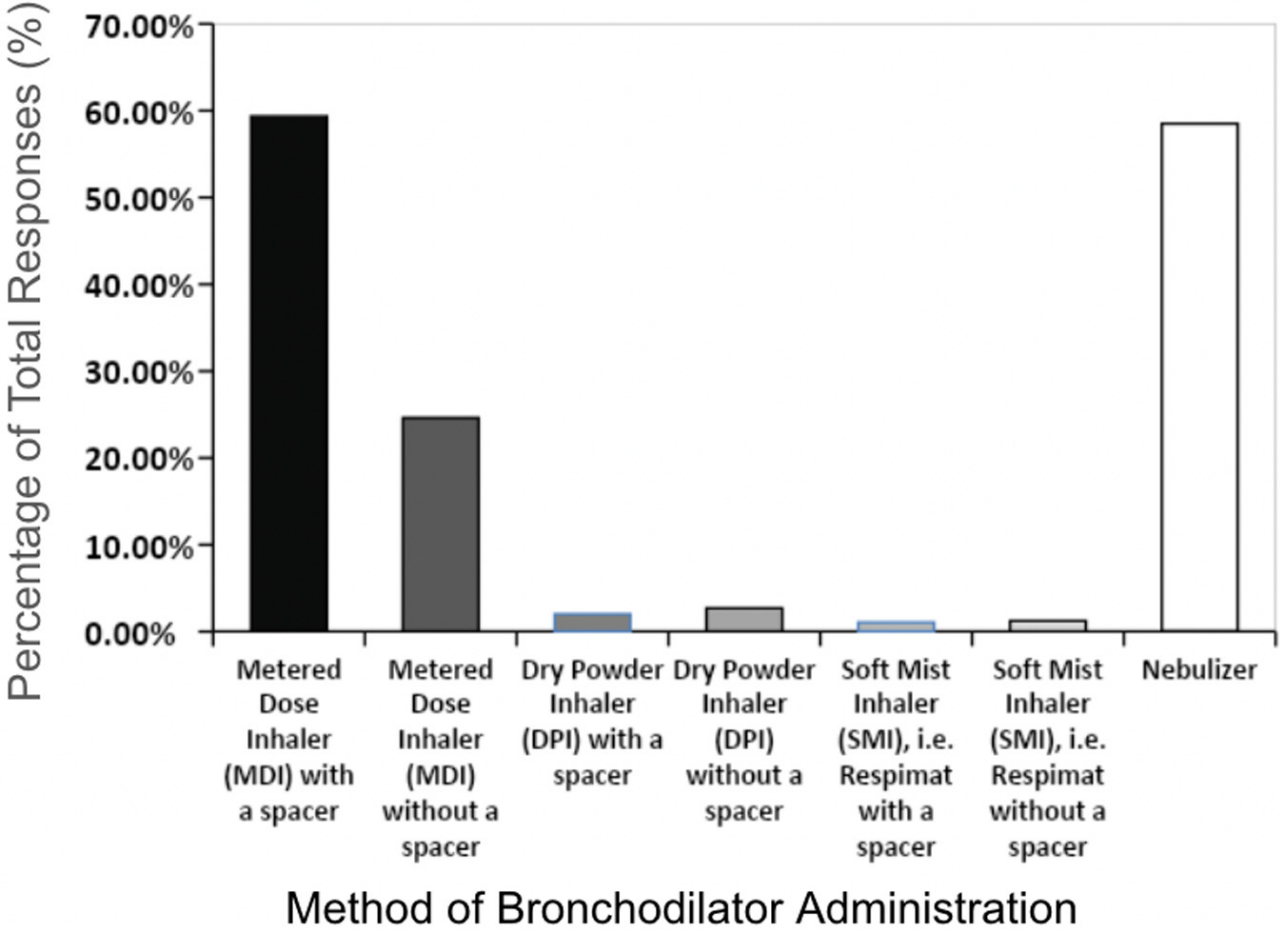
Methods that allergists use for bronchodilator administration. The most commonly used methods were MDIs with spacers (59.34% [n = 289]) use and nebulizers (58.52% [n = 285]).

In all, 487 allergists (98.1%) responded the first selected question, which addressed the method of bronchodilator administration. Using an MDI with a spacer and nebulizers were the most common bronchodilator administration techniques mentioned, accounting for 59.35% (n = 289) and 58.52% (n = 285) of the responses, respectively (Fig 1). These techniques were followed by using an MDI without a spacer, a dry powder inhaler with and without a spacer, and a soft mist inhaler with and without a spacer, accounting for 24.64% (n = 120), 4.72% (n = 23), and 2.26% (n = 11) of the responses, respectively. On the basis of these data, using MDIs and nebulizers are the most common methods for administering medication for bronchodilator responsiveness testing, which is consistent with the findings in prior literature.^21^^,^^23^

**Fig 2:**
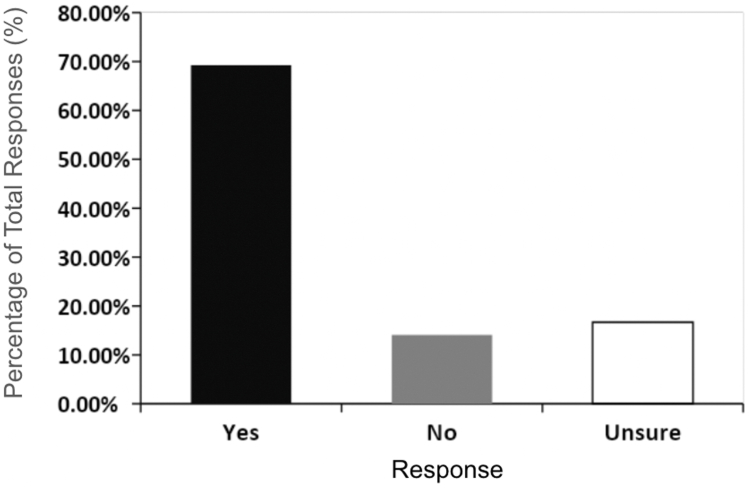
Physicians' opinion regarding whether their asthma medication administration is sterile. The majority of respondents (69.25% [n = 340]) believed that their techniques were sterile, whereas a significant portion either believed that their administration was not sterile (14.05% [n = 69]) or were unsure (16.70% [n = 82]).

A total of 491 allergists (99.0%) responded to the second selected question, which addressed whether administration of bronchodilator testing is sterile. The majority of the allergists (69.25% [n = 340]) considered their bronchodilator administration techniques to be sterile (Fig 2). On the other hand, 14.05% of the allergists (n = 69) did not consider their administration technique to be sterile and 16.70% (n = 82) were unsure. The reasons why 30.75% of the respondents (n = 156) were unsure or did not practice sterility when administering bronchodilator medications include lack of awareness for guidelines and unclear or conflicting guidelines. Several studies have shown how lack of awareness by providers poses barriers for adherence to infection control guidelines.^24^ Even if practicing allergists are aware of the ATS guidelines, there might be confusion, given the inconsistencies in previously published guidelines, which can obscure the steps necessary to practice sterility.^25^ Our survey did not ask respondents whether they were aware of guidelines by the ATS or other societies.

**Fig 3:**
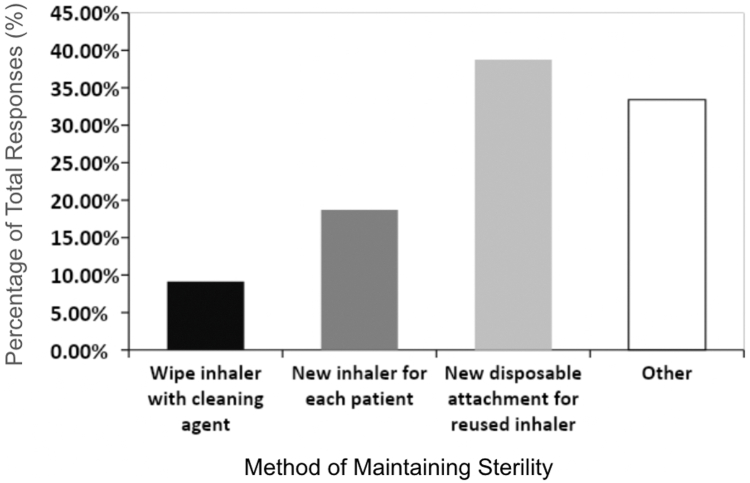
How physicians maintain inhaler sterility between each patient. Respondents reported that they use a new disposable attachment for a reused inhaler (38.75% [n = 174]), wipe the inhaler with a cleansing agent after use (9.13% [n = 41]), and/or use a new inhaler for each patient (18.71% [n = 84]). Some respondents (33.41% [n = 150]) replied Other.

The third selected question, which addressed maintaining inhaler sterility, elicited responses from 449 of the allergists (90.5%). Methods of maintaining inhaler sterility, such as using a new disposable attachment for reused inhalers, using a new inhaler for each patient, wiping inhalers with a cleaning agent, and using another method accounted for 38.75% (n = 174), 18.71% (n = 84), 9.13% (n = 41), and 33.41% (n = 150) of the responses, respectively (Fig 3). Among those respondents who used another method, common explanations included sterilizing the spacer of an inhaler after every use; sterilizing the tubing, mask, and mouthpiece of the nebulizer; using a new nebulizer or component of nebulizer for each patient; and rinsing equipment with soap and water. It should also be noted that many explanations emphasized that the allergist used only a nebulizer for this purpose rather than inhalers.

Using a new inhaler as well as a disposable attachment for a reused inhaler adheres to the ATS guidelines, whereas wiping the inhaler with a cleaning agent possibly adheres to guidelines if the manufacturer’s recommendations are followed. Among the explanations from allergists who selected the response Other, a majority showed adherence to ATS guidelines for both inhalers and nebulizers. A few respondents indicated using soap and water for sterility, which can possibly violate ATS guidelines unless the manufacturer has specified that soap and water can be used for rinsing equipment.

This study was limited in several respects. The survey was sent out in 2015 and therefore does not capture the current sterility practices of allergists in testing bronchodilator responsiveness after the COVID-19 pandemic. Because this survey collected prepandemic responses, the next step will be to determine the influence of COVID-19 and updated ATS guidelines on the current sterility practice of bronchodilator responsiveness testing.^4^ This direction would provide insight into the trend of adherence to sterility guidelines over time. Furthermore, although our survey did not inquire into the allergists’ awareness of the ATS guidelines, a future study could address this gap and also elucidate the reasons why proper sterility practices are not being implemented. Another limitation is the fact that the relevant ATS guidelines at the time of survey distribution were from 2005, whereas a new set of guidelines was released in 2019.^1^^,^^4^ However, the 2019 guidelines did not include any significant changes to the recommended sterility protocols. Despite the different numbers of responses for each of the selected survey questions, we were unable to ascertain how many allergists answered all 3 questions based on the survey data. Furthermore, the data did not include information on how many allergists selected more than 1 response when answering each of the selected questions. Ideally, a higher response rate for our survey would have made the data more valid and generalizable from the standpoint of representation of practicing allergists in North America. The final limitation is that the third selected survey question directly included answer choices about sterility practices for inhalers but did not include any answer choices about sterility practices for nebulizers. If allergists sterilized their nebulizers, they had to write out their response after selecting Other. This may explain why the third selected question had a 90.5% response rate whereas the first 2 questions had rates of 98.1% and 99.0%, respectively.

Overall, we have demonstrated that nearly one-third of responding allergists reported that they did not practice or were unsure that they practiced sterility in bronchodilator responsiveness according to the ATS guidelines. Although we did not assess whether survey respondents were aware of the ATS guidelines, visibility should nonetheless be promoted so that practicing allergists can be aware of proper sterility practices. Furthermore, revised guidelines should focus on providing specific recommendations to preserve sterility for MDI with spacers and nebulizers because these were the most common methods of bronchodilator administration. Proposing new guidelines can optimize adherence to sterility practices, which can mitigate the risk of infection, promote cost savings for hospitals and clinics, and ensure patient safety.

For details on the study methodology and findings, see the Online Repository at www.jaci-global.org.

**Key messages**

• Lack of sterility when conducting bronchodilator responsiveness testing can increase the risk of infectious transmission and compromise patient safety.
• Understanding the adoption of sterility techniques in bronchodilator responsiveness before the pandemic not only establishes a baseline for how sterility is practiced over time but also provides insights into optimizing adherence among allergists.

## Disclosure statement

Disclosure of potential conflict of interest: The authors declare that they have no relevant conflicts of interest.
