# Supplementary material for: Assessing sterility techniques in bronchodilator responsiveness testing by practicing allergists in North America
Source: J Allergy Clin Immunol Glob. 2024 Aug 22;3(4):100325. doi: 10.1016/j.jacig.2024.100325 (PMC11426031; doi:10.1016/j.jacig.2024.100325)
Supplement: Supplementary data [file mmc1.docx]

A questionnaire regarding bronchodilator testing was created and sent to the American Academy of Allergy, Asthma, and Immunology (AAAAI) for approval and distribution in 2015. The anonymously recorded responses were separated, tabulated, and returned after a 3-week period. There were a total of 15 questions on the survey, of which three were selected based on the relevance to sterility practices, as follows: 1. What method do you use for bronchodilator administration in the office? 2. Do you think your asthma medication administration is sterile? 3. How do you maintain inhaler sterility between each patient? Because the study used data from an anonymous survey that did not ask for personal health information(PHI), it was considered compliant with the Health Insurance Portability and Accountability Act (HIPAA) and did not require Institutional Review Board (IRB).
