# Supplementary material for: Assessing sterility techniques in bronchodilator responsiveness testing by practicing allergists in North America
Source: J Allergy Clin Immunol Glob. 2024 Aug 22;3(4):100325. doi: 10.1016/j.jacig.2024.100325 (PMC11426031; doi:10.1016/j.jacig.2024.100325)
Supplement: Supplementary Materials [file mmc2.pdf]

Q1 Do you perform bronchodilator reversibility testing in your office?

Answered: 488 Skipped: 8

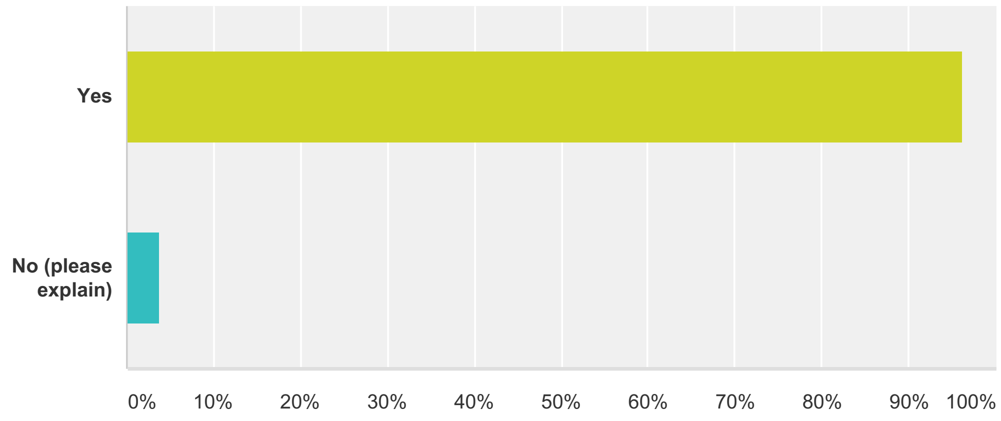

Answer Choices	Responses	
Yes	**96.31%**	470
No (please explain)	**3.69%**	18
**Total**		**488**

#	No (please explain)	Date
1	At a University and all testing is performed in specialized center for lung function studies.	6/16/2015 3:37 PM
2	My practice is limited to immunodeficiency.	6/16/2015 10:09 AM
3	I do basic research	6/16/2015 9:59 AM
4	Our office is hospital-based and we use the adjacent PFT laboratory	6/16/2015 9:14 AM
5	Refer to pulmonary consultant	6/10/2015 9:57 AM
6	n/a	6/10/2015 9:03 AM
7	Done in hospital PFT lab	6/9/2015 8:03 PM
8	hospital practice done in pulmonary	6/9/2015 2:29 PM
9	But I am now retired	6/9/2015 1:58 PM
10	Part of a multi-specialty group practice; bronchodilator reversibility testing is done in the pulmonary department.	6/9/2015 7:24 AM
11	na	6/4/2015 11:59 AM
12	We are a hospital based clinic, so the testing is performed in a separate pulmonary function lab. We only provide office spirometry in the clinic. In the rare case we check spiro pre/post, a neb is used with fresh tubing, as below.	6/4/2015 11:40 AM
13	I found its not necessary to effectively manage an Allergy or an Asthmatic patient.	6/4/2015 5:57 AM
14	I work at a military MTF and have no functioning spirometer despite asking for one over and over since August 2014. The information I am entering is how pulmonary does it which I have to go through pulmonary to get my PFTs.	6/3/2015 7:45 PM
15	rarely I check pre and post spirometry	6/2/2015 8:03 PM
16	It is done in our pulmonary function laboratory	6/2/2015 7:43 PM

17	pre-and post administration of albuterol or Levalbuterol via MDI or nebulizer I specific circumstances.	6/2/2015 5:49 PM
18	Very rarely	6/2/2015 4:16 PM

Q2 What method do you use for bronchodilator administration in the office? (Check All That Apply)

Answered: 487 Skipped: 9

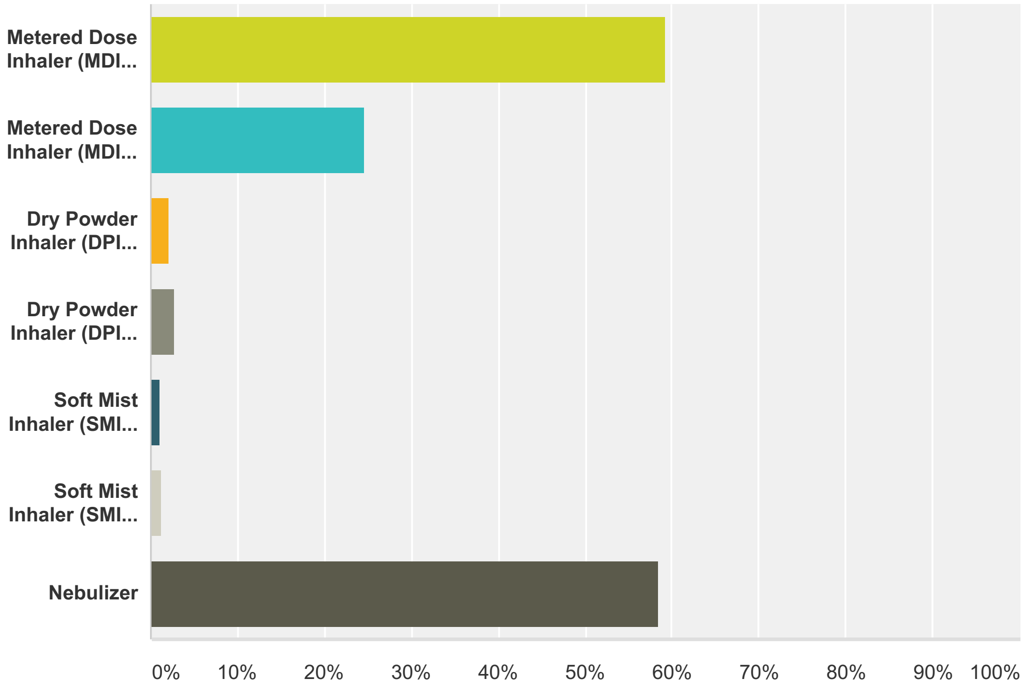

Answer Choices	Responses	
Metered Dose Inhaler (MDI) with a spacer	**59.34%**	289
Metered Dose Inhaler (MDI) without a spacer	**24.64%**	120
Dry Powder Inhaler (DPI) with a spacer	**2.05%**	10
Dry Powder Inhaler (DPI) without a spacer	**2.67%**	13
Soft Mist Inhaler (SMI), i.e. Respimat with a spacer	**1.03%**	5
Soft Mist Inhaler (SMI), i.e. Respimat without a spacer	**1.23%**	6
Nebulizer	**58.52%**	285
**Total Respondents: 487**		

Q3 If using an MDI, how many puffs:

Answered: 349 Skipped: 147

#	Responses	Date
1	2	6/19/2015 4:48 PM
2	2	6/19/2015 12:50 PM
3	2	6/19/2015 12:32 PM
4	2-4	6/18/2015 5:24 PM
5	4	6/18/2015 4:49 PM
6	2 - 4	6/18/2015 9:56 AM
7	2	6/17/2015 4:53 PM
8	two	6/17/2015 4:01 PM
9	2	6/17/2015 10:54 AM
10	2	6/17/2015 9:43 AM
11	2 - 4 puffs	6/16/2015 9:38 PM
12	4	6/16/2015 9:36 PM
13	2	6/16/2015 9:00 PM
14	2	6/16/2015 8:33 PM
15	2	6/16/2015 7:01 PM
16	2	6/16/2015 6:00 PM
17	3 - 4	6/16/2015 4:09 PM
18	2 to 4	6/16/2015 2:42 PM
19	3	6/16/2015 2:24 PM
20	2	6/16/2015 2:10 PM
21	2	6/16/2015 1:36 PM
22	4	6/16/2015 12:55 PM
23	2	6/16/2015 12:33 PM
24	Two	6/16/2015 12:22 PM
25	two	6/16/2015 12:12 PM
26	2	6/16/2015 11:55 AM
27	2	6/16/2015 11:33 AM
28	2 puffs	6/16/2015 11:33 AM
29	4	6/16/2015 11:00 AM
30	3	6/16/2015 10:58 AM
31	3 to 4 puffs	6/16/2015 10:38 AM
32	2	6/16/2015 10:12 AM
33	2	6/16/2015 10:08 AM

34	2	6/16/2015 10:05 AM
35	2	6/16/2015 10:05 AM
36	2	6/16/2015 9:56 AM
37	2	6/16/2015 9:45 AM
38	2	6/16/2015 9:29 AM
39	2	6/16/2015 9:23 AM
40	2 to 4	6/16/2015 9:14 AM
41	2-4	6/16/2015 9:10 AM
42	2	6/16/2015 9:10 AM
43	4	6/16/2015 9:09 AM
44	2-3	6/16/2015 9:06 AM
45	2	6/16/2015 9:05 AM
46	2	6/15/2015 8:48 PM
47	2 puffs	6/15/2015 8:44 PM
48	2	6/14/2015 9:41 PM
49	4puffs	6/14/2015 8:35 PM
50	2 puffs 5 min apart	6/14/2015 7:25 PM
51	4	6/14/2015 4:52 PM
52	2 puff	6/13/2015 8:39 PM
53	2	6/13/2015 9:11 AM
54	2	6/12/2015 9:10 PM
55	2	6/12/2015 12:27 PM
56	2	6/12/2015 6:19 AM
57	4	6/11/2015 9:23 AM
58	2	6/11/2015 12:00 AM
59	4	6/10/2015 5:29 PM
60	2	6/10/2015 3:17 PM
61	2-4	6/10/2015 2:43 PM
62	2	6/10/2015 2:14 PM
63	4	6/10/2015 2:00 PM
64	6-8	6/10/2015 1:19 PM
65	3	6/10/2015 12:50 PM
66	2-4	6/10/2015 11:58 AM
67	2	6/10/2015 11:32 AM
68	2	6/10/2015 11:26 AM
69	4	6/10/2015 11:25 AM
70	2	6/10/2015 10:26 AM
71	2	6/10/2015 9:14 AM

72	2	6/10/2015 9:06 AM
73	n/a	6/10/2015 9:03 AM
74	2 to 6, depending upon patient age	6/10/2015 8:58 AM
75	2	6/10/2015 8:08 AM
76	2	6/10/2015 7:53 AM
77	2	6/10/2015 6:04 AM
78	4	6/10/2015 1:54 AM
79	four	6/10/2015 12:39 AM
80	4 puff	6/9/2015 10:28 PM
81	2	6/9/2015 9:56 PM
82	2	6/9/2015 8:46 PM
83	Depends on weight but usually 4 puffs of ventolin, 2 puffs of bricanyl	6/9/2015 8:03 PM
84	2	6/9/2015 7:48 PM
85	2 puffs	6/9/2015 7:48 PM
86	2	6/9/2015 7:17 PM
87	4	6/9/2015 7:10 PM
88	2	6/9/2015 6:57 PM
89	2	6/9/2015 6:55 PM
90	2	6/9/2015 6:27 PM
91	2 puffs	6/9/2015 6:13 PM
92	2	6/9/2015 6:00 PM
93	2	6/9/2015 5:23 PM
94	4	6/9/2015 5:21 PM
95	2	6/9/2015 5:08 PM
96	2	6/9/2015 5:03 PM
97	4	6/9/2015 5:00 PM
98	2 puffs	6/9/2015 4:53 PM
99	2	6/9/2015 4:06 PM
100	4 puffs	6/9/2015 4:03 PM
101	4	6/9/2015 3:44 PM
102	4	6/9/2015 3:41 PM
103	4 puffs	6/9/2015 3:38 PM
104	2	6/9/2015 3:32 PM
105	4	6/9/2015 3:27 PM
106	2	6/9/2015 3:25 PM
107	2-3	6/9/2015 3:22 PM
108	6	6/9/2015 3:08 PM
109	2 or 4	6/9/2015 3:01 PM

110	2	6/9/2015 2:55 PM
111	2	6/9/2015 2:49 PM
112	4	6/9/2015 2:39 PM
113	4	6/9/2015 2:31 PM
114	4	6/9/2015 2:29 PM
115	2	6/9/2015 2:29 PM
116	2	6/9/2015 2:23 PM
117	4	6/9/2015 1:58 PM
118	4	6/9/2015 1:58 PM
119	2	6/9/2015 1:44 PM
120	2-4	6/9/2015 1:40 PM
121	4	6/9/2015 1:29 PM
122	4 in adults, 2 in kids	6/9/2015 1:27 PM
123	4	6/9/2015 1:19 PM
124	2puffs	6/9/2015 1:13 PM
125	4	6/9/2015 1:12 PM
126	4 puffs	6/9/2015 1:11 PM
127	2	6/9/2015 1:11 PM
128	4	6/9/2015 1:07 PM
129	4	6/9/2015 7:41 AM
130	2	6/9/2015 7:24 AM
131	2	6/9/2015 4:56 AM
132	3	6/9/2015 1:59 AM
133	2-4 puffs	6/8/2015 8:34 PM
134	2 puffs	6/8/2015 1:36 PM
135	2	6/8/2015 12:10 PM
136	2-4	6/8/2015 11:34 AM
137	2	6/8/2015 7:42 AM
138	2	6/7/2015 9:04 PM
139	2	6/7/2015 4:35 PM
140	2-4	6/7/2015 3:19 PM
141	2	6/7/2015 2:48 PM
142	Two	6/7/2015 2:37 PM
143	2-4 puffs depending.	6/7/2015 1:33 PM
144	4	6/7/2015 12:01 PM
145	4 for most adults, 2 for kids	6/6/2015 9:35 PM
146	2	6/6/2015 12:16 AM
147	2	6/5/2015 8:41 PM

148	one, wait a while, one more	6/5/2015 6:30 PM
149	2	6/5/2015 3:32 PM
150	2	6/5/2015 2:51 PM
151	2	6/5/2015 2:26 PM
152	2	6/5/2015 12:29 PM
153	2	6/5/2015 12:03 PM
154	2	6/5/2015 10:34 AM
155	1	6/4/2015 5:46 PM
156	2-3	6/4/2015 4:30 PM
157	2	6/4/2015 4:28 PM
158	4	6/4/2015 3:21 PM
159	4	6/4/2015 2:55 PM
160	4	6/4/2015 12:40 PM
161	na	6/4/2015 11:59 AM
162	2	6/4/2015 10:38 AM
163	2 to 4	6/4/2015 8:42 AM
164	2	6/4/2015 7:34 AM
165	2	6/4/2015 7:33 AM
166	2	6/4/2015 7:17 AM
167	4	6/4/2015 5:50 AM
168	4	6/3/2015 10:11 PM
169	Two	6/3/2015 9:43 PM
170	albuterol (any brand) 2 puffs	6/3/2015 8:23 PM
171	2-4	6/3/2015 8:17 PM
172	2 to 4	6/3/2015 7:56 PM
173	4 puffs	6/3/2015 7:45 PM
174	2	6/3/2015 7:35 PM
175	2	6/3/2015 6:48 PM
176	3	6/3/2015 5:09 PM
177	2 If patient has his/her own	6/3/2015 4:55 PM
178	2	6/3/2015 4:54 PM
179	2 puffs	6/3/2015 4:42 PM
180	2 puffs	6/3/2015 4:37 PM
181	4	6/3/2015 3:37 PM
182	2	6/3/2015 3:33 PM
183	2 to 4	6/3/2015 3:06 PM
184	4	6/3/2015 2:08 PM
185	4	6/3/2015 1:56 PM

186	2-3	6/3/2015 1:10 PM
187	n/a	6/3/2015 1:06 PM
188	Two	6/3/2015 12:56 PM
189	3	6/3/2015 12:49 PM
190	2-4	6/3/2015 12:07 PM
191	2 to 4	6/3/2015 11:46 AM
192	2-3	6/3/2015 11:27 AM
193	2 or 4	6/3/2015 11:17 AM
194	4	6/3/2015 11:15 AM
195	2	6/3/2015 11:06 AM
196	2	6/3/2015 11:03 AM
197	4	6/3/2015 10:50 AM
198	4	6/3/2015 10:34 AM
199	2 puff	6/3/2015 10:21 AM
200	2	6/3/2015 10:17 AM
201	2-3	6/3/2015 10:10 AM
202	2-4	6/3/2015 10:08 AM
203	2	6/3/2015 9:59 AM
204	2 - 4 (depending on age)	6/3/2015 9:48 AM
205	2-4	6/3/2015 9:31 AM
206	Two.	6/3/2015 9:21 AM
207	2-4	6/3/2015 9:11 AM
208	4	6/3/2015 8:58 AM
209	4	6/3/2015 8:43 AM
210	2	6/3/2015 8:40 AM
211	1 -2 puffs foradil	6/3/2015 8:38 AM
212	2	6/3/2015 8:19 AM
213	2 puffs	6/3/2015 8:13 AM
214	4	6/3/2015 8:11 AM
215	2	6/3/2015 8:10 AM
216	2 to 4	6/3/2015 8:01 AM
217	4	6/3/2015 7:53 AM
218	2	6/3/2015 7:50 AM
219	2	6/3/2015 7:45 AM
220	2-4	6/3/2015 7:33 AM
221	2-4	6/3/2015 6:39 AM
222	2 to 4	6/3/2015 6:31 AM
223	2	6/3/2015 6:05 AM

224	3	6/3/2015 5:49 AM
225	2	6/3/2015 4:09 AM
226	2	6/3/2015 3:47 AM
227	2-4	6/2/2015 11:11 PM
228	4	6/2/2015 10:51 PM
229	2	6/2/2015 10:43 PM
230	2	6/2/2015 10:37 PM
231	2	6/2/2015 9:52 PM
232	4	6/2/2015 9:49 PM
233	2	6/2/2015 9:39 PM
234	2	6/2/2015 9:39 PM
235	4	6/2/2015 9:20 PM
236	2 followed 2more if unresponsive	6/2/2015 9:16 PM
237	2	6/2/2015 9:12 PM
238	2	6/2/2015 9:01 PM
239	2	6/2/2015 8:51 PM
240	4	6/2/2015 8:45 PM
241	2	6/2/2015 8:43 PM
242	2	6/2/2015 8:36 PM
243	2	6/2/2015 8:19 PM
244	2	6/2/2015 8:19 PM
245	2	6/2/2015 8:06 PM
246	2	6/2/2015 8:03 PM
247	2	6/2/2015 7:57 PM
248	2	6/2/2015 7:56 PM
249	2	6/2/2015 7:47 PM
250	2	6/2/2015 7:31 PM
251	4	6/2/2015 7:13 PM
252	2 puffs	6/2/2015 7:10 PM
253	2 puffs	6/2/2015 7:07 PM
254	2	6/2/2015 7:07 PM
255	2 puffs using a disposable mouthpiece held over the inhaler mouthpiece.	6/2/2015 7:04 PM
256	4 puffs	6/2/2015 7:03 PM
257	2	6/2/2015 6:57 PM
258	4 puffs	6/2/2015 6:56 PM
259	2 and sometimes 4	6/2/2015 6:42 PM
260	two	6/2/2015 6:42 PM
261	3	6/2/2015 6:30 PM

262	2 puffs Xopenx HFA	6/2/2015 6:22 PM
263	2	6/2/2015 6:18 PM
264	4	6/2/2015 6:14 PM
265	2	6/2/2015 6:13 PM
266	4	6/2/2015 6:11 PM
267	2	6/2/2015 6:10 PM
268	2.	6/2/2015 6:03 PM
269	2 to 4	6/2/2015 5:54 PM
270	2	6/2/2015 5:51 PM
271	2	6/2/2015 5:49 PM
272	2-4	6/2/2015 5:47 PM
273	2	6/2/2015 5:47 PM
274	2	6/2/2015 5:38 PM
275	2	6/2/2015 5:36 PM
276	2 puffs	6/2/2015 5:35 PM
277	4	6/2/2015 5:32 PM
278	2-3	6/2/2015 5:31 PM
279	2	6/2/2015 5:30 PM
280	2	6/2/2015 5:29 PM
281	4	6/2/2015 5:22 PM
282	2	6/2/2015 5:19 PM
283	4 puffs	6/2/2015 5:09 PM
284	2	6/2/2015 5:08 PM
285	2	6/2/2015 5:06 PM
286	2	6/2/2015 5:05 PM
287	two	6/2/2015 5:00 PM
288	4 puffs	6/2/2015 4:54 PM
289	2	6/2/2015 4:54 PM
290	4	6/2/2015 4:49 PM
291	2	6/2/2015 4:46 PM
292	2 puffs HFA - rarely use this - typically use a neb	6/2/2015 4:43 PM
293	4	6/2/2015 4:42 PM
294	4	6/2/2015 4:37 PM
295	2	6/2/2015 4:32 PM
296	na	6/2/2015 4:24 PM
297	2	6/2/2015 4:23 PM
298	2	6/2/2015 4:16 PM
299	2	6/2/2015 4:10 PM

300	4	6/2/2015 4:09 PM
301	2	6/2/2015 4:08 PM
302	2 puffs	6/2/2015 4:08 PM
303	2	6/2/2015 4:08 PM
304	4	6/2/2015 4:05 PM
305	2-4	6/2/2015 4:05 PM
306	2	6/2/2015 4:02 PM
307	4	6/2/2015 4:01 PM
308	4	6/2/2015 4:01 PM
309	4	6/2/2015 3:59 PM
310	3 puffs	6/2/2015 3:58 PM
311	2	6/2/2015 3:56 PM
312	4	6/2/2015 3:56 PM
313	2	6/2/2015 3:56 PM
314	2	6/2/2015 3:54 PM
315	2	6/2/2015 3:50 PM
316	N/A	6/2/2015 3:48 PM
317	four	6/2/2015 3:45 PM
318	2	6/2/2015 3:42 PM
319	4	6/2/2015 3:41 PM
320	2	6/2/2015 3:40 PM
321	2	6/2/2015 3:38 PM
322	2	6/2/2015 3:37 PM
323	4 in adults, 2 in children	6/2/2015 3:35 PM
324	2	6/2/2015 3:35 PM
325	2	6/2/2015 3:34 PM
326	2-3	6/2/2015 3:32 PM
327	2	6/2/2015 3:31 PM
328	2	6/2/2015 3:31 PM
329	3-4	6/2/2015 3:30 PM
330	2	6/2/2015 3:30 PM
331	2	6/2/2015 3:30 PM
332	2	6/2/2015 3:29 PM
333	2	6/2/2015 3:29 PM
334	2	6/2/2015 3:28 PM
335	2	6/2/2015 3:28 PM
336	2	6/2/2015 3:27 PM
337	2-4	6/2/2015 3:27 PM

338	2	6/2/2015 3:26 PM
339	2	6/2/2015 3:25 PM
340	4	6/2/2015 3:25 PM
341	2	6/2/2015 3:24 PM
342	4	6/2/2015 3:23 PM
343	2	6/2/2015 3:23 PM
344	2	6/2/2015 3:23 PM
345	2	6/2/2015 3:23 PM
346	2 kids, 4 teens and adults	6/2/2015 3:23 PM
347	4	6/2/2015 3:23 PM
348	2	6/2/2015 3:22 PM
349	4	6/2/2015 3:21 PM

Q4 Which bronchodilators or bronchodilator combinations do you use for reversibility testing? (Check All That Apply)

Answered: 492 Skipped: 4

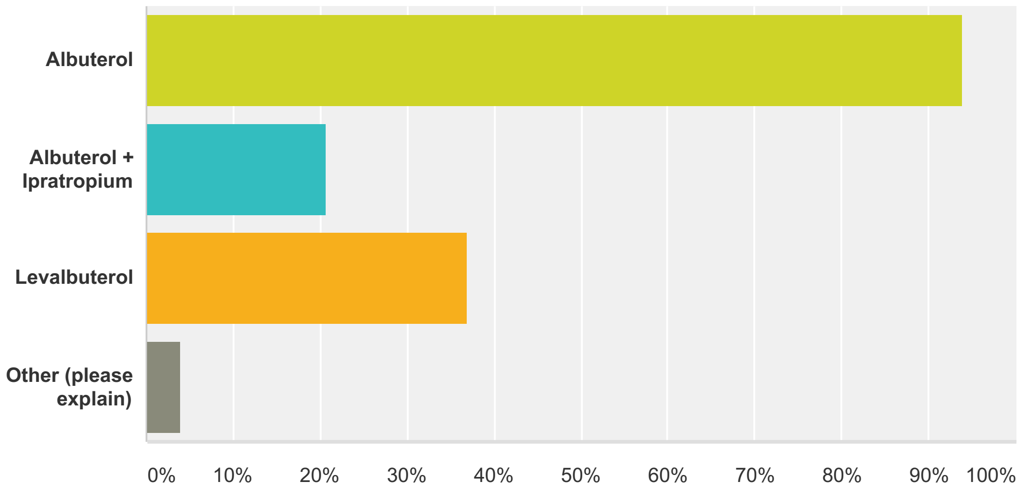

Answer Choices	Responses	
Albuterol	**93.90%**	462
Albuterol + Ipratropium	**20.73%**	102
Levalbuterol	**36.99%**	182
Other (please explain)	**4.07%**	20
**Total Respondents: 492**		

#	Other (please explain)	Date
1	ipratropium	6/16/2015 8:00 PM
2	If patient does not bring own device, we will use Zenhale (i.e. formoterol) because of samples on hand.	6/16/2015 4:09 PM
3	Albuterol or Symbicort	6/15/2015 8:44 PM
4	n/a	6/10/2015 9:03 AM
5	use levalbuterol if patient has a history of exaggerated side effects to albuterol	6/10/2015 12:39 AM
6	symbicort as only samples available and it works by 15 minutes	6/9/2015 8:46 PM
7	Salbutamol and terbutaline (in Canada)	6/9/2015 8:03 PM
8	Salbutamol - Canadian equivalent to Albuterol	6/9/2015 7:48 PM
9	Spiriva	6/9/2015 3:25 PM
10	symbicort	6/9/2015 2:29 PM
11	Symbicort or Dulera as well	6/5/2015 8:41 PM
12	na	6/4/2015 11:59 AM
13	I would prefer levalbuteral all the time, but it is quite expensive and we get samples of abuterol	6/3/2015 3:06 PM

14	Foradil	6/3/2015 8:38 AM
15	Symbicort or Dulera rarely	6/2/2015 8:51 PM
16	Dulera or Symbicort Form petrol also acts as a bronchodilator rapid onset of action	6/2/2015 8:43 PM
17	Salbutamol in canada	6/2/2015 6:42 PM
18	spiriva	6/2/2015 6:13 PM
19	Symbicort or Dulera	6/2/2015 3:32 PM
20	Ipratropium	6/2/2015 3:25 PM

Q5 Typically, how long after administering the bronchodilator do you wait before performing repeat spirometry?

Answered: 487 Skipped: 9

#	Responses	Date
1	20 minutes	6/19/2015 4:48 PM
2	5 minutes	6/19/2015 12:50 PM
3	20 min	6/19/2015 12:32 PM
4	10-15 minutes	6/18/2015 5:24 PM
5	15 min	6/18/2015 4:49 PM
6	15 min after finishing the neb	6/18/2015 12:03 PM
7	15-20 min	6/18/2015 9:56 AM
8	20 minutes	6/17/2015 4:53 PM
9	10 minutes	6/17/2015 4:01 PM
10	10 minutes	6/17/2015 2:37 PM
11	10 minutes	6/17/2015 10:54 AM
12	15-20min	6/17/2015 9:43 AM
13	5 - 15 minutes	6/16/2015 9:38 PM
14	15 min	6/16/2015 9:36 PM
15	10-15 minutes	6/16/2015 9:00 PM
16	15-20 mins	6/16/2015 8:33 PM
17	30 min per ATS guidelines	6/16/2015 8:00 PM
18	15	6/16/2015 7:01 PM
19	15 minutes	6/16/2015 6:00 PM
20	15 minutes	6/16/2015 5:43 PM
21	20 minutes	6/16/2015 4:09 PM
22	15 minutes	6/16/2015 3:37 PM
23	15 minutes	6/16/2015 2:42 PM
24	15 min.	6/16/2015 2:24 PM
25	15 minutes	6/16/2015 2:10 PM
26	10-15 min	6/16/2015 2:00 PM
27	20 minutes	6/16/2015 1:36 PM
28	15-20 minutes	6/16/2015 1:22 PM
29	20 min	6/16/2015 1:01 PM
30	10-15 minutes	6/16/2015 12:55 PM
31	20 minutes	6/16/2015 12:42 PM

32	15 minutes	6/16/2015 12:33 PM
33	5 min	6/16/2015 12:22 PM
34	10-20 min	6/16/2015 12:12 PM
35	20 mins	6/16/2015 11:55 AM
36	10 min	6/16/2015 11:36 AM
37	15	6/16/2015 11:33 AM
38	20 minutes	6/16/2015 11:33 AM
39	15 minutes	6/16/2015 11:18 AM
40	30 min	6/16/2015 11:00 AM
41	15 min	6/16/2015 10:58 AM
42	10 to 15 minutes	6/16/2015 10:38 AM
43	10-15 minutes	6/16/2015 10:12 AM
44	15 to 20 minutes	6/16/2015 10:08 AM
45	15	6/16/2015 10:05 AM
46	15-20 minutes	6/16/2015 10:05 AM
47	20min	6/16/2015 9:56 AM
48	15 minutes	6/16/2015 9:45 AM
49	15 minutes	6/16/2015 9:30 AM
50	10-15 minutes	6/16/2015 9:29 AM
51	15 mins	6/16/2015 9:23 AM
52	20 minutes	6/16/2015 9:15 AM
53	10 minutes	6/16/2015 9:14 AM
54	15-20 minutes	6/16/2015 9:12 AM
55	10 minutes	6/16/2015 9:12 AM
56	20 to 30 minutes.	6/16/2015 9:10 AM
57	10-15 min	6/16/2015 9:10 AM
58	5 minutes	6/16/2015 9:10 AM
59	20 minutes	6/16/2015 9:09 AM
60	20 min	6/16/2015 9:09 AM
61	15 minutes	6/16/2015 9:06 AM
62	15 min	6/16/2015 9:05 AM
63	15 minutes	6/15/2015 8:48 PM
64	15 minutes	6/15/2015 8:44 PM
65	20 minutes	6/14/2015 9:41 PM
66	15minutes	6/14/2015 8:35 PM
67	10 min. after 2nd puff	6/14/2015 7:25 PM
68	15 min	6/14/2015 4:52 PM
69	15 minutes	6/14/2015 1:43 AM

70	5 minute	6/13/2015 8:39 PM
71	15 min	6/12/2015 9:10 PM
72	10 min	6/12/2015 12:27 PM
73	15-20 minutes	6/12/2015 10:34 AM
74	20 minutes	6/12/2015 9:05 AM
75	15 minutes	6/12/2015 7:51 AM
76	8 min	6/12/2015 6:19 AM
77	10 mts	6/11/2015 11:34 AM
78	20 minutes	6/11/2015 9:23 AM
79	10 min	6/11/2015 12:00 AM
80	15 minutes	6/10/2015 5:29 PM
81	15 minutes	6/10/2015 3:17 PM
82	15 minutes	6/10/2015 2:53 PM
83	15 minutes	6/10/2015 2:43 PM
84	10-15 min	6/10/2015 2:19 PM
85	10 minutes	6/10/2015 2:14 PM
86	20 minutes	6/10/2015 2:00 PM
87	20 minutes	6/10/2015 1:29 PM
88	20-30 minutes	6/10/2015 1:19 PM
89	15-20 minutes	6/10/2015 1:13 PM
90	20 minutes	6/10/2015 1:04 PM
91	15 minutes	6/10/2015 12:50 PM
92	15 min	6/10/2015 11:58 AM
93	10 minutes	6/10/2015 11:41 AM
94	10 min	6/10/2015 11:32 AM
95	10 mins	6/10/2015 11:26 AM
96	15 min	6/10/2015 11:25 AM
97	15 min	6/10/2015 10:26 AM
98	20	6/10/2015 9:14 AM
99	15 min	6/10/2015 9:06 AM
100	20 minutes	6/10/2015 8:58 AM
101	15 minutes	6/10/2015 8:37 AM
102	10 minutes	6/10/2015 8:08 AM
103	20	6/10/2015 7:53 AM
104	10-15 minutes (preferably 15 minutes)	6/10/2015 6:16 AM
105	15 minutes	6/10/2015 6:04 AM
106	20 min	6/10/2015 5:55 AM
107	10-15 mins	6/10/2015 1:54 AM

108	10-15 minutes	6/10/2015 12:39 AM
109	20 minutes	6/9/2015 10:57 PM
110	20 minutes	6/9/2015 10:28 PM
111	15 minutes	6/9/2015 9:56 PM
112	15-20 minutes	6/9/2015 9:26 PM
113	15-20 minutes	6/9/2015 8:46 PM
114	15 min	6/9/2015 8:03 PM
115	15min	6/9/2015 7:51 PM
116	5 to 15 min	6/9/2015 7:48 PM
117	15-20 minutes	6/9/2015 7:48 PM
118	10MIN	6/9/2015 7:17 PM
119	15 min.	6/9/2015 7:10 PM
120	3-5 min	6/9/2015 6:57 PM
121	10 minutes	6/9/2015 6:55 PM
122	15min.	6/9/2015 6:27 PM
123	20	6/9/2015 6:13 PM
124	15 minutes	6/9/2015 6:00 PM
125	15 min	6/9/2015 5:25 PM
126	15 min	6/9/2015 5:23 PM
127	15-30 minutes	6/9/2015 5:21 PM
128	one to five minutes	6/9/2015 5:20 PM
129	10 minutes	6/9/2015 5:08 PM
130	10 minutes	6/9/2015 5:03 PM
131	15-20	6/9/2015 5:00 PM
132	15 minutes	6/9/2015 4:55 PM
133	15 minutes	6/9/2015 4:53 PM
134	15 minutes	6/9/2015 4:47 PM
135	10 to 15 minutes	6/9/2015 4:41 PM
136	20 min	6/9/2015 4:24 PM
137	15 minutes	6/9/2015 4:06 PM
138	15 minutes	6/9/2015 4:03 PM
139	20 minutes	6/9/2015 3:56 PM
140	15-20	6/9/2015 3:44 PM
141	15 minutes	6/9/2015 3:41 PM
142	5-10 minutes	6/9/2015 3:38 PM
143	15	6/9/2015 3:32 PM
144	15 min	6/9/2015 3:27 PM
145	10-20 minutes	6/9/2015 3:25 PM

146	10-15 min	6/9/2015 3:22 PM
147	20 minutes	6/9/2015 3:16 PM
148	15 minutes	6/9/2015 3:10 PM
149	15 minutes	6/9/2015 3:08 PM
150	15 - 30 min	6/9/2015 3:01 PM
151	15 minutes	6/9/2015 2:55 PM
152	PFTs done immediately after the levalbuterol	6/9/2015 2:50 PM
153	10 min	6/9/2015 2:49 PM
154	15 minutes	6/9/2015 2:43 PM
155	15-20 minutes	6/9/2015 2:39 PM
156	20 minutes	6/9/2015 2:36 PM
157	10 minutes	6/9/2015 2:32 PM
158	15 minutes	6/9/2015 2:31 PM
159	10 minutes	6/9/2015 2:29 PM
160	10 minutes	6/9/2015 2:25 PM
161	15min	6/9/2015 2:23 PM
162	10 minutes	6/9/2015 2:22 PM
163	15 to 20 minutes	6/9/2015 2:15 PM
164	15 minutes	6/9/2015 1:58 PM
165	15 minutes	6/9/2015 1:58 PM
166	15 to 30 min	6/9/2015 1:48 PM
167	10 min	6/9/2015 1:44 PM
168	minimum 15 minutes	6/9/2015 1:40 PM
169	10 minutes	6/9/2015 1:38 PM
170	15-20min	6/9/2015 1:29 PM
171	20 mins	6/9/2015 1:27 PM
172	15 mins	6/9/2015 1:27 PM
173	15 minutes	6/9/2015 1:19 PM
174	20 minutes	6/9/2015 1:13 PM
175	20 minutes	6/9/2015 1:12 PM
176	15 minutes	6/9/2015 1:12 PM
177	15-20 minutes	6/9/2015 1:11 PM
178	10	6/9/2015 1:11 PM
179	10 minutes	6/9/2015 1:11 PM
180	At least 20 minnutes	6/9/2015 1:10 PM
181	depends- albuterol 15 minutes; ipratropium 30 min	6/9/2015 1:07 PM
182	15 min	6/9/2015 1:07 PM
183	15 minutes	6/9/2015 7:41 AM

184	20 min	6/9/2015 7:24 AM
185	10 mins	6/9/2015 4:56 AM
186	15 minutes	6/9/2015 1:59 AM
187	10-15 minutes	6/8/2015 8:34 PM
188	5 to 10 minutes	6/8/2015 1:36 PM
189	10 minutes	6/8/2015 12:10 PM
190	20 min	6/8/2015 11:34 AM
191	20 minutes	6/8/2015 7:42 AM
192	15-20 min	6/7/2015 9:04 PM
193	20 min	6/7/2015 4:35 PM
194	10-15 minutes	6/7/2015 3:19 PM
195	15 min	6/7/2015 2:48 PM
196	15 minutes	6/7/2015 2:37 PM
197	20 minutes	6/7/2015 1:33 PM
198	15 mi	6/7/2015 12:01 PM
199	20 minutes	6/6/2015 9:35 PM
200	15 min	6/6/2015 12:32 PM
201	15 minutes	6/6/2015 12:16 AM
202	15-20 min	6/5/2015 8:41 PM
203	15 minutes	6/5/2015 6:30 PM
204	15-20 min	6/5/2015 3:32 PM
205	15 minutes	6/5/2015 2:51 PM
206	10	6/5/2015 2:26 PM
207	15 min	6/5/2015 1:51 PM
208	20 minutes	6/5/2015 12:29 PM
209	20 min	6/5/2015 12:03 PM
210	10-15	6/5/2015 10:34 AM
211	15 minutes	6/5/2015 4:40 AM
212	20 minutes	6/4/2015 9:32 PM
213	10 - 15 min	6/4/2015 6:33 PM
214	10 minutes	6/4/2015 5:46 PM
215	3-5 min	6/4/2015 4:30 PM
216	15 mins	6/4/2015 4:28 PM
217	15min	6/4/2015 3:21 PM
218	10 minutes	6/4/2015 3:15 PM
219	20 min	6/4/2015 2:55 PM
220	7 min	6/4/2015 1:17 PM
221	10-15 minutes	6/4/2015 1:17 PM

222	15-20 min	6/4/2015 12:40 PM
223	na	6/4/2015 11:59 AM
224	15 min	6/4/2015 11:40 AM
225	15	6/4/2015 10:38 AM
226	10 minutes	6/4/2015 8:46 AM
227	15 to 20 minutes	6/4/2015 8:42 AM
228	20 min	6/4/2015 8:01 AM
229	15 minutes	6/4/2015 7:34 AM
230	20min	6/4/2015 7:33 AM
231	15 mins.	6/4/2015 7:17 AM
232	20 minutes	6/4/2015 7:15 AM
233	10 min	6/4/2015 5:50 AM
234	15 min	6/3/2015 10:11 PM
235	15 minutes	6/3/2015 9:43 PM
236	15 minutes	6/3/2015 9:37 PM
237	10 min	6/3/2015 8:23 PM
238	15 min	6/3/2015 8:17 PM
239	10 minutes	6/3/2015 7:56 PM
240	10 minutes	6/3/2015 7:45 PM
241	15 min	6/3/2015 7:35 PM
242	15 minutes	6/3/2015 6:48 PM
243	15 minutes	6/3/2015 5:09 PM
244	5-10 min	6/3/2015 4:55 PM
245	10 minutes	6/3/2015 4:54 PM
246	15 min	6/3/2015 4:42 PM
247	15-20 minutes	6/3/2015 4:37 PM
248	10 minutes	6/3/2015 4:14 PM
249	10 minutes	6/3/2015 3:37 PM
250	30 minutes	6/3/2015 3:33 PM
251	10-15 min	6/3/2015 3:06 PM
252	20 min	6/3/2015 2:43 PM
253	15 minutes	6/3/2015 2:16 PM
254	20 minutes	6/3/2015 2:08 PM
255	20 min	6/3/2015 1:59 PM
256	15min	6/3/2015 1:56 PM
257	20+ minutes	6/3/2015 1:10 PM
258	within 10 minutes	6/3/2015 1:10 PM
259	15 min	6/3/2015 1:06 PM

260	15 minutes	6/3/2015 12:56 PM
261	10 minutes	6/3/2015 12:49 PM
262	20 minutes	6/3/2015 12:13 PM
263	20 minutes	6/3/2015 12:07 PM
264	15 min	6/3/2015 11:46 AM
265	15 to 20 minutes	6/3/2015 11:45 AM
266	20-30 minutes	6/3/2015 11:27 AM
267	10-15 mins	6/3/2015 11:17 AM
268	15-20 minutes	6/3/2015 11:15 AM
269	5 mins	6/3/2015 11:06 AM
270	30 minutes	6/3/2015 11:03 AM
271	15 min	6/3/2015 10:50 AM
272	10 to 15 minutes	6/3/2015 10:35 AM
273	15 minutes	6/3/2015 10:34 AM
274	20 minutes	6/3/2015 10:26 AM
275	15 min	6/3/2015 10:21 AM
276	15 min	6/3/2015 10:18 AM
277	15 min	6/3/2015 10:17 AM
278	15 minutes	6/3/2015 10:11 AM
279	15-20 minutes	6/3/2015 10:10 AM
280	10 minutes	6/3/2015 10:08 AM
281	15 minutes	6/3/2015 9:59 AM
282	15 min	6/3/2015 9:48 AM
283	9 minutes	6/3/2015 9:31 AM
284	20 minutes	6/3/2015 9:28 AM
285	20 minutes	6/3/2015 9:21 AM
286	15 minutes	6/3/2015 9:11 AM
287	15	6/3/2015 8:58 AM
288	15-20 minutes	6/3/2015 8:43 AM
289	20 min	6/3/2015 8:40 AM
290	15minutes	6/3/2015 8:38 AM
291	15 minutes	6/3/2015 8:19 AM
292	15 minutes	6/3/2015 8:14 AM
293	20 minutes	6/3/2015 8:13 AM
294	15 minutes	6/3/2015 8:11 AM
295	15-20 minutes	6/3/2015 8:10 AM
296	15 MINUTES	6/3/2015 8:09 AM
297	20 min	6/3/2015 8:01 AM

298	15 minutes	6/3/2015 8:01 AM
299	20 minutes	6/3/2015 7:53 AM
300	15 mins.	6/3/2015 7:50 AM
301	10 minutes	6/3/2015 7:45 AM
302	15 minutes	6/3/2015 7:33 AM
303	15min	6/3/2015 6:39 AM
304	15 minutes	6/3/2015 6:31 AM
305	15 min	6/3/2015 6:23 AM
306	15 minutes	6/3/2015 6:05 AM
307	15 minutes	6/3/2015 5:49 AM
308	5 mins	6/3/2015 4:09 AM
309	15 minutes	6/3/2015 3:47 AM
310	10 min	6/3/2015 2:45 AM
311	10'	6/3/2015 1:04 AM
312	15 minutes	6/2/2015 11:59 PM
313	15-20 min	6/2/2015 11:11 PM
314	15 minutes	6/2/2015 11:02 PM
315	20	6/2/2015 10:51 PM
316	15	6/2/2015 10:43 PM
317	15 minutes	6/2/2015 10:37 PM
318	15 min	6/2/2015 10:24 PM
319	10 minutes	6/2/2015 10:18 PM
320	15 min	6/2/2015 9:59 PM
321	10	6/2/2015 9:59 PM
322	15	6/2/2015 9:52 PM
323	20 minutes	6/2/2015 9:49 PM
324	15 minutes	6/2/2015 9:39 PM
325	10 minutes	6/2/2015 9:39 PM
326	10 minutes	6/2/2015 9:30 PM
327	15 minutes	6/2/2015 9:26 PM
328	15 minutes	6/2/2015 9:20 PM
329	12-15min	6/2/2015 9:16 PM
330	15 minutes	6/2/2015 9:12 PM
331	10-15 minutes	6/2/2015 9:04 PM
332	10-15 minutes	6/2/2015 9:03 PM
333	15 minutes	6/2/2015 9:01 PM
334	15 min	6/2/2015 8:58 PM
335	15 minutes	6/2/2015 8:51 PM

336	15-20 min	6/2/2015 8:47 PM
337	20 minutes	6/2/2015 8:45 PM
338	5 to 15 minutes	6/2/2015 8:43 PM
339	15-20 minutes	6/2/2015 8:42 PM
340	15 minutes	6/2/2015 8:38 PM
341	20 min	6/2/2015 8:36 PM
342	15 minutes	6/2/2015 8:19 PM
343	15 minuted	6/2/2015 8:19 PM
344	15 minutes	6/2/2015 8:07 PM
345	20 minutes	6/2/2015 8:07 PM
346	10 minutes	6/2/2015 8:06 PM
347	20 minutes	6/2/2015 8:03 PM
348	Shortly after, immediate or within a few min	6/2/2015 7:59 PM
349	10 - 15	6/2/2015 7:57 PM
350	15-20	6/2/2015 7:56 PM
351	10 min	6/2/2015 7:47 PM
352	15 minutes	6/2/2015 7:43 PM
353	20 minutes	6/2/2015 7:42 PM
354	20 min	6/2/2015 7:31 PM
355	15 min	6/2/2015 7:25 PM
356	15 minutes	6/2/2015 7:13 PM
357	10 minutes	6/2/2015 7:10 PM
358	5 minutes (longer if time allows)	6/2/2015 7:07 PM
359	12-15 min	6/2/2015 7:07 PM
360	12-15 minutes	6/2/2015 7:04 PM
361	15 min	6/2/2015 7:03 PM
362	15 minutes	6/2/2015 7:03 PM
363	15 minutes	6/2/2015 6:57 PM
364	15 min	6/2/2015 6:56 PM
365	15 minutes	6/2/2015 6:42 PM
366	10 to 15 minutes	6/2/2015 6:42 PM
367	20 minutes	6/2/2015 6:30 PM
368	20 minutes	6/2/2015 6:28 PM
369	15 minutes	6/2/2015 6:22 PM
370	10 min	6/2/2015 6:18 PM
371	3 minutes	6/2/2015 6:14 PM
372	10 min	6/2/2015 6:13 PM
373	15-20 minutes	6/2/2015 6:11 PM

374	15 min	6/2/2015 6:10 PM
375	5-7.	6/2/2015 6:03 PM
376	15 to 20 minutes	6/2/2015 5:54 PM
377	10-20 min	6/2/2015 5:51 PM
378	at least 15 minutes	6/2/2015 5:49 PM
379	15	6/2/2015 5:47 PM
380	15 minutes	6/2/2015 5:47 PM
381	at least 20 minutes	6/2/2015 5:40 PM
382	15 min	6/2/2015 5:38 PM
383	15 minutes	6/2/2015 5:36 PM
384	15-20 minutes	6/2/2015 5:36 PM
385	15 minutes	6/2/2015 5:35 PM
386	20 minutes	6/2/2015 5:32 PM
387	20 minutes	6/2/2015 5:31 PM
388	10 minutes	6/2/2015 5:30 PM
389	20 minutes	6/2/2015 5:29 PM
390	15 minutes	6/2/2015 5:22 PM
391	15 minutes	6/2/2015 5:19 PM
392	20 min	6/2/2015 5:09 PM
393	15 minutes	6/2/2015 5:09 PM
394	20 minutes	6/2/2015 5:09 PM
395	10	6/2/2015 5:08 PM
396	15min	6/2/2015 5:06 PM
397	15 min	6/2/2015 5:05 PM
398	10 minutes	6/2/2015 5:00 PM
399	10 minutes	6/2/2015 4:54 PM
400	15	6/2/2015 4:54 PM
401	20	6/2/2015 4:49 PM
402	10-15 minutes	6/2/2015 4:46 PM
403	10-15 min	6/2/2015 4:43 PM
404	30 minutes	6/2/2015 4:42 PM
405	20 min	6/2/2015 4:37 PM
406	15'	6/2/2015 4:32 PM
407	10 minutes	6/2/2015 4:30 PM
408	15 min	6/2/2015 4:24 PM
409	10 minutes	6/2/2015 4:23 PM
410	10 minutes	6/2/2015 4:18 PM
411	5 minutes	6/2/2015 4:18 PM

412	3 minutes	6/2/2015 4:17 PM
413	15 minutes	6/2/2015 4:16 PM
414	15 minutes	6/2/2015 4:16 PM
415	15-20 minutes	6/2/2015 4:15 PM
416	15 min	6/2/2015 4:10 PM
417	15-20 min	6/2/2015 4:09 PM
418	10 minutes	6/2/2015 4:08 PM
419	15 min	6/2/2015 4:08 PM
420	15 min	6/2/2015 4:08 PM
421	5 - 10 minutes	6/2/2015 4:08 PM
422	15 minutes	6/2/2015 4:05 PM
423	20 minutes	6/2/2015 4:05 PM
424	20 minutes	6/2/2015 4:04 PM
425	15 minutes	6/2/2015 4:02 PM
426	10 min	6/2/2015 4:02 PM
427	15 minutes	6/2/2015 4:01 PM
428	15 minutes	6/2/2015 4:01 PM
429	10 minutes	6/2/2015 4:00 PM
430	15 min	6/2/2015 3:59 PM
431	15 minutes	6/2/2015 3:58 PM
432	15-20 mins	6/2/2015 3:57 PM
433	20 minutes	6/2/2015 3:56 PM
434	15-20 minutes	6/2/2015 3:56 PM
435	15	6/2/2015 3:56 PM
436	15 minutes	6/2/2015 3:55 PM
437	10 minutes	6/2/2015 3:54 PM
438	10 minutes	6/2/2015 3:54 PM
439	5 minutes	6/2/2015 3:52 PM
440	15 min	6/2/2015 3:50 PM
441	15 min	6/2/2015 3:50 PM
442	10 min	6/2/2015 3:50 PM
443	15 minutes	6/2/2015 3:49 PM
444	5 minutes	6/2/2015 3:48 PM
445	20 minutes	6/2/2015 3:46 PM
446	15 min	6/2/2015 3:45 PM
447	10-15 mins	6/2/2015 3:42 PM
448	15	6/2/2015 3:41 PM
449	10 min	6/2/2015 3:41 PM

450	15-20 minutes	6/2/2015 3:40 PM
451	15 minutes	6/2/2015 3:40 PM
452	15 minutes	6/2/2015 3:38 PM
453	20 minutes	6/2/2015 3:37 PM
454	15 min	6/2/2015 3:37 PM
455	15 min	6/2/2015 3:37 PM
456	10 min	6/2/2015 3:35 PM
457	10-15 minutes	6/2/2015 3:35 PM
458	20 minutes	6/2/2015 3:35 PM
459	30 min	6/2/2015 3:34 PM
460	20 min	6/2/2015 3:32 PM
461	10-15 minutes	6/2/2015 3:31 PM
462	20 minutes	6/2/2015 3:31 PM
463	15 minutes	6/2/2015 3:31 PM
464	20 minutes	6/2/2015 3:31 PM
465	10 minutes	6/2/2015 3:30 PM
466	5 minutes	6/2/2015 3:30 PM
467	15 minutes	6/2/2015 3:30 PM
468	15 minutes	6/2/2015 3:29 PM
469	10 mins	6/2/2015 3:29 PM
470	15 minutes	6/2/2015 3:28 PM
471	20 minutes	6/2/2015 3:28 PM
472	15 min.	6/2/2015 3:27 PM
473	10 minutes	6/2/2015 3:27 PM
474	15-20 minutes	6/2/2015 3:27 PM
475	15 miin	6/2/2015 3:26 PM
476	15	6/2/2015 3:25 PM
477	15 or 30 minutes depending on agent	6/2/2015 3:25 PM
478	15 minutes	6/2/2015 3:24 PM
479	15 min	6/2/2015 3:23 PM
480	10	6/2/2015 3:23 PM
481	10 minutes	6/2/2015 3:23 PM
482	10 minutes	6/2/2015 3:23 PM
483	20	6/2/2015 3:23 PM
484	15 min	6/2/2015 3:23 PM
485	15-20 min	6/2/2015 3:23 PM
486	15 to 20 minutes	6/2/2015 3:22 PM
487	15 minutes	6/2/2015 3:21 PM

Q6 Do you think your asthma medication administration is sterile?

Answered: 491 Skipped: 5

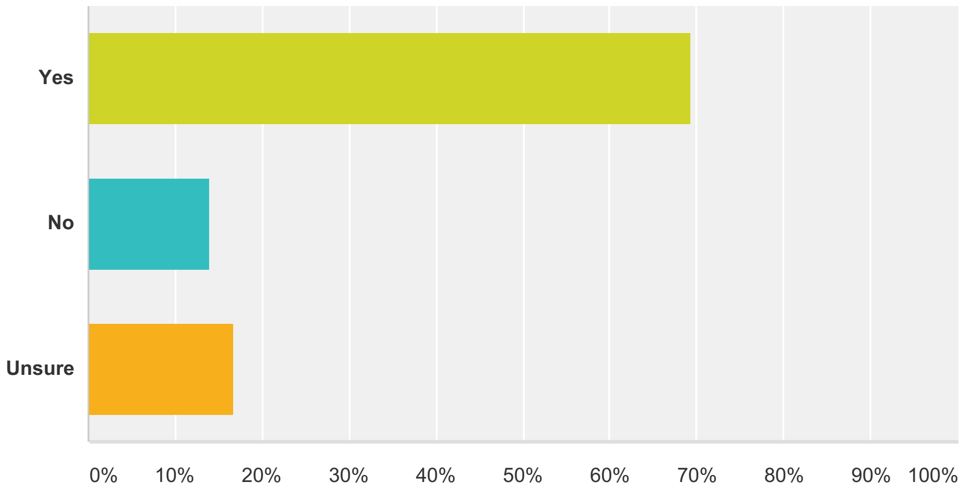

Answer Choices	Responses	
Yes	**69.25%**	340
No	**14.05%**	69
Unsure	**16.70%**	82
**Total**		**491**

Q7 How do you maintain inhaler sterility between each patient?

Answered: 449 Skipped: 47

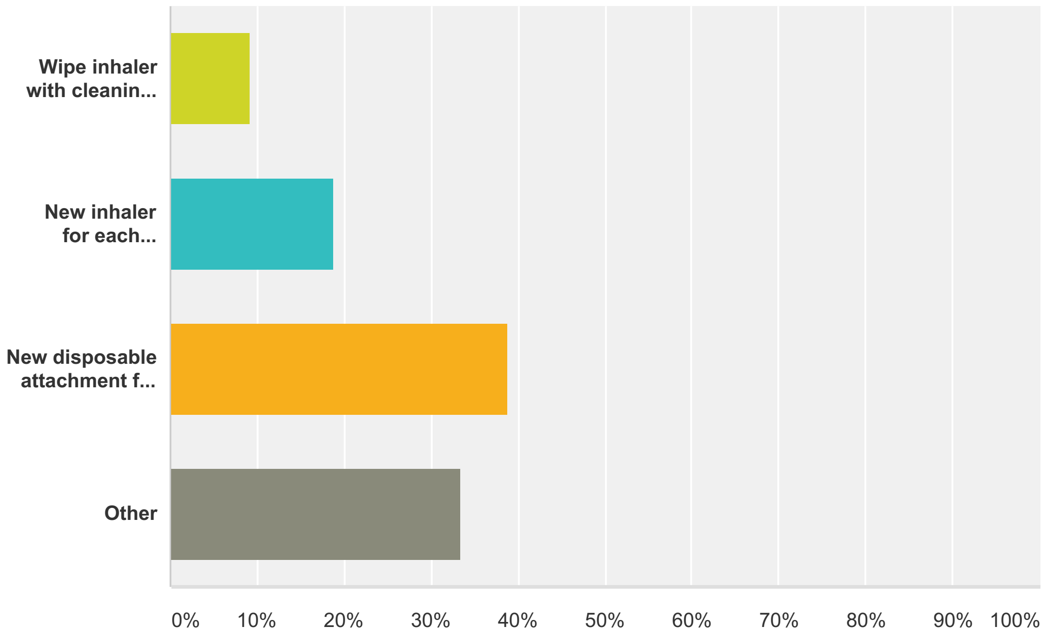

Answer Choices	Responses	
Wipe inhaler with cleaning agent	**9.13%**	41
New inhaler for each patient	**18.71%**	84
New disposable attachment for reused inhaler	**38.75%**	174
Other	**33.41%**	150
**Total**		**449**

#	If you chose a new inhaler or Other, please elaborate:	Date
1	Out the mouth technique	6/19/2015 4:48 PM
2	use spacer that is sterilized after each use	6/18/2015 5:24 PM
3	inhaler is wiped with cleaning agent and a new spacer is used with each pt	6/18/2015 4:49 PM
4	use nebulizer with new tubing and mouthpiece for every patient	6/18/2015 12:03 PM
5	We use spacers for each patient	6/18/2015 9:56 AM
6	Each patient receives a new nebulizer tubing with mouthpiece from a sealed package. The albuterol 0.5% nebulizer solution comes from a multi-dose 20 mL bottle; the saline or ipratropium comes from a sealed vial. So, not truly sterile, but it is kept as clean as reasonably possible.	6/17/2015 2:37 PM
7	cleaned spacer	6/17/2015 9:43 AM
8	Use new blue extension tube with each patient	6/16/2015 9:38 PM
9	soak the mouth piece or spacer in disinfectant solution	6/16/2015 8:33 PM

10	DO NOT USE AN INHALER.....THE DELIVERY IS ONLY AS GOOD AS THEY PATIENT'S EFFORT AND TECHNIQUE WHICH MEANS THERE IS NO CONSISTENCY BETWEEN TESTS DONE AT DIFFERENT VISITS.....A NEBULIZER IS NOT PATIENT TECHNIQUE DEPENDENT SO THE DELIVERY IS THE SAME EVERY TIME....AND THERE'S NO STERLILITY ISSUE	6/16/2015 8:00 PM
11	We use nebulizer with fresh tubing	6/16/2015 6:00 PM
12	Use disposable nebulizer	6/16/2015 2:00 PM
13	We don't use inhalers for this purpose.	6/16/2015 1:22 PM
14	New tubing and mouthpiece for each patient for nebulizer	6/16/2015 1:01 PM
15	Soak the outer plastic holder of an inhaler in sterilizing solution. We do not put the pressurized canister in the solution	6/16/2015 12:55 PM
16	use new nebulizer tubing	6/16/2015 12:42 PM
17	Soap and water	6/16/2015 12:22 PM
18	New nebulizer mouth piece or mask for each patient.	6/16/2015 11:36 AM
19	patient inhaler or office sample	6/16/2015 11:33 AM
20	Sterilize outer detachment between uaea	6/16/2015 11:00 AM
21	I have the patient use their own inhaler or I get one from the sample cabinet.	6/16/2015 10:38 AM
22	New attachement to the nebulizer for every patient tested.	6/16/2015 10:14 AM
23	Don't use inhalers	6/16/2015 9:30 AM
24	do not used the same inhaler for different patients	6/16/2015 9:23 AM
25	Don't use inhalers. I use nebs. Inhalers are not labelled for multiple patient use anyway.	6/16/2015 9:12 AM
26	New nebulizer device for each patient	6/16/2015 9:09 AM
27	New inhaler or new tubing circuit for nebulizer	6/16/2015 9:09 AM
28	We use a cardboard tubes disposed off after single use.	6/16/2015 9:06 AM
29	Use a clean spacer cleaned between each patient.	6/15/2015 8:48 PM
30	New disposable attachment for reused spacer, and sterilize spacer	6/15/2015 8:44 PM
31	remove canister. wipe (usually) or soak in cleaning agent then rinse the plastic component. let dry thoroughly. wait 24 hrs. or more for reuse (to make sure all is dry). spacer is used for those with poor technique.	6/14/2015 7:25 PM
32	spacers and nebulizer attachment (minus tubing) are soaked overnight in sterilizing solution	6/14/2015 4:52 PM
33	New mobilizer tubing	6/14/2015 1:43 AM
34	New nebulizers set up and wipe down of unit.	6/12/2015 10:34 AM
35	neb	6/12/2015 9:05 AM
36	We use a new nebulizer set up and unit dose albuterol or levalbuterol for each patient	6/12/2015 7:51 AM
37	have the patinet's bring their own inhaler	6/11/2015 11:34 AM
38	chamber is sterilized and inhaler is wiped	6/10/2015 5:29 PM
39	We use nebulizer	6/10/2015 2:53 PM
40	na	6/10/2015 2:19 PM
41	we use a nebulizer	6/10/2015 1:29 PM
42	Everything used for nebulization is new for each patient.	6/10/2015 9:48 AM
43	Cleaned, dried spacer for each test.	6/10/2015 9:14 AM
44	just stopped using spacers which we used to wipe clean. Now just use a nebulizer to maintain increased sterility	6/10/2015 9:06 AM

45	n/a	6/10/2015 9:03 AM
46	questions 6 & 7 are misleading. The mouthpiece is clean, but not sterile, any more than a drinking cup would be. However, the medication itself is sterile.	6/10/2015 8:58 AM
47	We don't use MDI's for this reason. We use nebulizer with new tubing.	6/10/2015 8:37 AM
48	neb device	6/10/2015 8:08 AM
49	Usually use a nebulizer to demonstrate reversibility. If using an MDI usually a sample is provided or use patients if they have one.	6/10/2015 12:39 AM
50	Sterilize nebulizer equipment	6/9/2015 10:57 PM
51	I use disposable nebulizers on all patients. I have no idea if they are sterile or not. They are not sterile when I touch the nebulizer b/c I don't use sterile gloves.	6/9/2015 9:26 PM
52	samples	6/9/2015 8:46 PM
53	Patients are instructed to bring their own devices or the post-BD PFTs are not done...	6/9/2015 8:03 PM
54	wavicide atttachment	6/9/2015 6:00 PM
55	new mask for each patient	6/9/2015 5:25 PM
56	This can never be sterile in the sense of autoclaving, but the chamber is cleaned and air dried between patients.	6/9/2015 5:23 PM
57	new individually packaged pipe or mask delivery system using sterile unit doses of nebulized medication	6/9/2015 5:20 PM
58	patients own inhaler	6/9/2015 5:00 PM
59	new nebulizer tubing, cup, etc. for each tested patient	6/9/2015 4:55 PM
60	New nebulizer circuit including hose for each patient	6/9/2015 4:47 PM
61	use nebulier	6/9/2015 4:41 PM
62	sterilizable plastic cup and mouthpiece	6/9/2015 4:24 PM
63	Do not use inhaler	6/9/2015 3:56 PM
64	new nebulizer kit for each patient	6/9/2015 3:38 PM
65	we uses sterilised commercial MDI plastic containers; ie the blue plastic tubes that albuterol is marketed with.	6/9/2015 3:27 PM
66	We clean spacer in between patients	6/9/2015 3:22 PM
67	New nebulizer cup with each patient	6/9/2015 3:10 PM
68	Use sterile nebulizer equipment	6/9/2015 2:50 PM
69	new disposable nebulizer set up for each patient	6/9/2015 2:43 PM
70	Inhalers/spacers are soaked in sterilizing solution.	6/9/2015 2:39 PM
71	ampules	6/9/2015 2:36 PM
72	Sporox disinfectant	6/9/2015 2:25 PM
73	use neb machine with new buting and mask and/or mouthpiece	6/9/2015 2:22 PM
74	Disinfect with hoSPITAL GRADE CLEANER	6/9/2015 1:58 PM
75	New nebulizer cups, tubing, sterile wipe down of compressor	6/9/2015 1:58 PM
76	use neb with disposable tubing	6/9/2015 1:38 PM
77	Glutaraldehyde cleaned Neb Chamber and tubing	6/9/2015 1:27 PM
78	Use new nebulizer unit for each patient	6/9/2015 1:12 PM
79	Soak MDI housing in microbicidal wash.	6/9/2015 1:11 PM
80	Use nebuliser New tubing for each patient	6/9/2015 1:11 PM

81	I don't share inhalers between people	6/9/2015 1:10 PM
82	Unknown; however this is part of the reason I do it in RT department->quality control is better.	6/9/2015 7:24 AM
83	clean spacer	6/9/2015 4:56 AM
84	clean in Cidex	6/9/2015 1:59 AM
85	I use samles	6/8/2015 1:36 PM
86	We used to clean our spacers with Cidex. We just stopped doing that. We now ask our patients to bring their own spacers to the office or use a disposable nebulizer.	6/7/2015 9:04 PM
87	Different neb mouthpiece	6/7/2015 12:01 PM
88	New disposable mouthpiece and tubing with single dose sterile albuterol vial	6/6/2015 12:32 PM
89	New spacer with 1 way valve for each patient. We re-use the albuterol with attachment unit.	6/5/2015 2:26 PM
90	new nebulizer	6/5/2015 12:03 PM
91	each patient uses their own inhaler that they bring to the office	6/5/2015 10:34 AM
92	Use nebulizer	6/5/2015 4:40 AM
93	One of the main reasons we use a nebulizer is so that we can sterilize	6/4/2015 6:33 PM
94	We use one time nebulizer, mouthpiece/mask and tubing for each pt.	6/4/2015 1:17 PM
95	disposable attachment for spacer; spacer wiped with alcohol between patients, and reused inhaler	6/4/2015 12:40 PM
96	na	6/4/2015 11:59 AM
97	we do NOT use an inhaler, rather we use NEW nebulizer tubing and equipment between ALL patients, for this reason, we STRONGLY believe this is NEEDED for patient safety	6/4/2015 8:46 AM
98	Nebulizer only	6/4/2015 8:01 AM
99	New tubing and mouthpiece with each nebulization	6/4/2015 7:15 AM
100	New sterile disposable nebulizer tubing, mouthpiece, and cup for each patient	6/3/2015 9:37 PM
101	chemical sterilization of spacer and mdi	6/3/2015 7:35 PM
102	use only nebulizer. No repeat tubing, etc	6/3/2015 4:55 PM
103	Use individual vials of brochodilator with disposable nebulizing units.	6/3/2015 4:37 PM
104	Soak in Sporox.	6/3/2015 3:37 PM
105	Plastic inhaler piece and spacer (if used) soaked in bleach solution after each use. medication canister removed from plastic inhaler piece and used in a clean plastic piece for next patient	6/3/2015 1:59 PM
106	New nebulizer administration set.	6/3/2015 1:10 PM
107	disposable nebulizer tubing and bulb	6/3/2015 1:06 PM
108	patient uses their own inhaler	6/3/2015 12:49 PM
109	New nebulizer attachment made for the Pari.	6/3/2015 12:13 PM
110	new nebulizer tubing for each patient	6/3/2015 11:45 AM
111	rinse mouthpiece with soap and water	6/3/2015 11:17 AM
112	Use nebulizer machine unless patient has their own rescue inhaler, then use their rescue inhaler.	6/3/2015 11:15 AM
113	sterilize attachment for reuse	6/3/2015 10:50 AM
114	We use a whole new nebulizer kit per patient--tubing, nebulizer, mask/mouthpiece--they are never re-used for another patient	6/3/2015 10:35 AM
115	Don't use mdi - use a new neb set.	6/3/2015 10:18 AM
116	replace spacer device (AeroChamber, one way valve)	6/3/2015 10:17 AM

117	New nebulizer tube and mask	6/3/2015 10:11 AM
118	wash plastic housing	6/3/2015 9:48 AM
119	A spacer is always used with the inhaler and that is sterilized after each use	6/3/2015 9:31 AM
120	All of the above.	6/3/2015 9:21 AM
121	Sterilization with cidex	6/3/2015 8:58 AM
122	Use nebulizer with new tubing for each patient	6/3/2015 8:14 AM
123	Up to now I have provided patients with a sample of either proair or ventolin, or more rarely xopenex. The trend recently I am experiencing is that I will not have enough samples to continue. I am considering alternatives.	6/3/2015 8:13 AM
124	We don't use an inhaler. We use nebulizer with new supplies for each patient.	6/3/2015 8:09 AM
125	nebulizer	6/3/2015 8:01 AM
126	we use a solution to put the MDI or spacer into to sterilize	6/3/2015 8:01 AM
127	We sterilize the spacers in cidex between pts	6/3/2015 7:45 AM
128	bacteriacidal cleaning of each MDI holder , and disposable spacer used with it	6/3/2015 6:39 AM
129	I don't use inhaler for this test	6/3/2015 6:23 AM
130	cidex overnite	6/3/2015 4:09 AM
131	Use Nebs only	6/3/2015 2:45 AM
132	New neb, tubing, and mouthpiece or mask for each patient	6/3/2015 1:04 AM
133	nebulizer has new tubing and mask for each patient	6/2/2015 11:59 PM
134	Do not use an inhaler	6/2/2015 10:18 PM
135	Use disposable nebulizer tubing. Don't use inhaler.	6/2/2015 9:59 PM
136	We don't use inhalers	6/2/2015 9:59 PM
137	We soak the mouthpiece in a cleaner	6/2/2015 9:52 PM
138	we have new inhalers available for use then the patient takes it home.	6/2/2015 9:49 PM
139	do not use inhalers	6/2/2015 9:30 PM
140	Either the patient uses their inhaler or I use a new sample. If I use a nebulizer, I use a new tubing kit and a vial of sterile solution	6/2/2015 9:20 PM
141	disposable neb cups	6/2/2015 9:16 PM
142	We use a nebulizer	6/2/2015 9:04 PM
143	new tubing and nebulizer	6/2/2015 9:03 PM
144	New nebulizer cup per pt. Unit doses of albuterol per pt.	6/2/2015 8:58 PM
145	I use a nebuliser so all the tubing etc is disposable	6/2/2015 8:47 PM
146	Nebulizer kit for every patient	6/2/2015 8:42 PM
147	Use a nebulization primarily	6/2/2015 8:38 PM
148	Soak inhaler mouthpiece (not canister) and spacer in cleaning agent designed to sterilize	6/2/2015 8:19 PM
149	we wash our nebulizers and MDI's in a cleaning solution, let them air dry.	6/2/2015 8:06 PM
150	Nebs	6/2/2015 7:59 PM
151	New tubing per patient	6/2/2015 7:42 PM
152	Autoclave	6/2/2015 7:31 PM

153	All patients are advised to bring there inhalers and holding chamber in each office visit. Patient's inhaler and holding chamber is used for the test.	6/2/2015 7:13 PM
154	new nebulizer set up or soak plastic 24 hours in sterilization solution	6/2/2015 7:10 PM
155	Cidex soak for mouthpieces	6/2/2015 7:07 PM
156	Soak in antiseptic	6/2/2015 7:07 PM
157	All new mouth piece tubing and meds for each patient	6/2/2015 7:03 PM
158	Nebulizer mouthpiece switched out.	6/2/2015 7:03 PM
159	Aero chamber used for inhaler is sterilizaed	6/2/2015 6:42 PM
160	Often the patient has their own or we use samples, if not available then we use the neb soln.	6/2/2015 6:42 PM
161	New disposable attachment for reused inhaler -- ** We use Lite-Aire disposable spacers.	6/2/2015 6:22 PM
162	in addition one time use of filter piece in nebulizer	6/2/2015 6:18 PM
163	99% of the time we nebulize it with disposable nebulizer supplies; the other 1% of the time we use a sample of the albuterol MDI and send it home with the patient; either way both are sterile	6/2/2015 6:14 PM
164	Only use Patient's own inhaler	6/2/2015 5:38 PM
165	Néw nebula and tubing.	6/2/2015 5:36 PM
166	same inhaler, sterilized valved holding chamber	6/2/2015 5:35 PM
167	New tubing when Neb used	6/2/2015 5:29 PM
168	Use samples	6/2/2015 5:22 PM
169	soaking in sterilizing soln	6/2/2015 4:54 PM
170	soak all used MDI attachments overnight in dilute bleach solution	6/2/2015 4:49 PM
171	Use only neb with new kit, rarely use albuterol HFA (a new sample with a spacer)	6/2/2015 4:43 PM
172	Always use valved holding chamber that is washed and disinfected after each use. The MDI's are never put in patient's mouths.	6/2/2015 4:42 PM
173	I only use nebulizer with new tubing for each patient.	6/2/2015 4:30 PM
174	saok neb. chamber in disinfectant	6/2/2015 4:24 PM
175	New mouthpiece for each IH rx	6/2/2015 4:18 PM
176	use disposable nebulizer	6/2/2015 4:18 PM
177	Sterilized nebulizer and tubing.	6/2/2015 4:16 PM
178	We used nebulozed ttm. All the time separate tubing and mask	6/2/2015 4:15 PM
179	Soak in disinfectant solution	6/2/2015 4:08 PM
180	Soak spacers in bleach water 60 min, rinse, and air dry	6/2/2015 4:08 PM
181	We use a nebulizer	6/2/2015 4:08 PM
182	new inhaler if MDI used. Disposable kit if nebulizer used.	6/2/2015 4:05 PM
183	Disposable mouth piece with filter	6/2/2015 4:02 PM
184	We always use a spacer and it is disinfected after each use	6/2/2015 3:58 PM
185	new nebulizer tubing/mask for each patient	6/2/2015 3:56 PM
186	cleansed spacer	6/2/2015 3:54 PM
187	It is prepackaged	6/2/2015 3:54 PM
188	New T-piece neb for each patient.	6/2/2015 3:52 PM

189	No inhalers used	6/2/2015 3:50 PM
190	new nebulizer tubing and mouth piece	6/2/2015 3:50 PM
191	new nebulizer and tubing for each patient	6/2/2015 3:49 PM
192	New nebulizer kit for each patient	6/2/2015 3:48 PM
193	Sterilize nightly and dishwash weekly	6/2/2015 3:41 PM
194	nebulizer equipment is clean and sealed	6/2/2015 3:37 PM
195	disposable neb units	6/2/2015 3:37 PM
196	Soak in Sklar	6/2/2015 3:35 PM
197	spacer used cleaned and inhalers in each exam room	6/2/2015 3:35 PM
198	I give them samples of new ones.	6/2/2015 3:31 PM
199	Use a disposable cardboard attachment on end of spacer.	6/2/2015 3:31 PM
200	Only do nebulizer with new disposable neb chamber	6/2/2015 3:31 PM
201	Do not use inhaler. Use nebulizer only and dispose of tubing and mouthpiece after each use.	6/2/2015 3:31 PM
202	Clean spacer mouthpiece after each use	6/2/2015 3:29 PM
203	cleanse	6/2/2015 3:28 PM
204	Samples of new inhalers from pharma reps.	6/2/2015 3:24 PM
205	new neb unit for each pt	6/2/2015 3:23 PM
206	Same MDI. Chamber washed and cleaned with hot soapy water between patients. Disposable mouthpiece.	6/2/2015 3:23 PM

Q8 Do you measure fractional exhaled Nitric Oxide (FeNO) as part of your assessment of reversibility?

Answered: 485 Skipped: 11

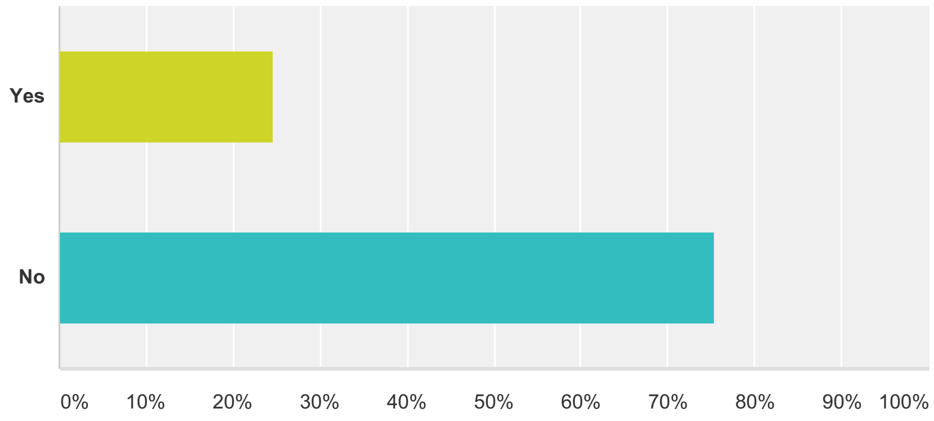

Answer Choices	Responses	
Yes	**24.54%**	119
No	**75.46%**	366
**Total**		**485**

Q9 How do you interpret a positive test?

Answered: 475 Skipped: 21

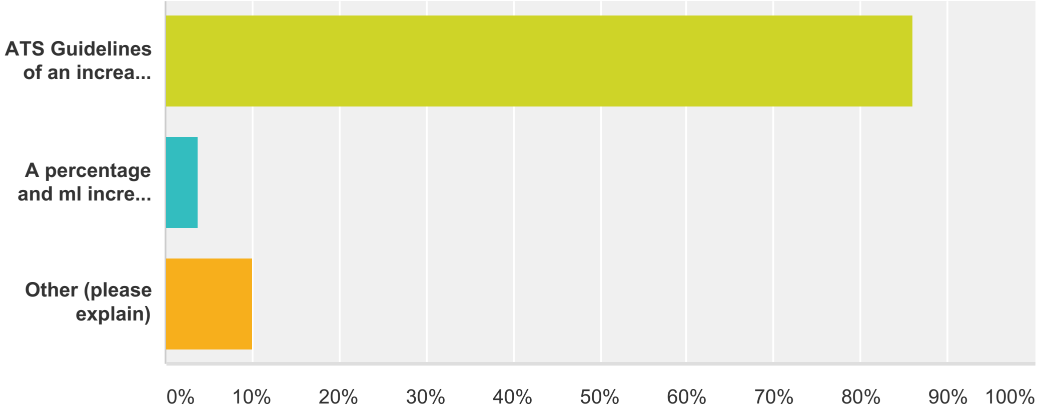

Answer Choices	Responses	
ATS Guidelines of an increase of 12% and 200 ml in FEV1 or FVC over the baseline	**86.11%**	409
A percentage and ml increase in FEV1 or FVC over the baseline	**3.79%**	18
Other (please explain)	**10.11%**	48
**Total**		**475**

#	Other (please explain)	Date
1	FEV1 increase 15% (prefer 20%) and/or FEF 25-75 increase 25% over baseline	6/18/2015 12:03 PM
2	ATS Guidelines of an increase of 12% and 20ml in FEV1 or FVC over baseline..........I ALSO LOOK AT THE FEF25% - 75% FOR THE SAME 12% INCREASE AS THESE ARE THE TERMINAL BRONCHIOLES AND ALVEOLI WHERE OXYGEN IS DELIVERED TO THE BLOOD....DAILY I SEE THIS IMPROVE IN PATIENTS WHICH IS CONSISTENT WITH THEIR COMMENT THEY CAN NOW FILL THEIR LUNGS AND BREATHE MUCH DEEPER AND ALSO CONSISTENT WITH AN INCREASE IN AIR EXCHANGE IN BASES ON AUSCULTATION....THERE MAY OR MAY NOT BE A CHANGE IN FEV1 OR FVC BUT PATIENT REPORTS AND AUSCULTATION CONFIRMS THE CHANGE OF FEF25% - 75% EVERY TIME	6/16/2015 8:00 PM
3	At least 12 %	6/16/2015 7:01 PM
4	12-15% positive change in FEV1 and FEF 25-75% as well as FVC	6/16/2015 1:22 PM
5	ATS criteria plus clinical history and physical	6/16/2015 1:01 PM
6	12-15+ %.	6/16/2015 12:22 PM
7	I use a change in just FEV1 of 12% OR 200 ml. I don't require both 12% AND 200 ml.	6/16/2015 9:30 AM
8	I see only children and ATS criteria are used for those of adult stature. Otherwise at least 10% increase in FEV 1	6/16/2015 9:09 AM
9	1/2 my patients are children. of the adults, some are elderly--i do NOT expect a 5' elderly lady with an FVC of 1 liter to have increase of 200 ml --which would be 20% improvement-- in order to see significant reversibility.	6/14/2015 7:25 PM
10	I look at % increase in FEV1 as well as changes in FEF25-75 and volume changes. The magnitude of FEV1 increase that's significant really depends upon the age and size of the patient. 200 ml increase in a 7 year old is not a realistic criterion for a positive test, but is more predictive in a 40 year old man.	6/10/2015 8:58 AM
11	15% and 200ml	6/9/2015 7:48 PM
12	Since I deal with children, a 200ml increase in FEV1 may not be a relevant number. I use a 12% increase in FEV1 and a 20% increase in FEF 25-75% for diagnostic purposes.	6/9/2015 6:13 PM

13	ATS guidelines, but another important measure of reversibility if variability among different test dates or on a different date soon after starting inhaled or systemic steroids, etc.	6/9/2015 5:23 PM
14	Increase in fef 25-75 or fev 1	6/9/2015 4:47 PM
15	In pediatrics we look for at least an 8% change in fev1	6/9/2015 3:44 PM
16	the ATS criteria are "significant" but by no means "diagnostic" of asthma.	6/9/2015 3:27 PM
17	Both FEV1 and FVC increase of 12% and 200 mL	6/9/2015 3:01 PM
18	I have always gone with 15%change	6/9/2015 2:50 PM
19	Increase in FEV1 of 12% and 200 ml over baseline	6/9/2015 2:43 PM
20	12% and 200 ml in FEV1	6/9/2015 1:12 PM
21	No clear definition, interpret overall and in light of patients symptoms	6/8/2015 7:42 AM
22	We typically use a % over baseline (around 12%) as we see many pediatric patients	6/7/2015 9:04 PM
23	I am very confused about the ATS guidelines of an increase of 12% as it seems that MDs interpret this differently. My interpretation: if a person has an FEV1 of 75% predicted, FEV1 would have to rise to 87% of predicted to be a 12% increase. I believe that others use a 12% rise in absolute FEV1, which is different than a 12% increase in % predicted FEV1.	6/6/2015 12:16 AM
24	changes in FEV1 and FEF 25-75	6/4/2015 4:30 PM
25	15% increase FEV1 and/or FEF 25-75	6/4/2015 7:33 AM
26	depends on age of patient. 200 mL not relevant to most 3 or 4 year olds	6/3/2015 2:16 PM
27	Generally use the 12% change in FEV-1 more than FVC.	6/3/2015 1:10 PM
28	20% increase in FEV1 over baseline	6/3/2015 11:27 AM
29	CHILDREN....percentage increase.....should we not pay attention to our Pediatric population?	6/3/2015 10:17 AM
30	ATS guidelines for adults and FMEF for children	6/3/2015 9:48 AM
31	ATS as above or 25% increase in FEF25-75%	6/3/2015 8:58 AM
32	Increase of >12% in FEV1, or >25% in FEF 25-75%	6/3/2015 8:13 AM
33	percentage	6/2/2015 9:30 PM
34	Increase in FEF 25-75% by greater than 25%	6/2/2015 8:43 PM
35	A, B or sometimes patient subjective improvement after bronchodilator.	6/2/2015 7:07 PM
36	I use a percentage (>12-15%) increase but do not always require a 200 ml increase as well.	6/2/2015 7:04 PM
37	Or clinical improvement ie: cough wheezing improved 12 to 15 percent	6/2/2015 7:03 PM
38	Ats as above. But not necessarily for kids.	6/2/2015 6:42 PM
39	I use the FEV1 as above and the FEF25-75	6/2/2015 6:13 PM
40	10-12% and 200 ml	6/2/2015 5:47 PM
41	first answer above(ATS guidelines) orFEF 25-75% increase equal or greater than 25%	6/2/2015 5:29 PM
42	change in mid-flow	6/2/2015 4:16 PM
43	Clinical history if reversibility not achieved	6/2/2015 3:54 PM
44	10% improvement and also in kids FEF 25-75% ratio is particularly helpful but also adhere to ATS guidelines MOST of the time	6/2/2015 3:35 PM
45	10 % improvement and meet the 200 cc minimum requirement	6/2/2015 3:31 PM
46	12% increase over baseline or at least 200 ml increase over baseline	6/2/2015 3:31 PM
47	As first answer plus consider 8-9% potentially significant adolescents	6/2/2015 3:27 PM

48	Use ATS guidelines but may suggest possibility of reversibility if exact criteria are not met.	6/2/2015 3:25 PM

Q10 What is the percentage and ml increase of FEV1 or FVC over the baseline in a positive test?

Answered: 384 Skipped: 112

#	Responses	Date
1	12%	6/19/2015 4:48 PM
2	12% or 200 ml	6/19/2015 12:50 PM
3	12%/200 ml	6/19/2015 12:32 PM
4	12% or 200ml	6/18/2015 5:24 PM
5	12% and 200ml	6/18/2015 4:49 PM
6	see above	6/18/2015 12:03 PM
7	12% and 200 mL	6/18/2015 9:56 AM
8	12	6/17/2015 4:53 PM
9	Same as in ATS guidelines	6/17/2015 4:01 PM
10	12% increase in FEV1 or FVC, which is 200 mL, in adults.	6/17/2015 2:37 PM
11	as guidelines	6/17/2015 10:54 AM
12	12	6/17/2015 9:43 AM
13	12%	6/16/2015 9:38 PM
14	15%	6/16/2015 9:00 PM
15	12%	6/16/2015 8:33 PM
16	12% OR MORE	6/16/2015 8:00 PM
17	12	6/16/2015 7:01 PM
18	12%	6/16/2015 5:43 PM
19	12	6/16/2015 3:37 PM
20	12 % and 200 ml over baswline	6/16/2015 2:42 PM
21	12% 200cc	6/16/2015 2:24 PM
22	12%	6/16/2015 2:00 PM
23	12	6/16/2015 1:36 PM
24	12% and	6/16/2015 1:22 PM
25	12 and 200	6/16/2015 1:01 PM
26	12% and 200 ml	6/16/2015 12:55 PM
27	not applicable	6/16/2015 12:42 PM
28	12%/200ml	6/16/2015 12:33 PM
29	FEV1 increase of 12% and 200mL	6/16/2015 11:36 AM
30	ATS criteria used	6/16/2015 11:33 AM
31	as above chosen	6/16/2015 11:33 AM

32	12% and 200 min FEV1l	6/16/2015 11:00 AM
33	12% and 400 ml	6/16/2015 10:14 AM
34	12% and 200 ml	6/16/2015 10:05 AM
35	see above	6/16/2015 9:56 AM
36	12% or 200 ml	6/16/2015 9:30 AM
37	12% and 200	6/16/2015 9:29 AM
38	12%	6/16/2015 9:23 AM
39	5-7% trend suggest that the diagnosis is correct. Then monitor with time of th improvement. Some patients are so severe that reversibility is noted more with steroids over weeks.	6/16/2015 9:15 AM
40	12 percent and 200 mL increase in FEV1	6/16/2015 9:14 AM
41	200ml	6/16/2015 9:12 AM
42	12%	6/16/2015 9:12 AM
43	12% or 200ml	6/16/2015 9:10 AM
44	15	6/16/2015 9:10 AM
45	10-15%	6/16/2015 9:09 AM
46	10% at keast	6/16/2015 9:09 AM
47	12% or 200ml	6/16/2015 9:05 AM
48	12% or 200ml	6/15/2015 8:48 PM
49	200ml	6/15/2015 8:44 PM
50	12% and 200 ml	6/14/2015 9:41 PM
51	12%, 200ml	6/14/2015 8:35 PM
52	12%, usually with improvement in FEF25-75 also.	6/14/2015 7:25 PM
53	per ATS criteria 12% increase in FEV1 and > 200 ml increase	6/14/2015 4:52 PM
54	12% and 200 ml	6/13/2015 8:39 PM
55	12 percent or 200 ml	6/12/2015 9:10 PM
56	12% and 200 ml	6/12/2015 12:27 PM
57	12-15%	6/12/2015 10:34 AM
58	as in #9 above	6/12/2015 7:51 AM
59	12	6/12/2015 6:19 AM
60	-	6/11/2015 11:34 AM
61	12% and 200mL	6/11/2015 9:23 AM
62	12% and 200 ml	6/10/2015 3:17 PM
63	12% and 200 ml	6/10/2015 2:53 PM
64	10-12%	6/10/2015 2:43 PM
65	12%	6/10/2015 2:14 PM
66	12% FEV1, 200 ml	6/10/2015 2:00 PM
67	200 mL and FEV1 >12%	6/10/2015 1:29 PM
68	I don't understand this question - see #9	6/10/2015 1:19 PM

69	12% and 200 mL	6/10/2015 1:13 PM
70	200 ml	6/10/2015 1:04 PM
71	12% or 200ml	6/10/2015 12:50 PM
72	12-15%	6/10/2015 11:32 AM
73	12% or greater	6/10/2015 11:26 AM
74	12% or 200 ml	6/10/2015 11:25 AM
75	12% and 200 ml	6/10/2015 10:26 AM
76	See question #9	6/10/2015 9:48 AM
77	12% or 200 ml	6/10/2015 9:14 AM
78	12	6/10/2015 9:06 AM
79	I go with a 12% increase, but I'm less inclined to look at absolute increase as a strict criterion, as I see everything from infants to advanced geriatrics, and normal predicted FEV1 varies significantly across my patient population.	6/10/2015 8:58 AM
80	12%	6/10/2015 8:37 AM
81	12% AND 200mL	6/10/2015 8:08 AM
82	12 or 200ml	6/10/2015 7:53 AM
83	12% and 200 mL; however, in many instances especially children, I view a significant increase in mid-expiratory flow rate (22% or more) as evidence of reversibility assuming good/consistent maneuver.	6/10/2015 6:16 AM
84	12% and 200mL	6/10/2015 1:54 AM
85	12% or 200ml	6/10/2015 12:39 AM
86	12% 200 ml	6/9/2015 10:57 PM
87	125 and at least 200 ml	6/9/2015 10:28 PM
88	At least 10% increase in FEV1	6/9/2015 9:56 PM
89	12 % increase FEV1	6/9/2015 8:46 PM
90	12%	6/9/2015 8:03 PM
91	>12% and 200mL	6/9/2015 7:51 PM
92	only use FEV1 15% and at least 200ml	6/9/2015 7:48 PM
93	12%	6/9/2015 7:48 PM
94	>121% FEV1 increase	6/9/2015 6:57 PM
95	12%	6/9/2015 6:55 PM
96	12% and 200ml	6/9/2015 6:27 PM
97	Since I deal with children, a 200ml increase in FEV1 may not be a relevant number. I use a 12% increase in FEV1 and a 20% increase in FEF 25-75% for diagnostic purposes. I do not use FVC by itself - a 12% increase may be considered only if accompanied by a concordant increase in FEV1.	6/9/2015 6:13 PM
98	12%	6/9/2015 6:00 PM
99	12% (15% even better) and 0.2 L	6/9/2015 5:23 PM
100	ATS guidelines as in question 9	6/9/2015 5:21 PM
101	% inc 12	6/9/2015 5:20 PM
102	12% 200 mL	6/9/2015 5:08 PM
103	12 % 200 ml	6/9/2015 5:03 PM
104	adult 12% and 200ml	6/9/2015 5:00 PM

105	FEVI increase of 12%	6/9/2015 4:53 PM
106	Increase in FEF 25-75 of 10 or more percent with good history	6/9/2015 4:47 PM
107	12% Range 10-20%	6/9/2015 4:24 PM
108	see above answer	6/9/2015 4:06 PM
109	12% and 200ml	6/9/2015 4:03 PM
110	FEV1 - 12% and 200ml	6/9/2015 3:56 PM
111	8% in kids 12% in adults	6/9/2015 3:44 PM
112	12% and 200ml in children and 12% or 200ml in adults	6/9/2015 3:41 PM
113	12% or 200ml	6/9/2015 3:38 PM
114	12% and 200cc	6/9/2015 3:32 PM
115	I would call 20% "POSITIVS" 12% "SIGNIFICANT" see above 12-20%: it depends on the story (pre-test probability)	6/9/2015 3:27 PM
116	8 % children , 12% adolescents and adults	6/9/2015 3:25 PM
117	12% or 200 mL	6/9/2015 3:16 PM
118	12% and atleast 200ml	6/9/2015 3:10 PM
119	12% and 200mL	6/9/2015 3:08 PM
120	12% and 200 mL	6/9/2015 3:01 PM
121	12% increase	6/9/2015 2:55 PM
122	15%	6/9/2015 2:50 PM
123	15	6/9/2015 2:49 PM
124	Increase in FEV1 of 12% and 200 ml over baseline	6/9/2015 2:43 PM
125	Pediatric population: 12% increase in FEV1	6/9/2015 2:39 PM
126	10%, 200mL	6/9/2015 2:32 PM
127	12% and 200mL FEV1	6/9/2015 2:31 PM
128	12%	6/9/2015 2:29 PM
129	12%	6/9/2015 2:25 PM
130	at least 12% and 200mL	6/9/2015 2:22 PM
131	12% and 200ml	6/9/2015 1:58 PM
132	15% or 200 ml	6/9/2015 1:58 PM
133	12	6/9/2015 1:48 PM
134	13% 200 ml	6/9/2015 1:44 PM
135	as per ATS guideline	6/9/2015 1:38 PM
136	DEPENDS ON CLINICAL SITUATION: 12% - 200 ML IN ADULTS IF HX IS NOT SUGGESTIVE OF BA; >8% IF PATIENT IS ALLERGIC AND /OR HX IS SUGGESTIVE	6/9/2015 1:27 PM
137	12% FEV1	6/9/2015 1:27 PM
138	see #9	6/9/2015 1:19 PM
139	see above ...12% and at least 200ml	6/9/2015 1:13 PM
140	12% and 200 ml in FEV1	6/9/2015 1:12 PM
141	12% and 200 ml	6/9/2015 1:11 PM

142	see ATS	6/9/2015 1:11 PM
143	200 ml	6/9/2015 1:11 PM
144	12 to 15% and 200 mls	6/9/2015 1:10 PM
145	As above	6/9/2015 1:07 PM
146	12% and 200 mL	6/9/2015 4:56 AM
147	12% 200 mL	6/9/2015 1:59 AM
148	12%, 200 ml	6/8/2015 8:34 PM
149	12%	6/8/2015 1:36 PM
150	200 mL and 12%	6/8/2015 12:10 PM
151	12% or 200 ml (low lung volume)	6/8/2015 11:34 AM
152	see above	6/8/2015 7:42 AM
153	12%	6/7/2015 9:04 PM
154	12% or 200cc.	6/7/2015 4:35 PM
155	An increase in 12% or 200 cc over baseline for either FEV1 and/or FVC	6/7/2015 3:19 PM
156	FEV1 12%	6/7/2015 2:48 PM
157	12% and 200ml	6/7/2015 1:33 PM
158	12	6/7/2015 12:01 PM
159	12%	6/6/2015 9:35 PM
160	> or equal to 15% FEV1	6/6/2015 12:32 PM
161	12%. I do not look at an absolute increase in ml.	6/6/2015 12:16 AM
162	12 and 200	6/5/2015 8:41 PM
163	about 15 percent	6/5/2015 6:30 PM
164	15%	6/5/2015 3:32 PM
165	12% or 200ml	6/5/2015 2:51 PM
166	12	6/5/2015 2:26 PM
167	12%	6/5/2015 1:51 PM
168	12%	6/5/2015 12:29 PM
169	12% 200 ml	6/5/2015 12:03 PM
170	for the pediatric patients I use 10 %	6/5/2015 10:34 AM
171	12% increase in FEV1	6/5/2015 4:40 AM
172	12%	6/4/2015 9:32 PM
173	12% abd 200 ml	6/4/2015 6:33 PM
174	12%	6/4/2015 5:46 PM
175	12 %	6/4/2015 4:30 PM
176	12% and 200 mls FEV1.	6/4/2015 4:28 PM
177	>=12%	6/4/2015 3:21 PM
178	12%, 200ml	6/4/2015 3:15 PM
179	>12% and 200 cc	6/4/2015 2:55 PM

180	12%	6/4/2015 1:17 PM
181	15%, 200ml	6/4/2015 11:59 AM
182	12% and 200 mL	6/4/2015 10:38 AM
183	12% and 200 ml, and also I look at peak flow improvement and FEF 25-75% improvement	6/4/2015 8:46 AM
184	12% 200 mL Less if child	6/4/2015 8:42 AM
185	20% change in FEV1	6/4/2015 8:01 AM
186	15%	6/4/2015 7:33 AM
187	12% increase and 200 ml in FEV1 or FVC over baseline	6/4/2015 7:17 AM
188	12%, 200 ml	6/4/2015 5:50 AM
189	12%/200ml	6/3/2015 9:37 PM
190	12%, 200 mL	6/3/2015 8:23 PM
191	Ats	6/3/2015 8:17 PM
192	12% and 200 mL	6/3/2015 7:56 PM
193	12	6/3/2015 7:35 PM
194	12%.200ml	6/3/2015 6:48 PM
195	12% and 200 ml	6/3/2015 5:09 PM
196	as above	6/3/2015 4:55 PM
197	12% 200 mL	6/3/2015 4:54 PM
198	12%	6/3/2015 4:42 PM
199	12% or above.	6/3/2015 4:37 PM
200	>12%rise in FEV1	6/3/2015 4:14 PM
201	10 or 12% depending on my mood that day	6/3/2015 3:06 PM
202	12% or 200 mL	6/3/2015 2:43 PM
203	15%	6/3/2015 2:16 PM
204	12% and 200 mL FEV1	6/3/2015 2:08 PM
205	12% and 200ml	6/3/2015 1:59 PM
206	12%, 200ml	6/3/2015 1:56 PM
207	12%	6/3/2015 1:10 PM
208	12%/200ml	6/3/2015 1:10 PM
209	15%	6/3/2015 1:06 PM
210	12%, 200 ml	6/3/2015 12:56 PM
211	12% and 200ml	6/3/2015 12:49 PM
212	as in # 9	6/3/2015 12:13 PM
213	see above	6/3/2015 11:27 AM
214	12	6/3/2015 11:17 AM
215	12% and 200 mL	6/3/2015 11:15 AM
216	12 and 200	6/3/2015 11:06 AM
217	ATS guidelines	6/3/2015 11:03 AM

218	15% and 200 ml	6/3/2015 10:50 AM
219	12 % increase in FEV1 or FVC AND at least 200 ml increase as well	6/3/2015 10:35 AM
220	12% 200ml	6/3/2015 10:34 AM
221	200	6/3/2015 10:26 AM
222	As above	6/3/2015 10:18 AM
223	12% IS NICE, BUT EVERYONE IGNORES FLOW AT MID-LUNG VOLUMES. Data from the Chernick lab showed a relationship with 7% chnage in FEV1 and AHR Almost any regular dose of ICS deminishes FEV1 reversibility	6/3/2015 10:17 AM
224	12% for FEV1	6/3/2015 10:11 AM
225	12% and at least 200 ml	6/3/2015 10:10 AM
226	12% and 200 ml	6/3/2015 10:08 AM
227	12% increase or/and 200ml	6/3/2015 9:59 AM
228	12%	6/3/2015 9:48 AM
229	12% and 200 ml	6/3/2015 9:31 AM
230	More than 12% and 200 ml	6/3/2015 9:28 AM
231	12% and 200 ml.	6/3/2015 9:21 AM
232	12% and 200 mL	6/3/2015 9:11 AM
233	12%	6/3/2015 8:43 AM
234	read all inc % but know the Standard 12%	6/3/2015 8:40 AM
235	12%	6/3/2015 8:38 AM
236	12-15% increase and 200 ml increase	6/3/2015 8:19 AM
237	12% and 200mL	6/3/2015 8:13 AM
238	12% and at least 200 ml	6/3/2015 8:11 AM
239	12% and 200 ml	6/3/2015 8:01 AM
240	12	6/3/2015 7:50 AM
241	10-12%	6/3/2015 7:45 AM
242	12% and 200ml	6/3/2015 7:33 AM
243	as above	6/3/2015 6:39 AM
244	12%	6/3/2015 6:31 AM
245	12% and 200mL	6/3/2015 5:49 AM
246	12%	6/3/2015 4:09 AM
247	12% or greater, and 200 ml	6/3/2015 3:47 AM
248	200ml	6/3/2015 2:45 AM
249	125	6/2/2015 11:11 PM
250	12% and 200 mL	6/2/2015 10:43 PM
251	at least 12 %	6/2/2015 10:37 PM
252	12%, 200 ml	6/2/2015 10:24 PM
253	12% or 200ml	6/2/2015 10:18 PM
254	15% increase from baseline.	6/2/2015 9:59 PM

255	12%, 200 ml	6/2/2015 9:59 PM
256	12% and 200 cc	6/2/2015 9:52 PM
257	n/a	6/2/2015 9:49 PM
258	12% and 200 ml	6/2/2015 9:39 PM
259	12%	6/2/2015 9:39 PM
260	12 and 200	6/2/2015 9:30 PM
261	12%	6/2/2015 9:26 PM
262	12 percent. 200 ml	6/2/2015 9:12 PM
263	12%	6/2/2015 9:03 PM
264	12% and 200 ml	6/2/2015 9:01 PM
265	> 12 % and 200ml over baseline for Fev1 or FVC.	6/2/2015 8:58 PM
266	12%	6/2/2015 8:51 PM
267	12%	6/2/2015 8:45 PM
268	200	6/2/2015 8:43 PM
269	200ml	6/2/2015 8:42 PM
270	13	6/2/2015 8:36 PM
271	12% 200ml	6/2/2015 8:19 PM
272	12% 200 ml	6/2/2015 8:19 PM
273	12 and 200cc	6/2/2015 8:07 PM
274	10 to 12 % begins to be significant 15% or greater to be positive	6/2/2015 8:06 PM
275	12%	6/2/2015 8:03 PM
276	12% and 200 ml	6/2/2015 7:59 PM
277	12 or 200	6/2/2015 7:57 PM
278	as above	6/2/2015 7:56 PM
279	12% 200	6/2/2015 7:47 PM
280	12	6/2/2015 7:42 PM
281	12% increase in FEV1	6/2/2015 7:31 PM
282	12	6/2/2015 7:25 PM
283	Not used.	6/2/2015 7:13 PM
284	12%	6/2/2015 7:10 PM
285	10-15% (not rigid)	6/2/2015 7:07 PM
286	12-15%	6/2/2015 7:04 PM
287	12 percent	6/2/2015 7:03 PM
288	FEV1 of 12% and 200ml	6/2/2015 7:03 PM
289	12-15%	6/2/2015 6:57 PM
290	FEV-1 improvement over baseline of 12%	6/2/2015 6:56 PM
291	12% change in FEV1, we usually don't measure the number of ml	6/2/2015 6:42 PM
292	8%, mls differ dependent on size, age, etc	6/2/2015 6:18 PM

293	12%	6/2/2015 6:13 PM
294	12% adn 200mL in an adult	6/2/2015 6:11 PM
295	12% and 200 ml	6/2/2015 6:10 PM
296	12% and 200 ml. of FEV1.	6/2/2015 6:03 PM
297	12%/200 ml	6/2/2015 5:54 PM
298	I except 12% improvement but would prefer to have 15% or more. I typically do not use a quantitative amount in milliliters for improvement.	6/2/2015 5:49 PM
299	10-12% and 200 ml	6/2/2015 5:47 PM
300	As above for FEV1	6/2/2015 5:47 PM
301	at least 12% improvement in FEV! and/or greater than 200 ml FVC?	6/2/2015 5:40 PM
302	12% 200ml	6/2/2015 5:38 PM
303	12 and 200	6/2/2015 5:36 PM
304	12	6/2/2015 5:36 PM
305	as above	6/2/2015 5:35 PM
306	12% and 200mL	6/2/2015 5:32 PM
307	12%	6/2/2015 5:31 PM
308	FEV-1 improvement by >=12% and at least 200 mL.	6/2/2015 5:30 PM
309	12%, 200 ml	6/2/2015 5:29 PM
310	12%, 200 ml	6/2/2015 5:22 PM
311	12% and 200ml in FEV1	6/2/2015 5:19 PM
312	12% AND 200 ml	6/2/2015 5:09 PM
313	12% or 200 ml	6/2/2015 5:09 PM
314	12	6/2/2015 5:08 PM
315	12% and 200ml	6/2/2015 5:06 PM
316	12%/200ml	6/2/2015 5:05 PM
317	12%, 200 ml	6/2/2015 5:00 PM
318	12 percent	6/2/2015 4:54 PM
319	12% and 200 ml	6/2/2015 4:54 PM
320	>12-15%	6/2/2015 4:49 PM
321	12-!5% increase tin FEV1	6/2/2015 4:46 PM
322	12%	6/2/2015 4:43 PM
323	12-15% and > 200 ml	6/2/2015 4:42 PM
324	12 % or 200ml	6/2/2015 4:37 PM
325	12, 200	6/2/2015 4:32 PM
326	10% may be sufficient depending on the case	6/2/2015 4:30 PM
327	12% and 200 ml inc. in FEV 1	6/2/2015 4:24 PM
328	12%	6/2/2015 4:23 PM
329	12% and at least 200 ml	6/2/2015 4:18 PM

330	I look for any reversibility	6/2/2015 4:16 PM
331	200 mL	6/2/2015 4:16 PM
332	12% and 200 ml	6/2/2015 4:15 PM
333	>12%	6/2/2015 4:09 PM
334	12%	6/2/2015 4:08 PM
335	12%, 200 ml	6/2/2015 4:08 PM
336	12 % for FEV1 but ml depends on age, if a child it may not be the 200 ml	6/2/2015 4:08 PM
337	12% increase or more	6/2/2015 4:08 PM
338	As above in ATS guidelines	6/2/2015 4:05 PM
339	12% and 200ml	6/2/2015 4:04 PM
340	Fev1 increase 15% and/or 40% increase FEF25-75%	6/2/2015 4:02 PM
341	12% and 200 ml	6/2/2015 4:01 PM
342	15	6/2/2015 4:00 PM
343	>12% rise in FEV1	6/2/2015 3:58 PM
344	12% and 200ml	6/2/2015 3:54 PM
345	12% and 200 ml	6/2/2015 3:54 PM
346	12%, 200mL.	6/2/2015 3:52 PM
347	12% and 200 ml in FEV1	6/2/2015 3:50 PM
348	12%/ 200mL	6/2/2015 3:50 PM
349	12% and 200 ml	6/2/2015 3:48 PM
350	An increase of 12% and 200 ml in FEV1 or FVC over the baseline	6/2/2015 3:46 PM
351	10%	6/2/2015 3:45 PM
352	12%, 200ml	6/2/2015 3:42 PM
353	as above	6/2/2015 3:41 PM
354	>12%	6/2/2015 3:41 PM
355	12% 200 ml	6/2/2015 3:40 PM
356	12 and 200	6/2/2015 3:40 PM
357	12%, 200 ml	6/2/2015 3:38 PM
358	10%	6/2/2015 3:37 PM
359	12%, 200mL	6/2/2015 3:37 PM
360	12% or more. Not sure we can use a ml change for children	6/2/2015 3:37 PM
361	12	6/2/2015 3:35 PM
362	12%	6/2/2015 3:35 PM
363	10%	6/2/2015 3:35 PM
364	12%/200mL	6/2/2015 3:34 PM
365	approx. 15%. Must be taken in context of individual patient	6/2/2015 3:32 PM
366	12 percent	6/2/2015 3:31 PM
367	12% and 200ml	6/2/2015 3:31 PM

368	12%	6/2/2015 3:30 PM
369	12% and 200 mL	6/2/2015 3:30 PM
370	12	6/2/2015 3:29 PM
371	ATS guidelines of 12% and 200cc for adults but for kids could be less	6/2/2015 3:29 PM
372	12, 200	6/2/2015 3:28 PM
373	15	6/2/2015 3:28 PM
374	see above	6/2/2015 3:27 PM
375	≥12% and 200 ml	6/2/2015 3:27 PM
376	15-20%	6/2/2015 3:26 PM
377	12% or 200 ml	6/2/2015 3:25 PM
378	12% increase in FEV1	6/2/2015 3:24 PM
379	12%	6/2/2015 3:23 PM
380	12%	6/2/2015 3:23 PM
381	12%	6/2/2015 3:23 PM
382	12 and 200	6/2/2015 3:23 PM
383	12% or 200 mL	6/2/2015 3:23 PM
384	12%	6/2/2015 3:22 PM

Q11 If a test is negative, but you still have a high index of suspicion, what do you do:

Answered: 452 Skipped: 44

#	Responses	Date
1	Check for change in FEF(25-75)	6/19/2015 4:48 PM
2	treat	6/19/2015 12:50 PM
3	treat anyway	6/19/2015 12:32 PM
4	Methacholine Also important that pts did not use Albuterol for 6 hours and LABA for at least 12 hours before spirometry	6/18/2015 5:24 PM
5	treatment trial	6/18/2015 4:49 PM
6	Either treat with a 7-10 day oral steroid burst and re-check PFT or give 6 weeks inhaled steroid and re-check PFT	6/18/2015 12:03 PM
7	Bronchoprovocational challenge (Methacholine)	6/18/2015 9:56 AM
8	treat empirically	6/17/2015 4:53 PM
9	Treat and reassess on next visit	6/17/2015 4:01 PM
10	Sometimes I do a FENO test before the reversibility test. If the FENO score is > 27, I lean towards asthma, if there is clinical suspicion.	6/17/2015 2:37 PM
11	treat	6/17/2015 10:54 AM
12	therapeutic trial of albuterol and or prednisone	6/17/2015 9:43 AM
13	Feno	6/16/2015 9:38 PM
14	Consider to refer for cold air challenge, or exercise challenge	6/16/2015 9:00 PM
15	look at FEF 25- 75 and listen to the chest	6/16/2015 8:33 PM
16	TREAT ANYWAY ON PATIENT HISTORY AND PHYSICAL EXAM....YOU LOOK AT THE PATIENT NOT THE TEST.....AND ON FOLLOW UP....THEIR LUNG FUNCTION IS MUCH IMPROVED ON MAINTENANCE MEDICATION...MORE THAN 12% ABOVE THE BASELINE OF THE PRIOR VISIT	6/16/2015 8:00 PM
17	Repeat if symptoms are present	6/16/2015 7:01 PM
18	Methacholine Trial of prednisone Trial of albuterol with peak flow monitoring	6/16/2015 6:00 PM
19	methacholine challenge- done at outside facility	6/16/2015 5:43 PM
20	Begin a therapeutic trial with an inhaled corticosteroid.	6/16/2015 4:09 PM
21	Follow patient clinically. Treat based on symptoms. If not improving consider other diagnosis. Alternatively, if already on medicatons consider stopping all meds and retest OR perform a MCT	6/16/2015 3:37 PM
22	Petty protocol with oral steroid	6/16/2015 2:42 PM
23	Higher dose or corticosteroid burst.	6/16/2015 2:24 PM
24	Trial on Albuterol at home	6/16/2015 2:10 PM
25	Send patient for PFT	6/16/2015 2:00 PM
26	Methacholine	6/16/2015 1:36 PM
27	tret empirically with ICS	6/16/2015 1:22 PM
28	Treat	6/16/2015 1:01 PM
29	methacholine challenge or an exercise challenge	6/16/2015 12:55 PM

30	treat with controller medications to see if symptoms improve	6/16/2015 12:42 PM
31	probably treat as asthma until proven otherwise	6/16/2015 12:33 PM
32	If smaller% list it as minimal reversibility or none	6/16/2015 12:22 PM
33	If needed consider challenge with cold air, exercise or McH	6/16/2015 12:12 PM
34	trial of controller therapy and follow-up to monitor response	6/16/2015 11:55 AM
35	Treat with bronchodilators, send for methacholine challenge	6/16/2015 11:36 AM
36	Repeat on a different day or order methacholine challenge	6/16/2015 11:33 AM
37	send for metacholine	6/16/2015 11:33 AM
38	Methacholine or mannitol challenge	6/16/2015 11:18 AM
39	Do methacholeine challenge	6/16/2015 11:00 AM
40	repeat the test making sure that SABA use has been restricted > 4 hours and LABA > 12 hours	6/16/2015 10:14 AM
41	order formal pulmonary function testing with methacholine challenge	6/16/2015 10:12 AM
42	repeat after a few weeks. check for medication response at f/u.	6/16/2015 10:05 AM
43	FeNO, repeat spirometry, perhaps home PEF measurement	6/16/2015 9:56 AM
44	Treat with asthma controller and follow up in 2-6 weeks	6/16/2015 9:30 AM
45	Sometimes give 2 additional puffs, but sometimes still assume asthma if history is strong enough. If negative test and history concerning but not suggestive of asthma, may consider full pulmonary function testing.	6/16/2015 9:29 AM
46	Consider FeNo or send for DCLO	6/16/2015 9:23 AM
47	Exercise	6/16/2015 9:15 AM
48	Usually, methacholine challenge	6/16/2015 9:14 AM
49	may treat empirically first. If not helpful may consider mannitol challenge.	6/16/2015 9:12 AM
50	Possibly trial of medications	6/16/2015 9:12 AM
51	Methacholine challenge	6/16/2015 9:10 AM
52	FeNO, methacholine challenge, therapeutic trial of albuterol	6/16/2015 9:10 AM
53	often order a methacholine challenge	6/16/2015 9:09 AM
54	Treat as asthma with at home beta agonist and obtain peak flow record for 1 month	6/16/2015 9:09 AM
55	look at PEF changes and sometimes administer second dose of albuterol.	6/16/2015 9:06 AM
56	Place patient on a bronchodilator	6/16/2015 9:05 AM
57	Burst with steroids x 5 days to induce receptor number. Repeat testing after the 5 days.	6/15/2015 8:48 PM
58	methacholine challenge	6/15/2015 8:44 PM
59	Clinical trial of inhaled corticosteroid or oral corticosteroid and return visit for reversibility assessment	6/14/2015 9:41 PM
60	methacholine challenge,excercise challenege	6/14/2015 8:35 PM
61	(Assuming the patient had NOT used bronchodilator including LABA at home) tell the patient the test is normal/negative, but the history does suggest asthma. Likely repeat the test with illness and/or in their allergy "season" for 1 with significant allergic disease.	6/14/2015 7:25 PM
62	either methacholine challenge test or trial of presumptive treatment	6/14/2015 4:52 PM
63	Methacholine challenge	6/14/2015 1:43 AM
64	Methacholine test	6/13/2015 8:39 PM
65	Treat as if asthma is present	6/12/2015 9:10 PM

66	a clinical trial of a bronchodilator for 1-2 weeks	6/12/2015 12:27 PM
67	Treat with ICS and SABA and monitor response	6/12/2015 10:34 AM
68	exercise or methacholine challenge	6/12/2015 9:05 AM
69	I will institute a trial of systemic corticosteroids for two weeks, and re test after that	6/12/2015 7:51 AM
70	Methacholine challenge	6/12/2015 6:19 AM
71	Mech cahllenge	6/11/2015 11:34 AM
72	Do not exclude a diagnosis of asthma. Need to assess recent SABA and LABA use. I find some atopic pts will only show reversibility in setting of allergen exposure.	6/11/2015 9:23 AM
73	treat with combination for > 3 week retest	6/11/2015 12:00 AM
74	trial with albuterol or singulair, follow up in a few months	6/10/2015 3:17 PM
75	Treat the patient as I would for asthma.	6/10/2015 2:53 PM
76	Repeat or consider methacholine challange.	6/10/2015 2:43 PM
77	therapeutic trial	6/10/2015 2:19 PM
78	methacholine challenge	6/10/2015 2:14 PM
79	Treat symptomatically; perhaps pursue methacholine challenge. Patient may already be on ICS, interfering with challenge itself.	6/10/2015 2:00 PM
80	methacholine challenge	6/10/2015 1:29 PM
81	monitor clinically, possibly methacholine challenge	6/10/2015 1:19 PM
82	treat	6/10/2015 1:13 PM
83	repeat at next visist	6/10/2015 1:04 PM
84	Usually treat if asthma is suspected and have the patient follow up after a month to assess for improvement.	6/10/2015 12:50 PM
85	may treat empirically	6/10/2015 11:58 AM
86	try oral or ICS and re- test in 2-4 weeks	6/10/2015 11:41 AM
87	FeNO and consider methacholine challenge	6/10/2015 11:32 AM
88	Methacholine challenge	6/10/2015 11:26 AM
89	Review the response in the midflow (FEF 25-75)	6/10/2015 11:25 AM
90	treat as asthma anyway	6/10/2015 10:26 AM
91	Have	6/10/2015 9:48 AM
92	Trial of ICS for 1 month and repeat PFT	6/10/2015 9:14 AM
93	Therapeutic medication trial	6/10/2015 9:06 AM
94	Follow peak flows and do a trial of inhaled or oral corticosteroids, depending upon the situation, then re-test.	6/10/2015 8:58 AM
95	Treat with oral steroids and remeasure after a course.	6/10/2015 8:37 AM
96	Empiric therapy or METH testing	6/10/2015 8:08 AM
97	Get full PFTS or treat	6/10/2015 7:53 AM
98	I may get FeNO--though this is a marker of eosinophilic airways inflammation and there are certainly patents without elevated FeNO who have asthma. I will typically given them a trial of short-acting bronchodilator to use when symptomatic and monitor response to treatment or depending on frequency of symptoms, a trial of controller medication (ICS or ICS/LABA) and then return for follow-up evalution and repeat spirometry.	6/10/2015 6:16 AM
99	treat for asthma anyway	6/10/2015 6:04 AM
100	Trial of an inh steroid, return for repeat testing	6/10/2015 5:55 AM

101	Bronchoprovocation study	6/10/2015 1:54 AM
102	Will perform a methacholine provocation testr	6/10/2015 12:39 AM
103	Niox Med trial Methochcoline challenge	6/9/2015 10:57 PM
104	A methacholine challenge. Use to do Mannitol Challenge, but the solution is in back order now for many month	6/9/2015 10:28 PM
105	Trial of inhaled corticosteroids for 2 weeks and see if respiratory symptoms improve	6/9/2015 9:56 PM
106	I often have already performed FeNO on pt. prior to doing spirometry. I sometimes order methacholine challenges, but not often. I don't do methacholine challenge in my office.	6/9/2015 9:26 PM
107	Repeat on the followup	6/9/2015 8:46 PM
108	Methacholine challenge	6/9/2015 8:03 PM
109	Continue to treat as planned	6/9/2015 7:51 PM
110	give additional 2 puff of MDI	6/9/2015 7:48 PM
111	methacholine challenge	6/9/2015 7:48 PM
112	TRIAL OF ASTHMA MEDICATION INHALER OR ORAL STEROIDS	6/9/2015 7:17 PM
113	Repeat in the future, consider 7 - 10 day course of prednisone	6/9/2015 7:10 PM
114	Consider methacholine, or repeat at a future time.	6/9/2015 6:57 PM
115	methacholine challenge	6/9/2015 6:55 PM
116	trial of systemic steroids	6/9/2015 6:27 PM
117	A course of steroids. We all know that if a patient has mostly inflammation and minimal bronchospasm, bronchodilators will have a minimal effect on PFT numbers.	6/9/2015 6:13 PM
118	Treat accordingly. On occasion order methacholine challenge	6/9/2015 6:00 PM
119	First determine whether mitigating factors are present that would improve lung function sufficiently to render same-day reversibility inapparent.	6/9/2015 5:23 PM
120	bronchoprovocation with methacholine or controller treatment initiation depending on the clinical circumstances.	6/9/2015 5:21 PM
121	consider other diagnosis	6/9/2015 5:20 PM
122	Trial of inhaled corticosteroid for one month and then reevaluate.	6/9/2015 5:08 PM
123	combination LABA and ICS for a 2-4 weeks and look for improvement	6/9/2015 5:03 PM
124	treat	6/9/2015 5:00 PM
125	Treat for asthma and repeat spirometry next visit	6/9/2015 4:55 PM
126	Repeat the test at the next visit	6/9/2015 4:53 PM
127	Trial of treatment and repeat 3 weeks later	6/9/2015 4:47 PM
128	exercise test or methacholine challenge	6/9/2015 4:41 PM
129	often treat as asthma	6/9/2015 4:24 PM
130	consider empiric treatment consider mannitol or methacholine challenge	6/9/2015 4:06 PM
131	FeNO, empiric treatment	6/9/2015 4:03 PM
132	methacholine challenge	6/9/2015 3:56 PM
133	Trial of medication Methacholine challenge (less often used in kids)	6/9/2015 3:44 PM
134	Try on medications and see back soon Methacholine Exericise challenge as appropriate	6/9/2015 3:41 PM
135	I may refer for metacholine testing	6/9/2015 3:38 PM
136	empiric trial of ICS therapy	6/9/2015 3:32 PM

137	If I think they have asthma I would trial treatment If I think they do not have asthma I would obtain a methacholine test	6/9/2015 3:27 PM
138	Give albuterol nebulization or 2 more puffs albuterol MDI	6/9/2015 3:25 PM
139	If test and FeNO are completely negative/normal, patient needs other tests for dyspnea or cough (depending on the main complaint) which could include a exercise treadmill test for coronary insufficiency and or evaluation for acid reflux and chest x-ray	6/9/2015 3:16 PM
140	methacholine challenge	6/9/2015 3:10 PM
141	Depends on the clinical scenario: possibly PRN SABA, possibly ICS monotherapy, possibly ICS/LABA.	6/9/2015 3:08 PM
142	Treat	6/9/2015 3:01 PM
143	Clinical correlation	6/9/2015 2:55 PM
144	Clinical trial to see if there is subjective improvement	6/9/2015 2:50 PM
145	use anti inflamatory therapy	6/9/2015 2:49 PM
146	exercise challenge, methacholine chalelnge, or trial of therapy	6/9/2015 2:43 PM
147	Arrange methacholine challenge	6/9/2015 2:39 PM
148	Methacholine test	6/9/2015 2:32 PM
149	Therapeutic trial of inhaled steroid and see them back	6/9/2015 2:31 PM
150	IOS	6/9/2015 2:29 PM
151	meth chall	6/9/2015 2:29 PM
152	Treat with inhaled steroid	6/9/2015 2:25 PM
153	If clinical suspicion is still very high for asthma or the reversibility is close to 12%, then I will often consider a trial of asthma therapy.	6/9/2015 2:22 PM
154	Trial of asthma therapy and re-assess patient in 4 weeks	6/9/2015 1:58 PM
155	Full PFT's, repeat in >6 weeks	6/9/2015 1:58 PM
156	treat as asthma	6/9/2015 1:48 PM
157	Therapeutic trial	6/9/2015 1:44 PM
158	Methacholine challenge	6/9/2015 1:40 PM
159	a trial of pred. and inhaled steroid	6/9/2015 1:38 PM
160	Treat empirically with ICS for 2 months and repeat testing as well as assess for symptom improvement	6/9/2015 1:29 PM
161	METH CHALL TEST	6/9/2015 1:27 PM
162	complete PFTs and methacholine challenge	6/9/2015 1:27 PM
163	provocation challenge	6/9/2015 1:19 PM
164	consider methacholine challenge	6/9/2015 1:13 PM
165	Note result does not meet criteria and comment in EHR that my overall impression is still that pt has asthma based on other data.	6/9/2015 1:12 PM
166	methacholine or mannitol challenge	6/9/2015 1:11 PM
167	Variable, but will most likely move forward with presumptive treatment.	6/9/2015 1:11 PM
168	Check FeNO or treat emperically	6/9/2015 1:10 PM
169	Start low dose ICS or go on to Methacholine Inhalation challenge, depending on discussion with patient.	6/9/2015 1:07 PM
170	Med trial	6/9/2015 1:07 PM
171	treat anyway and assess clinical response to bronchodilator	6/9/2015 7:41 AM

172	Trial of therapy followed by repeat PFT or methacholine challenge	6/9/2015 7:24 AM
173	treat anyway	6/9/2015 4:56 AM
174	Trial of treatment	6/9/2015 1:59 AM
175	Consider inhalation technique, amount of albuterol received, time, age of patient, more albuterol or Xopenex	6/8/2015 8:34 PM
176	go ahead and treat	6/8/2015 1:36 PM
177	treat empirically and assess response OR give prednisone, repeat spirometry after pred burst	6/8/2015 12:10 PM
178	Treat or5 methacholine challenge	6/8/2015 11:34 AM
179	send for exercise or methacholine challenge, or just treat	6/8/2015 7:42 AM
180	Treat the patient as if the test was positive and review at follow up in 3 to 4 weeks	6/7/2015 9:04 PM
181	Order methacholine challenge with full PFT's.	6/7/2015 4:35 PM
182	Still treat the patient as if they have asthma	6/7/2015 3:19 PM
183	treat and reassess	6/7/2015 2:48 PM
184	Methacholine or mannitol bronchoprovocation test	6/7/2015 2:37 PM
185	Treat patient for asthma.	6/7/2015 1:33 PM
186	Treat empiricly	6/7/2015 12:01 PM
187	Therapeutic challenge: A 3-4 week trial of an ICS or ICS/LABA to determine whether there is improvement in symptoms and/or spirometry.	6/6/2015 9:35 PM
188	peak flow meter given to patient with instructions to log during symptoms	6/6/2015 12:32 PM
189	FENO. A trial of Albuterol nevertheless. Methacholine challenge. Why did Mannitol go off the market?	6/6/2015 12:16 AM
190	trial of medication	6/5/2015 8:41 PM
191	Methacholine challenge.	6/5/2015 3:32 PM
192	Empiric asthma treatment	6/5/2015 2:51 PM
193	methacholine challenge	6/5/2015 2:26 PM
194	24% increase in FEF25-75%, or check FeNO	6/5/2015 1:51 PM
195	Therapeutic trial	6/5/2015 12:29 PM
196	bronchoprovacation	6/5/2015 12:03 PM
197	IOS	6/5/2015 10:34 AM
198	Send the patient for full PFTs lung volumes etc and methacholine challenge test.	6/5/2015 4:40 AM
199	Start an ICS.	6/4/2015 9:32 PM
200	Trial of ICS x 2-4 weeks and RTC for repeat spirometry. Occasoinally, I will give a trial of oral corticosteroids for 5-7 days and have them come back for spirometry	6/4/2015 6:33 PM
201	Check response to therapy for 4 to 6 weeks	6/4/2015 5:46 PM
202	treat the pt empirically	6/4/2015 4:30 PM
203	Treat accordingly.	6/4/2015 4:28 PM
204	Look at the FeNO, consider broncho-provocation such as methacholine	6/4/2015 3:21 PM
205	emperically treat for asthma	6/4/2015 3:15 PM
206	methacholine challenge is indicated, but I do not conduct it	6/4/2015 2:55 PM
207	Give prednisone and bring back for PFT after oral prednisone.	6/4/2015 1:17 PM
208	Treat but usually not with LABA	6/4/2015 1:17 PM

209	treat empirically with follow up and re-evaluation in 6-8 weeks	6/4/2015 12:40 PM
210	methacholine challenge	6/4/2015 11:59 AM
211	depends...sometimes methacholine testing	6/4/2015 11:40 AM
212	look at FEF 25-75	6/4/2015 10:38 AM
213	empiric trial of medications/steroids	6/4/2015 8:46 AM
214	Rx and repeat baseline on return	6/4/2015 8:42 AM
215	Methacholine or check peak flows at home	6/4/2015 8:01 AM
216	Place the patient on bronchodilator and or ICS therapy	6/4/2015 7:34 AM
217	methacholine challenge	6/4/2015 7:33 AM
218	Formal PFTs with bronchodilator challenge, or if patient is highly symptomatic empiric treatment with asthma medications	6/4/2015 7:17 AM
219	Treat and recheck in 2-4 weeks	6/4/2015 7:15 AM
220	Methacholine on another day	6/4/2015 5:50 AM
221	Niox and/or trial of treatment	6/3/2015 10:11 PM
222	eniox	6/3/2015 9:43 PM
223	Consider an NiOx test. Also, if the pt. reports relief of symptoms or wheezing resolves, a therapeutic trial of controller medication , or an increase in controller medication may be considered. If there is reversible coving of the flow-volume loop it may also affect my decision.	6/3/2015 9:37 PM
224	empirically treat and reassess spirometry after a few weeks of treatment; also assess symptom control after treatment	6/3/2015 8:23 PM
225	Repeat in 1-2 weeks	6/3/2015 8:17 PM
226	peak flow meter; repeat test later	6/3/2015 7:56 PM
227	treat like asthma and redo PFT in 3 months	6/3/2015 7:45 PM
228	trial of ics and repeat ACT and Spirometry	6/3/2015 7:35 PM
229	treat empirically	6/3/2015 6:48 PM
230	Still treat according to my suspicion	6/3/2015 5:09 PM
231	treat as asthma and repeat test in 2 -3 weeks	6/3/2015 4:55 PM
232	Treat with inhaled or oral corticosteroids	6/3/2015 4:54 PM
233	start meds, ICS, then recheck FEV1 in 1 month	6/3/2015 4:42 PM
234	Will treat patient and have this repeated in 3-4 weeks	6/3/2015 4:37 PM
235	Trial of medication.	6/3/2015 4:14 PM
236	Treat or consider a methacholine challenge.	6/3/2015 3:37 PM
237	consider retest at a later date to make sure they did not use medication prior to testing and not inform us.	6/3/2015 3:33 PM
238	have the patient try albuterol at home in 'real life' situations	6/3/2015 3:06 PM
239	Oral steroids for 5-7 days	6/3/2015 2:43 PM
240	repeat after a course of anti-inflammatory drug therapy	6/3/2015 2:16 PM
241	rely on clinical picture for decision making	6/3/2015 2:08 PM
242	Trial of medications and have the patient follow up	6/3/2015 1:59 PM
243	Treat as if asthma present	6/3/2015 1:56 PM
244	may treat empirically	6/3/2015 1:10 PM

245	Clinical trial of asthma therapy and follow-up spirometry.	6/3/2015 1:10 PM
246	10 day course of oral steroid (rarely needed)	6/3/2015 1:06 PM
247	Treat as asthma, re-evaluate in 3-4 weeks.	6/3/2015 12:56 PM
248	still treat for asthma as I would anyway	6/3/2015 12:49 PM
249	eNO	6/3/2015 12:13 PM
250	Methacholine challenge	6/3/2015 11:46 AM
251	Treat with low dose inhaled steroid and follow up in six weeks to assess symptom control with repeat lung function testing.	6/3/2015 11:45 AM
252	consider therapeutic trial on anti-inflammatory therapy	6/3/2015 11:27 AM
253	Treat or repeat test without LABA if on.	6/3/2015 11:15 AM
254	methacholine challenge on a separate visit	6/3/2015 11:06 AM
255	Treat empirically with inhaled steroids, then follow up to judge clinical response.	6/3/2015 11:03 AM
256	treat, repeat study in 6-12 months	6/3/2015 10:50 AM
257	place patient on a diagnostic and therapeutic trial of systemic or inhaled steroid and bring them back and repeat the spirometry testing--sometimes it is the reversibility from one visit to another that helps support the diagnosis, not always a reversibility on the same visit	6/3/2015 10:35 AM
258	Start medication; repeat PFTs when see back.	6/3/2015 10:34 AM
259	Repeat at next visit. See how they do with therapeutic treatment	6/3/2015 10:26 AM
260	still use bronchodilator and inhale corticosteroid trial for 2 weeks	6/3/2015 10:21 AM
261	If numbers suggest small airway disease/copd I give a course of oral steroids to assess benefit - have had occasional pts unresponsive to albuterol have fev1 and fef25-75 improve up to 50% with steroid.	6/3/2015 10:18 AM
262	Trial of therapy Consider MCh Challenge but with awareness of Liem et al and Carlson et al	6/3/2015 10:17 AM
263	Treat the patient's clinical symptoms. Evaluate for other possible causes of symptoms.	6/3/2015 10:11 AM
264	Trial of inhaled steroid/laba	6/3/2015 10:10 AM
265	Consider methacholine challenge	6/3/2015 10:08 AM
266	Try treatment with Ipratropium and repaet PFT	6/3/2015 9:59 AM
267	treat patient as if they had demonstrable reversibility	6/3/2015 9:48 AM
268	Therapeutic trial of medication	6/3/2015 9:31 AM
269	Methacholine challenge test	6/3/2015 9:28 AM
270	Oral steroids for 10 days and repeat PFT's or send to pulmonary for methacholine challenge.	6/3/2015 9:21 AM
271	Treat based on symptoms- spirometry is just a piece of the puzzle	6/3/2015 9:11 AM
272	Look at FEF 25-75 reversibility	6/3/2015 8:43 AM
273	Test again later, plus eNO, plus Pt measures PF at home...	6/3/2015 8:40 AM
274	May give a course of oral steroids and see the patient back in follow up	6/3/2015 8:38 AM
275	Discuss this with patient, instruct them to use albuterol as needed, and schedule follow up	6/3/2015 8:13 AM
276	give a second dose, usully albuterol + atrovent	6/3/2015 8:11 AM
277	Pulmonary function testing instead of just spirometry	6/3/2015 8:10 AM
278	treat for asthma and reassess in 1-3 months	6/3/2015 8:09 AM
279	Follow clinically or do methacholine challenge	6/3/2015 8:01 AM
280	Methacholine challenge	6/3/2015 7:53 AM

281	Repeat at next visit	6/3/2015 7:50 AM
282	Yes	6/3/2015 7:45 AM
283	Repeat test at next visit or send for formal pulmonary function testing	6/3/2015 7:33 AM
284	Trial of Oral steroids for 2 weeks then repeat testing if Im looking at posible COPD or severe obstruction, sometimes in mild cases I will take them off ICS and repeat in 3-4 weeks	6/3/2015 6:39 AM
285	a. Nothing since that's not how asthma is diagnosed. b. Prednisone for a week and repeat c. Methacholine. d. Something else.	6/3/2015 6:31 AM
286	still treat for asthma	6/3/2015 6:23 AM
287	methacholine	6/3/2015 6:05 AM
288	continue to use bronchodilator	6/3/2015 5:49 AM
289	check NIOX and treat empirically with followup both	6/3/2015 4:09 AM
290	Trial ICS treatment and repeat tests in 4 weeks	6/3/2015 3:47 AM
291	Treat	6/3/2015 2:45 AM
292	methacholine challenge	6/3/2015 1:04 AM
293	Treat with ICS/LABA BID for 1 month and have them return for spirometry or treat with course of oral steroid and SABA and have return for spirometry in 2 weeks	6/2/2015 11:59 PM
294	Look at change in FEF25-75 also	6/2/2015 11:11 PM
295	Still manage clinically as asthma	6/2/2015 10:51 PM
296	Treat the pt with appropriate med regimen and follow peak flows as an outpt; plan repeat spiro at follow up visit	6/2/2015 10:43 PM
297	put the patient on steroids for a week and have them return for followup spiromtery	6/2/2015 10:37 PM
298	Empirical therapy; repeat spiro testing at another time; consider methacholine challenge	6/2/2015 10:24 PM
299	Check FeNO, perform a Provocholine challenge	6/2/2015 10:18 PM
300	Trial of LABA+ICS for 2-4 weeks	6/2/2015 9:59 PM
301	Treat with a controller for a month and reassess	6/2/2015 9:59 PM
302	methacholine	6/2/2015 9:52 PM
303	trial of ICS	6/2/2015 9:49 PM
304	repeat after 15 more minutes	6/2/2015 9:39 PM
305	Trial of inhaled corticosteroid	6/2/2015 9:39 PM
306	use asthma meds on a trial basis if there is a 5-11% iimprovement in FEV1 and/or dramatic increase in fef- 25-75 and subjective improvement, I will treat this as if it were an positive response and treat accordingly.	6/2/2015 9:30 PM
307	repeat	6/2/2015 9:26 PM
308	Empirically treat for asthma	6/2/2015 9:20 PM
309	trial 10d po cs or 3wks high dose inhaled cs if gerd rx	6/2/2015 9:16 PM
310	Treat patient	6/2/2015 9:12 PM
311	Challenge	6/2/2015 9:04 PM
312	Manitol or methacholine challenge	6/2/2015 9:03 PM
313	Give 40 to 60 mg of prednisone for 6 -7 days and retest	6/2/2015 9:01 PM
314	Either an oral prednisone for 1 week then repeat or 2-4 weeks of an ICS and repeat. If still negative with high index of suspicion then Methicholine challenge.	6/2/2015 8:58 PM
315	trial medication	6/2/2015 8:51 PM

316	Send for body box PFTs to evaluate lung volumes pre and post bronchodilator for isovolume shift.	6/2/2015 8:45 PM
317	Methacholine challenge	6/2/2015 8:43 PM
318	Consider methacholine	6/2/2015 8:42 PM
319	Course of po steroids with follow up spirometry	6/2/2015 8:38 PM
320	Still treat according to clinical suspicion	6/2/2015 8:36 PM
321	treat c albuterol inhaler	6/2/2015 8:19 PM
322	Trial of inhaled steroid	6/2/2015 8:19 PM
323	Methacholine challenge	6/2/2015 8:07 PM
324	Treat empirically	6/2/2015 8:07 PM
325	Depends, I may order an exercise challenge. I used to do Aridol	6/2/2015 8:06 PM
326	methacholine challenge	6/2/2015 8:03 PM
327	Either exhaled nitric oxide or methacholine challenge	6/2/2015 7:59 PM
328	Treat emperically. If your doing a reversibility test, there fev1 is probably under 80% to begin with	6/2/2015 7:57 PM
329	stratify mild or moderate BD response	6/2/2015 7:56 PM
330	continue work up and treatment	6/2/2015 7:47 PM
331	FENO or methacholine	6/2/2015 7:43 PM
332	Methacholine	6/2/2015 7:42 PM
333	Corticosteroid trial and PFT	6/2/2015 7:31 PM
334	PFT AND THEN METACHOLINE CHALLENGE	6/2/2015 7:25 PM
335	1. Give asthma treatment trial either with 7 day course of oral steroids, or with daily controller medications to see if that would help symptoms and lung function.	6/2/2015 7:13 PM
336	depends on patient- trial of inhaled steroids	6/2/2015 7:10 PM
337	Put patient on combination ICS and LABA and repeat PFT in 2-4 weeks	6/2/2015 7:07 PM
338	Repeat if FEV1 declines throughout visits.	6/2/2015 7:07 PM
339	possibly treat and have the patient return or repeat the breathing test days or weeks later.	6/2/2015 7:04 PM
340	Trial of Ics order full pfts	6/2/2015 7:03 PM
341	methacholine challenge in most cases	6/2/2015 7:03 PM
342	give a trial of bronchodilators in real world settings and reevaluate in one month	6/2/2015 6:57 PM
343	Repeat when symptomatic or repeat with field exercise challenge	6/2/2015 6:56 PM
344	Methacholine challenge. Medications on trial	6/2/2015 6:42 PM
345	Treat the patient depending on the history. We see lots of individuals with normal lung function or no significant reversibility who have EIB.	6/2/2015 6:42 PM
346	Treat	6/2/2015 6:30 PM
347	Bronchochallenge	6/2/2015 6:22 PM
348	Start LABA/INS and repeat PFTs in 1 week	6/2/2015 6:18 PM
349	For new patients I give them a medium dose inhaled steroid BID for one month then repeat spirometry as the problem is likely more inflammation than spasm	6/2/2015 6:14 PM
350	methacholine challenge	6/2/2015 6:13 PM
351	If abnormal but not reversible, PO Steroids and repeat testing. If normal consider exercise challenge or methacholine.	6/2/2015 6:11 PM

352	Diagnose asthma using a detailed H & P.	6/2/2015 6:10 PM
353	Treat them anyway.	6/2/2015 6:03 PM
354	Methacholine or mannitol challenge, FENO	6/2/2015 5:54 PM
355	use bronchodilator therapy anyway. The patient with years or many months of airflow obstruction may not show significant reversibility in 15-20 minutes, but may after extended therapy showed overall improvement in spirometry.	6/2/2015 5:49 PM
356	trial of treatment	6/2/2015 5:47 PM
357	Increase number of puffs to assess reversibility. Would go to maximum bronchodilator effect. This is usually done with a research protocol since most of my work wis with asthma research.	6/2/2015 5:47 PM
358	Trial of corticosteroids in some situations, repeat test in 3-6 weeks; or give trial of pen albuterol.	6/2/2015 5:40 PM
359	Initiate treatment follow up in 6-8 weeks	6/2/2015 5:38 PM
360	Methacholine.	6/2/2015 5:36 PM
361	depends on patient. Consider full PFTs in pulm lab or treat for asthma with appropriate medications or consider prescribing albuterol and then keeping a daily symptom diary and have patient return in 2-3 weeks for re-evaluation with repeating office spiromety	6/2/2015 5:36 PM
362	repeat the testing at other time	6/2/2015 5:35 PM
363	therapeutic trial, consider OCS burst then repeat PFTs	6/2/2015 5:32 PM
364	treat the patient clinically with ICS and SABA	6/2/2015 5:31 PM
365	Methacholine challenge	6/2/2015 5:30 PM
366	Give pt bronchodilator to try when sx present. Sometimes if sx are chronic and daily, I give daily ICS and prn bronchodilator, then follow peak flows and repeat baseline spirometry at later date.	6/2/2015 5:29 PM
367	Methacholine challenge or trial of ICS	6/2/2015 5:22 PM
368	Trial of inhaled steroid	6/2/2015 5:19 PM
369	repeat test at later date	6/2/2015 5:09 PM
370	Still treat for asthma	6/2/2015 5:09 PM
371	Methacholine challenge test	6/2/2015 5:09 PM
372	Begin ICS	6/2/2015 5:08 PM
373	Send for PFTs or methacholine	6/2/2015 5:06 PM
374	clinical trials of meds	6/2/2015 5:05 PM
375	consider trial of ICS/LABA, consider methacholine challenge	6/2/2015 5:00 PM
376	i use prednisone systemic for a few days and retest.	6/2/2015 4:54 PM
377	consider empiric treatment	6/2/2015 4:49 PM
378	monitor peak flows at home over time, with pre and post bronchodilator response at home	6/2/2015 4:46 PM
379	Check a FeNO, treat empericly or if needed get a methacholine challenge	6/2/2015 4:43 PM
380	Occasionally the FEV1 decreases, in which case I assume an arg-arg phenotype and prescribe ipratopium with good success. Rarely send patient for methacholine challenge.	6/2/2015 4:42 PM
381	medication trial.	6/2/2015 4:37 PM
382	repeat next visit	6/2/2015 4:32 PM
383	I treat the patient...	6/2/2015 4:30 PM
384	Medication trial	6/2/2015 4:24 PM
385	treat and recheck for response	6/2/2015 4:23 PM

386	Treat empirically, or if really questionable, a methacholine challenge test.	6/2/2015 4:18 PM
387	administer steroids either inhaled or oral and repeat exam in 1-3 weeks	6/2/2015 4:17 PM
388	treat as asthma until proven otherwise.	6/2/2015 4:16 PM
389	Check the FeNo result or empirically treat for 2 weeks.	6/2/2015 4:16 PM
390	Treat May refer for methacholine	6/2/2015 4:15 PM
391	trial of medications	6/2/2015 4:10 PM
392	give another dose of SAB and repeat PFT	6/2/2015 4:09 PM
393	Start Controller medication and see them back in one month	6/2/2015 4:08 PM
394	repeat at next visit, treat other issues at this visit	6/2/2015 4:08 PM
395	also use FENO and if there is a 25% change in MMEF (FEF25-75)	6/2/2015 4:08 PM
396	Trial of medications	6/2/2015 4:08 PM
397	Will treat for asthma	6/2/2015 4:05 PM
398	Typically will either treat with systemic steroids if significantly obstructed to see if reversibility can be restored; if obstruction is minor, usually will look for other reasons for persistent cough	6/2/2015 4:05 PM
399	add a controller and repeat the test in one month	6/2/2015 4:04 PM
400	If high index suspicion, methacholine challenge	6/2/2015 4:02 PM
401	treat with appropriate medication and follow up test	6/2/2015 4:02 PM
402	send for a methacholine challenge or get full PFTs	6/2/2015 4:01 PM
403	Try bronchodilators for 2 weeks and repeat test	6/2/2015 4:00 PM
404	Send them for methacholine challenge test	6/2/2015 3:58 PM
405	Repeat when symptomatic or methacholine	6/2/2015 3:57 PM
406	empiric treatment with ICS. may also do short burst of systemic steroids to assess for reversibility if more significantly symptomatic	6/2/2015 3:56 PM
407	Full PFT	6/2/2015 3:56 PM
408	Use other information from history	6/2/2015 3:55 PM
409	look at the FEF 25-75	6/2/2015 3:54 PM
410	Methacholine	6/2/2015 3:54 PM
411	FeNO, methacholine.	6/2/2015 3:52 PM
412	2 week trial of asthma medication	6/2/2015 3:50 PM
413	Consider provocation testing	6/2/2015 3:50 PM
414	Trial of BD, etc	6/2/2015 3:50 PM
415	Perform a methacholine challenge	6/2/2015 3:48 PM
416	Treat empirically	6/2/2015 3:46 PM
417	Treat empirically.	6/2/2015 3:45 PM
418	check NO trial of medications and re-check	6/2/2015 3:42 PM
419	methacholine	6/2/2015 3:41 PM
420	Start ICS	6/2/2015 3:41 PM
421	treat	6/2/2015 3:40 PM
422	begin ICs or oral steroid and return in 2 weeks	6/2/2015 3:40 PM

423	two to four weeks of inhaled steroid, then retest. Occasionally will consider oral steroid -- 40 mg prednisone per day for 5 days, then 20 a day for 10 days. Repeat pfts.	6/2/2015 3:38 PM
424	Start inhaled steroids or increase dose anyway.	6/2/2015 3:37 PM
425	sometimes will administer another nebulizer dose (2.5mg each time) or perform methacholine challenge or exercise challenge depending on the history	6/2/2015 3:37 PM
426	empirical trial of controllers for 30 days with repeat pre/post albuterol PFT	6/2/2015 3:37 PM
427	repeat testing at another time	6/2/2015 3:35 PM
428	give peak flow meter for home use	6/2/2015 3:35 PM
429	MIC	6/2/2015 3:35 PM
430	nothing	6/2/2015 3:34 PM
431	still treat them	6/2/2015 3:31 PM
432	Will treat empirically with inhaled steroid. If no improvement then will have patient return for a methacholine challenge.	6/2/2015 3:31 PM
433	7 day course of prednisone or 6 week bid steroid inhlaer	6/2/2015 3:31 PM
434	Methacholine challenge	6/2/2015 3:31 PM
435	Methacholine challenge	6/2/2015 3:30 PM
436	methacholine challenge	6/2/2015 3:29 PM
437	evaluate symptomatic improvement	6/2/2015 3:29 PM
438	Jet neb	6/2/2015 3:28 PM
439	trial of bronchodilator 20 minutes before exercise	6/2/2015 3:28 PM
440	Steroid burst and/or FENO	6/2/2015 3:27 PM
441	Trial of therapy	6/2/2015 3:27 PM
442	confirm no use of bronchodilators pre test, consider with draw controllers and restudy. methacholine challenge	6/2/2015 3:27 PM
443	peak flows at home	6/2/2015 3:26 PM
444	Repeat with 4 puffs of MDI. Send out for full spirometry.	6/2/2015 3:25 PM
445	Treat clinically, consider formal PFT's, methacholine or mannitol challenge.	6/2/2015 3:24 PM
446	Check bid peak flows, consider exercise challenge or methacholine challenge.	6/2/2015 3:23 PM
447	treat with medications and follow up with another pft in the future	6/2/2015 3:23 PM
448	Repeat at next visit	6/2/2015 3:23 PM
449	Order MCT	6/2/2015 3:23 PM
450	trial of ICS or methcholine challenge	6/2/2015 3:23 PM
451	Begin oral steroids and repeat spirometry in 10 days	6/2/2015 3:22 PM
452	consider empiric treatment with steroid or other medication	6/2/2015 3:21 PM

Q12 Which patients receive bronchodilator reversibility tests? (Check All That Apply)

Answered: 488 Skipped: 8

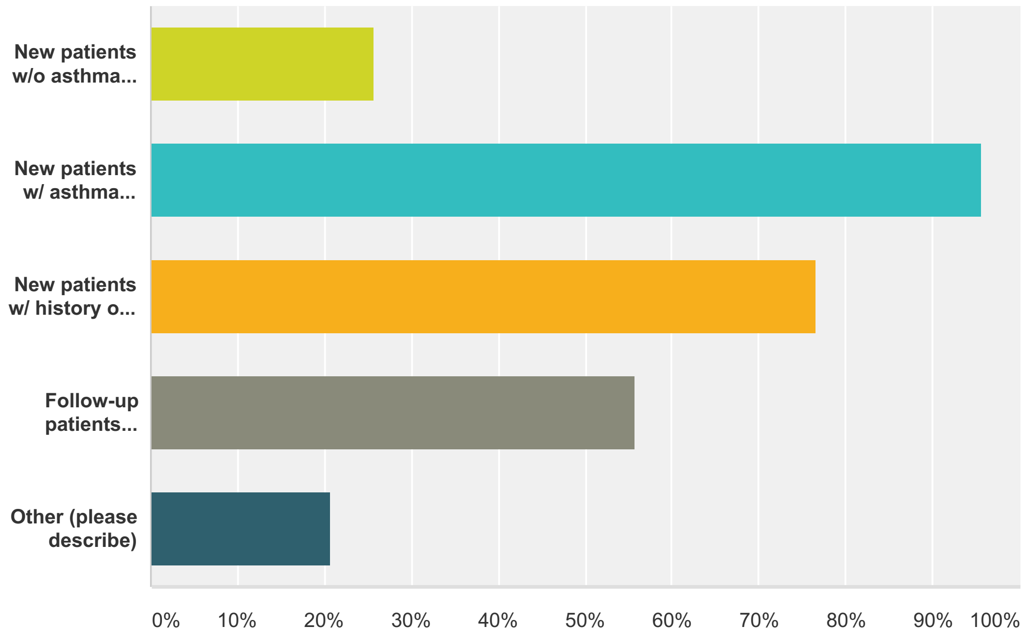

Answer Choices	Responses	
New patients w/o asthma symptoms to detect subclinical asthma	**25.61%**	125
New patients w/ asthma symptoms to aid in diagnosis	**95.70%**	467
New patients w/ history of asthma to assess initial asthma control	**76.64%**	374
Follow-up patients w/asthma to assess follow-up asthma control	**55.74%**	272
Other (please describe)	**20.70%**	101
**Total Respondents: 488**		

#	Other (please describe)	Date
1	pts with chronic cough AllCOPD pts Pts with acute exacerbation of asthma to see the degree of obstruction and also whether they respond to Albuterol	6/18/2015 5:24 PM
2	If baseline is normal, we do not perform post	6/16/2015 9:36 PM
3	sick patients with acute symptoms	6/16/2015 8:33 PM
4	WE HAVE ONE AIRWAY WHICH STARTS IN THE SINUSES AND ENDS AT THE BASE OF THE LUNGS SO ALL PATIENTS WITH UPPER AND/OR LOWER AIRWAY SYMPTOMS RECEIVE A BRT	6/16/2015 8:00 PM
5	Exacerbation	6/16/2015 7:01 PM
6	Patient with shortness of breath - unknown dx	6/16/2015 6:00 PM
7	patients in whom I am looking for reversibility of low numbers	6/16/2015 1:22 PM
8	evaluation of a cough or dyspnea	6/16/2015 12:55 PM

9	It is decided on a case by case basis depending on symptoms. Done to determine degree of reversibility and to gauge response to meds.	6/16/2015 11:33 AM
10	I use it for some asthmatic and COPD patients when the question of diagnosis is is in dispute.	6/16/2015 10:38 AM
11	Depends on the clinical situation. These answer choices do not allow for clinical judgement that would occur with each situation. Every new or follow up asthma patient does not need it, but some will.	6/16/2015 9:45 AM
12	Follow up patients may just have prebronchodilator spirometry unless it has been >1 year since last test	6/16/2015 9:14 AM
13	Generally new patients / return patients in whom the clinical history is unclear and /or treatment is not working.	6/16/2015 9:12 AM
14	This isn't really an "other" but --We do peak flows as vital signs on all patients able to do them. This has often been valuable when a patient without airway disease has a "cold" with cough--and peak flow is significantly decreased. %	6/14/2015 7:25 PM
15	new patients with pulmonary symptoms that may or may not be asthma	6/12/2015 12:27 PM
16	To assess spacer/MDI technique and to demonstrate that improved inhaler use will improve symptoms.	6/11/2015 9:23 AM
17	Patients having an asthma exacerbation	6/10/2015 2:53 PM
18	New patients with asthma symptoms but who have normal baseline spirometry. We routinely find that they bronchodilate well, and over the long term on ICS therapy have reduced symptoms with improved spirometry.	6/10/2015 2:00 PM
19	new patients who have a diagnosis of asthma but diagnosis is uncertain.	6/10/2015 11:26 AM
20	Usually only use if unclear a patient has asthma.	6/10/2015 11:25 AM
21	Sometimes in patients having asthma flares - helps to determine whether oral steroids are needed vs just increasing ICS. Also provides opportunity to assess inhaler technique.	6/10/2015 8:58 AM
22	New patients who are atopic (allergic rhinitis, etc) who report any respiratory symptoms--i.e. cough, chest tightness, wheezing, etc.	6/10/2015 6:16 AM
23	Asthma patient with exacerbation	6/9/2015 7:51 PM
24	eval of dyspnea	6/9/2015 7:48 PM
25	obstruction on spirometry, concave expiratory curves.	6/9/2015 7:10 PM
26	ID patients incorrectly dx with asthma who have inspiratory dyspnea and VCD instead.	6/9/2015 6:57 PM
27	Followup patients in distress in order to determine how much of their comprimise is reversible with bronchodilators and how aggresive I might need to get (or not get) with steroids.	6/9/2015 6:13 PM
28	Patients with an appropriate respiratory diagnosis (ie cough)	6/9/2015 5:08 PM
29	study patients or some new patients with cough or asthma symptoms and mildly low spirometry (80-90%) to see if bronchospasm/asthma is present (when not obvious by history).	6/9/2015 5:03 PM
30	clinical research	6/9/2015 5:00 PM
31	Patients with nocturnal awakening and or other Asthms sxs.	6/9/2015 4:47 PM
32	as respirologists we test COPD and suspect COPD patient all the time with BD reversibility	6/9/2015 3:27 PM
33	asthma patients under poor control. new patients with cough	6/9/2015 3:22 PM
34	new atients with exertional dyspnea; new patients with chronic cough and normal CXR.	6/9/2015 3:16 PM
35	Most patients who show evidence of obstruction not previously treated with ICS.	6/9/2015 3:08 PM
36	new or follow-up paitents with suggestive history and obstructive spirometry in whom knowledge of post-BD reversability will change mgt	6/9/2015 2:43 PM
37	Abnormal baseline spirometry.	6/9/2015 2:39 PM
38	New pts with without asthma that have symptoms suggestive of asthma.	6/9/2015 2:22 PM
39	SOMETIMES FOR ACUTE EXACERBATION TO ASSESS RESPONSE TO B AGONIST AND MAY BE DECIDE STEROID BURST DOSAGE AND DURATION.	6/9/2015 1:27 PM

40	chronic cough patients without previous diagnosis of asthma	6/9/2015 1:11 PM
41	For new patients, this is done if I need to make a diagnosis or if I want to know their level of reversibility. Many new patients are treated with new meds and return for follow up several weeks later to assess without a reversibility test. Follow up patients may have the testing done if it is clinically indicated but this is less common for me.	6/9/2015 1:10 PM
42	Am using bronchodilator tests for followup less now that have easy availability to FeNO	6/9/2015 7:24 AM
43	Patient with mixed obstructive/restrictive lung tests	6/8/2015 11:34 AM
44	Patients with histories suspicious of asthma without objectively confirmed reversible obstruction or observed improvement with standard asthem therapy	6/7/2015 2:37 PM
45	New patients with hx of childhood asthma, new patients with history of a chronic cough, patients with history of EIB	6/4/2015 6:33 PM
46	pts with complaint of cough	6/4/2015 8:46 AM
47	Patients with chronic cough	6/4/2015 7:17 AM
48	Asthma sick call patients to determine the severity of exacerbations	6/3/2015 9:37 PM
49	Based on clinical history - if there are symptoms not well controlled. For either new or follow up pts. For significant decreases in lung function in established patients	6/3/2015 2:43 PM
50	patients with an unexplained cough	6/3/2015 1:10 PM
51	If they are old enough to perform the test.	6/3/2015 1:10 PM
52	COPD patients	6/3/2015 10:35 AM
53	Asthma diagnosis with acute change in symptoms	6/3/2015 9:59 AM
54	Follow up patients with asthma that have symptoms of an asthma flare or just a cough or active wheezing	6/3/2015 9:31 AM
55	follow up patients may not get spirometry every visit	6/3/2015 8:38 AM
56	any patient where diagnosis is doubt	6/3/2015 8:01 AM
57	Patients with respiratory symptoms	6/3/2015 8:01 AM
58	Symptomatic patints with abnormal spirometries	6/3/2015 7:53 AM
59	patients with clinical control but severe obstruction	6/3/2015 6:39 AM
60	Someone with obstruction on the baseline test in whom I would like to determined if there is a ß-agonist reversible component or not.	6/3/2015 6:31 AM
61	Patients in which the diagnosis of asthma is uncertain or who are not responding to therapy as expected	6/2/2015 11:11 PM
62	patients initially thought to have EIB who might actually have asthma	6/2/2015 10:37 PM
63	FU patients with low lung function	6/2/2015 10:18 PM
64	Follow up patients with a lower FEV1. Pts who are considering a clinical research study.	6/2/2015 9:52 PM
65	Select patients with obstruction present on prebronchodilation	6/2/2015 9:49 PM
66	symtoms that suggest pulmonary disease	6/2/2015 9:03 PM
67	When patients are having acute symptoms or asthma - like symptoms to differentiate between asthma and non-asthma	6/2/2015 8:43 PM
68	New patients with history of asthma to confirm diagnosis	6/2/2015 8:07 PM
69	where the diagnosis of asthma is unclear	6/2/2015 8:03 PM
70	Might not get postbronchodilator study if baseline spirometry is acceptable in some if the above clinical scenarios	6/2/2015 7:59 PM
71	Any follow up patients, with evidence of persistent airflow obstruction despite infrequent symptoms, to see if has component of fixed airflow obstruction.	6/2/2015 7:13 PM
72	patients with cough	6/2/2015 7:10 PM

73	When patients come to office acutely ill, to determine whether to prescribe steroids or just reurn to baseline program.	6/2/2015 7:07 PM
74	In patients with chronic rhinitis I often do spirometry because of the higher incidence of asymptomatic asthma.	6/2/2015 7:04 PM
75	Part of work up for chronic cough / chest tightness	6/2/2015 7:03 PM
76	Repeat study on follow up when assessing steroid responsiveness	6/2/2015 6:56 PM
77	cough	6/2/2015 6:18 PM
78	for followup patients who are not controlled I may do bronchospasm challenge to confirm they are reversible, and, if so, this usually leads into a discussion that reveals that they are not taking their medicines as prescribed or we need to review and correct inhaler technique	6/2/2015 6:14 PM
79	Any new patient with a history of bronchitis or pneumonia or clinical symptoms of SOB, wheeze or cough.	6/2/2015 6:10 PM
80	patients with abnormal spirometry at baseline	6/2/2015 5:49 PM
81	Symptoms.	6/2/2015 5:36 PM
82	Post exercise challenges to help distinguish EIA from VCD	6/2/2015 5:19 PM
83	new patients with no overt history of asthma but markedly decreased peak flow	6/2/2015 5:00 PM
84	Follow up patients with a specific reason - ie, a major med change.	6/2/2015 4:18 PM
85	Rarely only on patients who have hard to control issues.	6/2/2015 4:16 PM
86	Follow-up patients previously with good control now with exacerbations.	6/2/2015 4:16 PM
87	chronic cough	6/2/2015 4:09 PM
88	Follow up patients with new symptoms	6/2/2015 4:08 PM
89	Most often are new patients with a chronic cough and abnormal spirometry.	6/2/2015 4:05 PM
90	any patient with cough/wheeze/dyspnea, poor exercise tolerance, even fatigue in someone with allergies	6/2/2015 3:50 PM
91	Help clarify between asthma and COPD	6/2/2015 3:49 PM
92	New patients with suspicion of vocal cord dysfunction to rule out atypical asthma	6/2/2015 3:48 PM
93	but not every patient every time	6/2/2015 3:40 PM
94	any patient with pfts below normal, or in the low normal range	6/2/2015 3:38 PM
95	Are we missing the main point? What % of patients DON"T get seen or followed by specialists in the real world.	6/2/2015 3:37 PM
96	if medication dosage adjustment change or patient going into the military, if patient on immunotherapy and not needing medication. Or if insurance mandates a switch of medication.	6/2/2015 3:35 PM
97	Will assess indivudals with history of asthma that has been "outgrown" to make sure that they do not indeed have subclinical asthma.	6/2/2015 3:31 PM
98	only if asthma is not certain but suspected	6/2/2015 3:30 PM
99	questionable respiratory symptoms to have results serving as baseline, flow volume loop for possible obstructive, VCD, etc	6/2/2015 3:26 PM
100	Patients with unexplained dyspnea in whom no PFT data might be available	6/2/2015 3:25 PM
101	Completely depends on history and individual patient factors. All new patients with abnormal spirometry is the most common category. Follow up patietns with abnormal or below baseline spiro.	6/2/2015 3:24 PM

Q13 What proportion of patients in each category do you perform bronchodilator reversibility testing for? (0-100% for each category)

Answered: 468 Skipped: 28

Answer Choices	Responses	
New patients w/o asthma symptoms to detect subclinical asthma:	**90.38%**	423
New patients w/ asthma symptoms to aid in diagnosis:	**99.36%**	465
New patients w/ history of asthma to assess initial asthma control:	**95.51%**	447
Follow-up patients w/ asthma to assess follow-up asthma control:	**92.31%**	432

#	New patients w/o asthma symptoms to detect subclinical asthma:	Date
1	80	6/19/2015 12:50 PM
2	0	6/19/2015 12:32 PM
3	100	6/18/2015 5:24 PM
4	5	6/18/2015 4:49 PM
5	0	6/18/2015 12:03 PM
6	35	6/18/2015 9:56 AM
7	10	6/17/2015 4:53 PM
8	10	6/17/2015 4:01 PM
9	0	6/17/2015 2:37 PM
10	35	6/17/2015 10:54 AM
11	10	6/17/2015 9:43 AM
12	25	6/16/2015 9:38 PM
13	0	6/16/2015 9:36 PM
14	0	6/16/2015 9:00 PM
15	10	6/16/2015 8:33 PM
16	100%	6/16/2015 8:00 PM
17	50%	6/16/2015 7:01 PM
18	0	6/16/2015 6:00 PM
19	0	6/16/2015 5:43 PM
20	0	6/16/2015 4:09 PM
21	0	6/16/2015 3:37 PM
22	0	6/16/2015 2:42 PM
23	0	6/16/2015 2:24 PM
24	80	6/16/2015 2:10 PM
25	100	6/16/2015 2:00 PM

26	5	6/16/2015 1:22 PM
27	0	6/16/2015 1:01 PM
28	20	6/16/2015 12:55 PM
29	85	6/16/2015 12:42 PM
30	20	6/16/2015 12:33 PM
31	0	6/16/2015 12:22 PM
32	0	6/16/2015 11:55 AM
33	0	6/16/2015 11:36 AM
34	0	6/16/2015 11:33 AM
35	25	6/16/2015 11:33 AM
36	0	6/16/2015 11:18 AM
37	0	6/16/2015 11:00 AM
38	1	6/16/2015 10:38 AM
39	5	6/16/2015 10:12 AM
40	5	6/16/2015 10:05 AM
41	0	6/16/2015 10:05 AM
42	100	6/16/2015 9:56 AM
43	0	6/16/2015 9:30 AM
44	0	6/16/2015 9:29 AM
45	10	6/16/2015 9:23 AM
46	10	6/16/2015 9:15 AM
47	100	6/16/2015 9:14 AM
48	0	6/16/2015 9:12 AM
49	0	6/16/2015 9:12 AM
50	10	6/16/2015 9:10 AM
51	85	6/16/2015 9:10 AM
52	100	6/16/2015 9:10 AM
53	80	6/16/2015 9:09 AM
54	0	6/16/2015 9:09 AM
55	90	6/16/2015 9:06 AM
56	10	6/16/2015 9:05 AM
57	50	6/15/2015 8:44 PM
58	0	6/14/2015 8:35 PM
59	5% at most	6/14/2015 7:25 PM
60	0	6/14/2015 4:52 PM
61	0	6/14/2015 1:43 AM
62	0	6/13/2015 8:39 PM
63	10	6/12/2015 9:10 PM

64	2%	6/12/2015 10:34 AM
65	75	6/12/2015 9:05 AM
66	20	6/12/2015 7:51 AM
67	15	6/12/2015 6:19 AM
68	10%	6/11/2015 11:34 AM
69	50	6/11/2015 9:23 AM
70	<5%	6/11/2015 12:00 AM
71	0	6/10/2015 5:29 PM
72	10	6/10/2015 3:17 PM
73	10%	6/10/2015 2:53 PM
74	5	6/10/2015 2:43 PM
75	5	6/10/2015 2:19 PM
76	5	6/10/2015 2:00 PM
77	0	6/10/2015 1:29 PM
78	0	6/10/2015 1:19 PM
79	0	6/10/2015 1:13 PM
80	0	6/10/2015 1:04 PM
81	0	6/10/2015 12:50 PM
82	10	6/10/2015 11:41 AM
83	60	6/10/2015 11:32 AM
84	1	6/10/2015 11:26 AM
85	0	6/10/2015 11:25 AM
86	0	6/10/2015 10:26 AM
87	0	6/10/2015 9:48 AM
88	0	6/10/2015 9:14 AM
89	0	6/10/2015 8:58 AM
90	60%	6/10/2015 8:37 AM
91	0	6/10/2015 8:08 AM
92	5	6/10/2015 6:16 AM
93	0	6/10/2015 6:04 AM
94	0	6/10/2015 5:55 AM
95	1%	6/10/2015 1:54 AM
96	100	6/10/2015 12:39 AM
97	10	6/9/2015 10:57 PM
98	100%	6/9/2015 10:28 PM
99	0	6/9/2015 9:56 PM
100	0	6/9/2015 9:26 PM
101	10	6/9/2015 8:46 PM

102	0	6/9/2015 8:03 PM
103	0	6/9/2015 7:51 PM
104	30	6/9/2015 7:48 PM
105	20	6/9/2015 7:48 PM
106	100	6/9/2015 7:17 PM
107	25	6/9/2015 6:57 PM
108	10	6/9/2015 6:55 PM
109	10	6/9/2015 6:27 PM
110	20	6/9/2015 6:13 PM
111	10	6/9/2015 6:00 PM
112	100	6/9/2015 5:25 PM
113	2	6/9/2015 5:23 PM
114	0	6/9/2015 5:21 PM
115	0	6/9/2015 5:20 PM
116	0 (insurance will not reimburse)	6/9/2015 5:08 PM
117	0	6/9/2015 5:03 PM
118	10	6/9/2015 5:00 PM
119	0	6/9/2015 4:55 PM
120	50	6/9/2015 4:53 PM
121	0	6/9/2015 4:47 PM
122	20	6/9/2015 4:41 PM
123	90	6/9/2015 4:24 PM
124	5	6/9/2015 4:06 PM
125	5%	6/9/2015 4:03 PM
126	0%	6/9/2015 3:56 PM
127	0	6/9/2015 3:41 PM
128	50%	6/9/2015 3:38 PM
129	0	6/9/2015 3:32 PM
130	50	6/9/2015 3:27 PM
131	0	6/9/2015 3:25 PM
132	80	6/9/2015 3:22 PM
133	0	6/9/2015 3:16 PM
134	0	6/9/2015 3:10 PM
135	0	6/9/2015 3:08 PM
136	0	6/9/2015 2:55 PM
137	20	6/9/2015 2:50 PM
138	0	6/9/2015 2:49 PM
139	5%	6/9/2015 2:39 PM

140	5	6/9/2015 2:36 PM
141	10%	6/9/2015 2:32 PM
142	0	6/9/2015 2:31 PM
143	90	6/9/2015 2:29 PM
144	0	6/9/2015 2:25 PM
145	0	6/9/2015 2:23 PM
146	0	6/9/2015 2:22 PM
147	0	6/9/2015 2:15 PM
148	5	6/9/2015 1:58 PM
149	70	6/9/2015 1:58 PM
150	0	6/9/2015 1:44 PM
151	10	6/9/2015 1:38 PM
152	100	6/9/2015 1:29 PM
153	50	6/9/2015 1:27 PM
154	0	6/9/2015 1:27 PM
155	0	6/9/2015 1:19 PM
156	90	6/9/2015 1:13 PM
157	0	6/9/2015 1:12 PM
158	5%	6/9/2015 1:12 PM
159	0	6/9/2015 1:11 PM
160	20	6/9/2015 1:11 PM
161	0	6/9/2015 1:10 PM
162	0	6/9/2015 1:07 PM
163	0	6/9/2015 7:41 AM
164	10	6/9/2015 7:24 AM
165	0	6/9/2015 4:56 AM
166	0	6/9/2015 1:59 AM
167	100	6/8/2015 8:34 PM
168	100	6/8/2015 1:36 PM
169	10	6/8/2015 12:10 PM
170	5%	6/8/2015 11:34 AM
171	0	6/8/2015 7:42 AM
172	0	6/7/2015 9:04 PM
173	0	6/7/2015 4:35 PM
174	0	6/7/2015 3:19 PM
175	100	6/7/2015 2:48 PM
176	1	6/7/2015 2:37 PM
177	1	6/7/2015 1:33 PM

178	10	6/7/2015 12:01 PM
179	0	6/6/2015 9:35 PM
180	0	6/6/2015 12:32 PM
181	25%	6/6/2015 12:16 AM
182	10	6/5/2015 8:41 PM
183	20%	6/5/2015 3:32 PM
184	0	6/5/2015 2:51 PM
185	10	6/5/2015 2:26 PM
186	50	6/5/2015 1:51 PM
187	0%	6/5/2015 12:29 PM
188	0	6/5/2015 10:34 AM
189	0	6/4/2015 9:32 PM
190	10	6/4/2015 6:33 PM
191	40	6/4/2015 5:46 PM
192	50	6/4/2015 4:30 PM
193	50%	6/4/2015 4:28 PM
194	10	6/4/2015 3:21 PM
195	0	6/4/2015 3:15 PM
196	90	6/4/2015 2:55 PM
197	5	6/4/2015 1:17 PM
198	0	6/4/2015 1:17 PM
199	0	6/4/2015 12:40 PM
200	0	6/4/2015 11:59 AM
201	0	6/4/2015 11:40 AM
202	0	6/4/2015 10:38 AM
203	0	6/4/2015 8:46 AM
204	0	6/4/2015 8:01 AM
205	100	6/4/2015 7:34 AM
206	0	6/4/2015 7:33 AM
207	10	6/4/2015 7:17 AM
208	0	6/4/2015 7:15 AM
209	10	6/4/2015 5:50 AM
210	0	6/3/2015 10:11 PM
211	5	6/3/2015 9:37 PM
212	0	6/3/2015 8:23 PM
213	50	6/3/2015 8:17 PM
214	0	6/3/2015 7:56 PM
215	0	6/3/2015 7:45 PM

216	0	6/3/2015 7:35 PM
217	10	6/3/2015 6:48 PM
218	100%	6/3/2015 5:09 PM
219	0	6/3/2015 4:55 PM
220	0	6/3/2015 4:54 PM
221	0	6/3/2015 4:42 PM
222	75	6/3/2015 4:37 PM
223	0	6/3/2015 4:14 PM
224	10	6/3/2015 3:37 PM
225	50	6/3/2015 3:06 PM
226	10	6/3/2015 2:43 PM
227	0	6/3/2015 2:16 PM
228	0	6/3/2015 2:08 PM
229	0	6/3/2015 1:59 PM
230	0	6/3/2015 1:56 PM
231	0	6/3/2015 1:10 PM
232	0	6/3/2015 1:10 PM
233	0	6/3/2015 1:06 PM
234	0	6/3/2015 12:56 PM
235	0	6/3/2015 12:49 PM
236	15	6/3/2015 12:07 PM
237	100	6/3/2015 11:46 AM
238	0	6/3/2015 11:45 AM
239	0	6/3/2015 11:27 AM
240	0	6/3/2015 11:15 AM
241	5	6/3/2015 11:06 AM
242	0	6/3/2015 11:03 AM
243	0	6/3/2015 10:35 AM
244	15	6/3/2015 10:34 AM
245	50	6/3/2015 10:26 AM
246	50	6/3/2015 10:21 AM
247	None	6/3/2015 10:18 AM
248	75%	6/3/2015 10:17 AM
249	0	6/3/2015 10:11 AM
250	0	6/3/2015 10:10 AM
251	0%	6/3/2015 10:08 AM
252	0%	6/3/2015 9:59 AM
253	10	6/3/2015 9:48 AM

254	1%	6/3/2015 9:31 AM
255	0	6/3/2015 9:28 AM
256	5	6/3/2015 8:43 AM
257	50	6/3/2015 8:40 AM
258	10	6/3/2015 8:38 AM
259	5	6/3/2015 8:19 AM
260	0	6/3/2015 8:14 AM
261	95	6/3/2015 8:13 AM
262	30	6/3/2015 8:11 AM
263	0	6/3/2015 8:10 AM
264	5	6/3/2015 8:09 AM
265	2	6/3/2015 8:01 AM
266	0	6/3/2015 8:01 AM
267	0	6/3/2015 7:53 AM
268	0	6/3/2015 7:50 AM
269	0	6/3/2015 7:45 AM
270	10	6/3/2015 7:33 AM
271	0	6/3/2015 6:39 AM
272	0	6/3/2015 6:31 AM
273	0	6/3/2015 6:23 AM
274	10	6/3/2015 6:05 AM
275	10	6/3/2015 5:49 AM
276	0	6/3/2015 4:09 AM
277	0	6/3/2015 3:47 AM
278	0	6/3/2015 2:45 AM
279	0	6/2/2015 11:59 PM
280	5	6/2/2015 11:11 PM
281	0	6/2/2015 10:51 PM
282	100	6/2/2015 10:43 PM
283	100	6/2/2015 10:37 PM
284	0	6/2/2015 10:24 PM
285	25	6/2/2015 10:18 PM
286	50	6/2/2015 9:59 PM
287	15	6/2/2015 9:59 PM
288	100	6/2/2015 9:52 PM
289	0	6/2/2015 9:49 PM
290	5	6/2/2015 9:39 PM
291	75	6/2/2015 9:30 PM

292	0	6/2/2015 9:26 PM
293	75	6/2/2015 9:20 PM
294	100	6/2/2015 9:16 PM
295	Approximately 30% of patients	6/2/2015 9:12 PM
296	0	6/2/2015 9:04 PM
297	20	6/2/2015 9:03 PM
298	10	6/2/2015 9:01 PM
299	5	6/2/2015 8:58 PM
300	0	6/2/2015 8:51 PM
301	0	6/2/2015 8:47 PM
302	10%	6/2/2015 8:45 PM
303	0	6/2/2015 8:43 PM
304	30	6/2/2015 8:42 PM
305	0	6/2/2015 8:38 PM
306	20	6/2/2015 8:36 PM
307	100	6/2/2015 8:19 PM
308	0	6/2/2015 8:19 PM
309	0	6/2/2015 8:07 PM
310	0	6/2/2015 8:07 PM
311	10%	6/2/2015 8:03 PM
312	5	6/2/2015 7:59 PM
313	0	6/2/2015 7:57 PM
314	20	6/2/2015 7:56 PM
315	0	6/2/2015 7:47 PM
316	5	6/2/2015 7:42 PM
317	100	6/2/2015 7:31 PM
318	0	6/2/2015 7:25 PM
319	80	6/2/2015 7:13 PM
320	0	6/2/2015 7:10 PM
321	0	6/2/2015 7:07 PM
322	0	6/2/2015 7:07 PM
323	75	6/2/2015 7:04 PM
324	0	6/2/2015 7:03 PM
325	0	6/2/2015 6:56 PM
326	0	6/2/2015 6:42 PM
327	50	6/2/2015 6:42 PM
328	100	6/2/2015 6:30 PM
329	25	6/2/2015 6:28 PM

330	0	6/2/2015 6:14 PM
331	100%	6/2/2015 6:13 PM
332	25	6/2/2015 6:10 PM
333	100	6/2/2015 6:03 PM
334	0	6/2/2015 5:54 PM
335	10	6/2/2015 5:49 PM
336	15	6/2/2015 5:47 PM
337	20	6/2/2015 5:47 PM
338	0-2%	6/2/2015 5:40 PM
339	0	6/2/2015 5:35 PM
340	0	6/2/2015 5:32 PM
341	20	6/2/2015 5:31 PM
342	0%	6/2/2015 5:30 PM
343	0	6/2/2015 5:19 PM
344	20	6/2/2015 5:09 PM
345	0	6/2/2015 5:09 PM
346	0	6/2/2015 5:09 PM
347	0	6/2/2015 5:06 PM
348	0	6/2/2015 5:05 PM
349	10	6/2/2015 5:00 PM
350	100	6/2/2015 4:54 PM
351	100	6/2/2015 4:54 PM
352	0	6/2/2015 4:49 PM
353	0	6/2/2015 4:46 PM
354	5	6/2/2015 4:43 PM
355	0	6/2/2015 4:42 PM
356	100	6/2/2015 4:32 PM
357	1%	6/2/2015 4:30 PM
358	10	6/2/2015 4:24 PM
359	100	6/2/2015 4:23 PM
360	20	6/2/2015 4:18 PM
361	0	6/2/2015 4:18 PM
362	2	6/2/2015 4:17 PM
363	0	6/2/2015 4:16 PM
364	0	6/2/2015 4:10 PM
365	0	6/2/2015 4:08 PM
366	0	6/2/2015 4:08 PM
367	100	6/2/2015 4:08 PM

368	0	6/2/2015 4:08 PM
369	0	6/2/2015 4:05 PM
370	0	6/2/2015 4:05 PM
371	0	6/2/2015 4:04 PM
372	0	6/2/2015 4:02 PM
373	20	6/2/2015 4:02 PM
374	20	6/2/2015 4:01 PM
375	0	6/2/2015 4:01 PM
376	10	6/2/2015 4:00 PM
377	0	6/2/2015 3:59 PM
378	95%	6/2/2015 3:58 PM
379	0	6/2/2015 3:57 PM
380	0	6/2/2015 3:56 PM
381	0	6/2/2015 3:56 PM
382	50%	6/2/2015 3:56 PM
383	5	6/2/2015 3:55 PM
384	0	6/2/2015 3:54 PM
385	30	6/2/2015 3:54 PM
386	0	6/2/2015 3:52 PM
387	0	6/2/2015 3:50 PM
388	0	6/2/2015 3:50 PM
389	20	6/2/2015 3:49 PM
390	0	6/2/2015 3:48 PM
391	0	6/2/2015 3:46 PM
392	0	6/2/2015 3:45 PM
393	30	6/2/2015 3:42 PM
394	0	6/2/2015 3:41 PM
395	25	6/2/2015 3:41 PM
396	15	6/2/2015 3:40 PM
397	0	6/2/2015 3:40 PM
398	0	6/2/2015 3:38 PM
399	25	6/2/2015 3:37 PM
400	100%	6/2/2015 3:37 PM
401	0	6/2/2015 3:35 PM
402	0	6/2/2015 3:35 PM
403	20	6/2/2015 3:35 PM
404	0	6/2/2015 3:34 PM
405	0	6/2/2015 3:31 PM

406	0	6/2/2015 3:31 PM
407	0	6/2/2015 3:31 PM
408	0	6/2/2015 3:30 PM
409	0	6/2/2015 3:29 PM
410	10	6/2/2015 3:28 PM
411	50	6/2/2015 3:28 PM
412	5	6/2/2015 3:27 PM
413	0	6/2/2015 3:27 PM
414	100	6/2/2015 3:26 PM
415	20	6/2/2015 3:25 PM
416	0	6/2/2015 3:24 PM
417	0	6/2/2015 3:23 PM
418	80	6/2/2015 3:23 PM
419	0	6/2/2015 3:23 PM
420	0	6/2/2015 3:23 PM
421	0	6/2/2015 3:23 PM
422	0	6/2/2015 3:23 PM
423	0	6/2/2015 3:22 PM

#	New patients w/ asthma symptoms to aid in diagnosis:	Date
1	45	6/19/2015 4:48 PM
2	80	6/19/2015 12:50 PM
3	50	6/19/2015 12:32 PM
4	100	6/18/2015 5:24 PM
5	35	6/18/2015 4:49 PM
6	75	6/18/2015 12:03 PM
7	50	6/18/2015 9:56 AM
8	100	6/17/2015 4:53 PM
9	100	6/17/2015 4:01 PM
10	25	6/17/2015 2:37 PM
11	35	6/17/2015 10:54 AM
12	100	6/17/2015 9:43 AM
13	25	6/16/2015 9:38 PM
14	90	6/16/2015 9:36 PM
15	20	6/16/2015 9:00 PM
16	10-20	6/16/2015 8:33 PM
17	100%	6/16/2015 8:00 PM
18	100%	6/16/2015 7:01 PM
19	100	6/16/2015 6:00 PM

20	75	6/16/2015 5:43 PM
21	20	6/16/2015 4:09 PM
22	90	6/16/2015 3:37 PM
23	100	6/16/2015 2:42 PM
24	30	6/16/2015 2:24 PM
25	50	6/16/2015 2:10 PM
26	100	6/16/2015 2:00 PM
27	30	6/16/2015 1:36 PM
28	100	6/16/2015 1:22 PM
29	90	6/16/2015 1:01 PM
30	80	6/16/2015 12:55 PM
31	95	6/16/2015 12:42 PM
32	100	6/16/2015 12:33 PM
33	60	6/16/2015 12:22 PM
34	90	6/16/2015 11:55 AM
35	75	6/16/2015 11:36 AM
36	95	6/16/2015 11:33 AM
37	25	6/16/2015 11:33 AM
38	50	6/16/2015 11:18 AM
39	100	6/16/2015 11:00 AM
40	90	6/16/2015 10:58 AM
41	1	6/16/2015 10:38 AM
42	100	6/16/2015 10:14 AM
43	30	6/16/2015 10:12 AM
44	40	6/16/2015 10:05 AM
45	80%	6/16/2015 10:05 AM
46	100	6/16/2015 9:56 AM
47	95	6/16/2015 9:30 AM
48	85	6/16/2015 9:29 AM
49	10	6/16/2015 9:23 AM
50	90	6/16/2015 9:15 AM
51	100	6/16/2015 9:14 AM
52	0	6/16/2015 9:12 AM
53	100	6/16/2015 9:12 AM
54	100	6/16/2015 9:10 AM
55	100	6/16/2015 9:10 AM
56	80	6/16/2015 9:09 AM
57	30	6/16/2015 9:09 AM

58	100	6/16/2015 9:06 AM
59	95	6/16/2015 9:05 AM
60	100%	6/15/2015 8:48 PM
61	100	6/15/2015 8:44 PM
62	100	6/14/2015 9:41 PM
63	90	6/14/2015 8:35 PM
64	90%	6/14/2015 7:25 PM
65	20	6/14/2015 4:52 PM
66	100	6/14/2015 1:43 AM
67	30%	6/13/2015 8:39 PM
68	100100	6/12/2015 9:10 PM
69	95%	6/12/2015 12:27 PM
70	60 %	6/12/2015 10:34 AM
71	25	6/12/2015 9:05 AM
72	30	6/12/2015 7:51 AM
73	25	6/12/2015 6:19 AM
74	10%	6/11/2015 11:34 AM
75	10	6/11/2015 9:23 AM
76	80%	6/11/2015 12:00 AM
77	80	6/10/2015 5:29 PM
78	30	6/10/2015 3:17 PM
79	50%	6/10/2015 2:53 PM
80	5	6/10/2015 2:43 PM
81	60	6/10/2015 2:19 PM
82	40	6/10/2015 2:00 PM
83	50	6/10/2015 1:29 PM
84	80	6/10/2015 1:19 PM
85	50	6/10/2015 1:13 PM
86	0	6/10/2015 1:04 PM
87	75	6/10/2015 12:50 PM
88	15	6/10/2015 11:58 AM
89	100	6/10/2015 11:41 AM
90	90	6/10/2015 11:32 AM
91	100	6/10/2015 11:26 AM
92	50	6/10/2015 11:25 AM
93	25	6/10/2015 10:26 AM
94	100	6/10/2015 9:48 AM
95	100	6/10/2015 9:14 AM

96	100	6/10/2015 8:58 AM
97	60%	6/10/2015 8:37 AM
98	100	6/10/2015 8:08 AM
99	90	6/10/2015 7:53 AM
100	100	6/10/2015 6:16 AM
101	100	6/10/2015 6:04 AM
102	20	6/10/2015 5:55 AM
103	100%	6/10/2015 1:54 AM
104	100	6/10/2015 12:39 AM
105	100	6/9/2015 10:57 PM
106	100%	6/9/2015 10:28 PM
107	100	6/9/2015 9:56 PM
108	50	6/9/2015 9:26 PM
109	70	6/9/2015 8:46 PM
110	100	6/9/2015 8:03 PM
111	10	6/9/2015 7:51 PM
112	100	6/9/2015 7:48 PM
113	60	6/9/2015 7:48 PM
114	100	6/9/2015 7:17 PM
115	100	6/9/2015 6:57 PM
116	10	6/9/2015 6:55 PM
117	100	6/9/2015 6:27 PM
118	90	6/9/2015 6:13 PM
119	30	6/9/2015 6:00 PM
120	100	6/9/2015 5:25 PM
121	100	6/9/2015 5:23 PM
122	100	6/9/2015 5:21 PM
123	100	6/9/2015 5:20 PM
124	100	6/9/2015 5:08 PM
125	20	6/9/2015 5:03 PM
126	70	6/9/2015 5:00 PM
127	70	6/9/2015 4:55 PM
128	80	6/9/2015 4:53 PM
129	100	6/9/2015 4:47 PM
130	10	6/9/2015 4:41 PM
131	90	6/9/2015 4:24 PM
132	90	6/9/2015 4:06 PM
133	100%	6/9/2015 4:03 PM

134	100%	6/9/2015 3:56 PM
135	25	6/9/2015 3:44 PM
136	100	6/9/2015 3:41 PM
137	80-90%	6/9/2015 3:38 PM
138	100	6/9/2015 3:32 PM
139	100	6/9/2015 3:27 PM
140	100	6/9/2015 3:25 PM
141	100	6/9/2015 3:22 PM
142	100	6/9/2015 3:16 PM
143	75	6/9/2015 3:10 PM
144	50	6/9/2015 3:08 PM
145	50	6/9/2015 2:55 PM
146	80	6/9/2015 2:50 PM
147	20	6/9/2015 2:49 PM
148	70%	6/9/2015 2:39 PM
149	70	6/9/2015 2:36 PM
150	100%	6/9/2015 2:32 PM
151	10	6/9/2015 2:31 PM
152	90	6/9/2015 2:29 PM
153	100	6/9/2015 2:25 PM
154	40	6/9/2015 2:23 PM
155	30	6/9/2015 2:22 PM
156	90	6/9/2015 2:15 PM
157	100	6/9/2015 1:58 PM
158	50	6/9/2015 1:58 PM
159	15	6/9/2015 1:48 PM
160	50	6/9/2015 1:44 PM
161	20	6/9/2015 1:40 PM
162	90	6/9/2015 1:38 PM
163	100	6/9/2015 1:29 PM
164	100	6/9/2015 1:27 PM
165	100	6/9/2015 1:27 PM
166	100	6/9/2015 1:19 PM
167	100	6/9/2015 1:13 PM
168	100	6/9/2015 1:12 PM
169	90%	6/9/2015 1:12 PM
170	90	6/9/2015 1:11 PM
171	25	6/9/2015 1:11 PM

172	10	6/9/2015 1:10 PM
173	99	6/9/2015 1:07 PM
174	75	6/9/2015 7:41 AM
175	60	6/9/2015 7:24 AM
176	40	6/9/2015 4:56 AM
177	100	6/9/2015 1:59 AM
178	100	6/8/2015 8:34 PM
179	100	6/8/2015 1:36 PM
180	30	6/8/2015 12:10 PM
181	50%	6/8/2015 11:34 AM
182	100	6/8/2015 7:42 AM
183	100	6/7/2015 9:04 PM
184	100	6/7/2015 4:35 PM
185	100	6/7/2015 3:19 PM
186	100	6/7/2015 2:48 PM
187	10	6/7/2015 2:37 PM
188	50	6/7/2015 1:33 PM
189	100	6/7/2015 12:01 PM
190	100	6/6/2015 9:35 PM
191	100	6/6/2015 12:32 PM
192	100%	6/6/2015 12:16 AM
193	10	6/5/2015 8:41 PM
194	35%	6/5/2015 3:32 PM
195	100	6/5/2015 2:51 PM
196	90	6/5/2015 2:26 PM
197	100	6/5/2015 1:51 PM
198	100%	6/5/2015 12:29 PM
199	100	6/5/2015 12:03 PM
200	100	6/5/2015 10:34 AM
201	100%	6/5/2015 4:40 AM
202	50	6/4/2015 9:32 PM
203	90	6/4/2015 6:33 PM
204	60	6/4/2015 5:46 PM
205	100	6/4/2015 4:30 PM
206	100%	6/4/2015 4:28 PM
207	99	6/4/2015 3:21 PM
208	70	6/4/2015 3:15 PM
209	90	6/4/2015 2:55 PM

210	100	6/4/2015 1:17 PM
211	100	6/4/2015 1:17 PM
212	40	6/4/2015 12:40 PM
213	50	6/4/2015 11:59 AM
214	100	6/4/2015 11:40 AM
215	100	6/4/2015 10:38 AM
216	40	6/4/2015 8:46 AM
217	100%	6/4/2015 8:42 AM
218	50	6/4/2015 8:01 AM
219	100	6/4/2015 7:34 AM
220	100	6/4/2015 7:33 AM
221	90	6/4/2015 7:17 AM
222	30	6/4/2015 7:15 AM
223	50	6/4/2015 5:50 AM
224	85	6/3/2015 10:11 PM
225	100	6/3/2015 9:43 PM
226	60+	6/3/2015 9:37 PM
227	100	6/3/2015 8:23 PM
228	100	6/3/2015 8:17 PM
229	100	6/3/2015 7:56 PM
230	100	6/3/2015 7:45 PM
231	80	6/3/2015 7:35 PM
232	100	6/3/2015 6:48 PM
233	100%	6/3/2015 5:09 PM
234	80	6/3/2015 4:55 PM
235	25	6/3/2015 4:54 PM
236	100	6/3/2015 4:42 PM
237	20	6/3/2015 4:37 PM
238	10	6/3/2015 4:14 PM
239	90	6/3/2015 3:37 PM
240	10	6/3/2015 3:33 PM
241	50	6/3/2015 3:06 PM
242	90	6/3/2015 2:43 PM
243	100	6/3/2015 2:16 PM
244	60	6/3/2015 2:08 PM
245	100	6/3/2015 1:59 PM
246	80	6/3/2015 1:56 PM
247	90	6/3/2015 1:10 PM

248	100 if able	6/3/2015 1:10 PM
249	10	6/3/2015 1:06 PM
250	75%	6/3/2015 12:56 PM
251	80	6/3/2015 12:49 PM
252	50	6/3/2015 12:07 PM
253	100	6/3/2015 11:46 AM
254	100	6/3/2015 11:45 AM
255	50	6/3/2015 11:27 AM
256	50	6/3/2015 11:15 AM
257	80	6/3/2015 11:06 AM
258	100	6/3/2015 11:03 AM
259	100	6/3/2015 10:50 AM
260	50	6/3/2015 10:35 AM
261	100	6/3/2015 10:34 AM
262	50	6/3/2015 10:26 AM
263	20	6/3/2015 10:21 AM
264	100%	6/3/2015 10:18 AM
265	75%	6/3/2015 10:17 AM
266	100	6/3/2015 10:11 AM
267	80	6/3/2015 10:10 AM
268	100%	6/3/2015 10:08 AM
269	75%	6/3/2015 9:59 AM
270	100	6/3/2015 9:48 AM
271	100	6/3/2015 9:31 AM
272	80	6/3/2015 9:28 AM
273	30%	6/3/2015 9:21 AM
274	98%	6/3/2015 9:11 AM
275	100	6/3/2015 8:58 AM
276	100	6/3/2015 8:43 AM
277	100	6/3/2015 8:40 AM
278	100	6/3/2015 8:38 AM
279	75%	6/3/2015 8:19 AM
280	100	6/3/2015 8:14 AM
281	60	6/3/2015 8:11 AM
282	50	6/3/2015 8:10 AM
283	50	6/3/2015 8:09 AM
284	2	6/3/2015 8:01 AM
285	20	6/3/2015 8:01 AM

286	33	6/3/2015 7:53 AM
287	100	6/3/2015 7:50 AM
288	100	6/3/2015 7:45 AM
289	55	6/3/2015 7:33 AM
290	65	6/3/2015 6:39 AM
291	25	6/3/2015 6:31 AM
292	99	6/3/2015 6:23 AM
293	60	6/3/2015 6:05 AM
294	100	6/3/2015 5:49 AM
295	40	6/3/2015 4:09 AM
296	90	6/3/2015 3:47 AM
297	40	6/3/2015 2:45 AM
298	100	6/2/2015 11:59 PM
299	85	6/2/2015 11:11 PM
300	100	6/2/2015 11:02 PM
301	100	6/2/2015 10:51 PM
302	100	6/2/2015 10:43 PM
303	100	6/2/2015 10:37 PM
304	75	6/2/2015 10:24 PM
305	50	6/2/2015 10:18 PM
306	50	6/2/2015 9:59 PM
307	70	6/2/2015 9:59 PM
308	100	6/2/2015 9:52 PM
309	2	6/2/2015 9:49 PM
310	100	6/2/2015 9:39 PM
311	100%	6/2/2015 9:39 PM
312	100	6/2/2015 9:30 PM
313	30	6/2/2015 9:26 PM
314	75	6/2/2015 9:20 PM
315	100	6/2/2015 9:16 PM
316	Your percent 100%	6/2/2015 9:12 PM
317	80	6/2/2015 9:04 PM
318	20	6/2/2015 9:03 PM
319	100	6/2/2015 9:01 PM
320	50	6/2/2015 8:58 PM
321	35	6/2/2015 8:51 PM
322	10%	6/2/2015 8:47 PM
323	90%	6/2/2015 8:45 PM

324	100	6/2/2015 8:43 PM
325	50	6/2/2015 8:42 PM
326	100	6/2/2015 8:38 PM
327	50	6/2/2015 8:36 PM
328	100	6/2/2015 8:19 PM
329	100%	6/2/2015 8:19 PM
330	90	6/2/2015 8:07 PM
331	80%	6/2/2015 8:07 PM
332	60	6/2/2015 8:06 PM
333	0%	6/2/2015 8:03 PM
334	30	6/2/2015 7:59 PM
335	15	6/2/2015 7:57 PM
336	20	6/2/2015 7:56 PM
337	100	6/2/2015 7:47 PM
338	100	6/2/2015 7:43 PM
339	4	6/2/2015 7:42 PM
340	100	6/2/2015 7:31 PM
341	80	6/2/2015 7:25 PM
342	80	6/2/2015 7:13 PM
343	100	6/2/2015 7:10 PM
344	90	6/2/2015 7:07 PM
345	100	6/2/2015 7:07 PM
346	100	6/2/2015 7:04 PM
347	75-100	6/2/2015 7:03 PM
348	100	6/2/2015 7:03 PM
349	100%	6/2/2015 6:56 PM
350	75	6/2/2015 6:42 PM
351	90	6/2/2015 6:42 PM
352	100	6/2/2015 6:30 PM
353	25	6/2/2015 6:28 PM
354	100%	6/2/2015 6:22 PM
355	100	6/2/2015 6:18 PM
356	60	6/2/2015 6:14 PM
357	100%	6/2/2015 6:13 PM
358	100	6/2/2015 6:11 PM
359	100	6/2/2015 6:10 PM
360	100	6/2/2015 6:03 PM
361	35	6/2/2015 5:54 PM

362	50%	6/2/2015 5:51 PM
363	5	6/2/2015 5:49 PM
364	75	6/2/2015 5:47 PM
365	90	6/2/2015 5:47 PM
366	60%	6/2/2015 5:40 PM
367	100	6/2/2015 5:36 PM
368	100	6/2/2015 5:35 PM
369	5	6/2/2015 5:32 PM
370	95	6/2/2015 5:31 PM
371	100%	6/2/2015 5:30 PM
372	100%	6/2/2015 5:29 PM
373	80%	6/2/2015 5:22 PM
374	80	6/2/2015 5:19 PM
375	10	6/2/2015 5:09 PM
376	70	6/2/2015 5:09 PM
377	100	6/2/2015 5:09 PM
378	90	6/2/2015 5:06 PM
379	100	6/2/2015 5:05 PM
380	100	6/2/2015 5:00 PM
381	100	6/2/2015 4:54 PM
382	100	6/2/2015 4:54 PM
383	100	6/2/2015 4:49 PM
384	100	6/2/2015 4:46 PM
385	95	6/2/2015 4:43 PM
386	100	6/2/2015 4:42 PM
387	35	6/2/2015 4:37 PM
388	100	6/2/2015 4:32 PM
389	50%	6/2/2015 4:30 PM
390	90	6/2/2015 4:24 PM
391	100	6/2/2015 4:23 PM
392	100	6/2/2015 4:18 PM
393	5	6/2/2015 4:18 PM
394	70	6/2/2015 4:17 PM
395	70	6/2/2015 4:16 PM
396	85	6/2/2015 4:15 PM
397	100	6/2/2015 4:10 PM
398	100	6/2/2015 4:09 PM
399	25	6/2/2015 4:08 PM

400	90	6/2/2015 4:08 PM
401	100	6/2/2015 4:08 PM
402	100	6/2/2015 4:08 PM
403	40	6/2/2015 4:05 PM
404	100	6/2/2015 4:05 PM
405	100	6/2/2015 4:04 PM
406	95%	6/2/2015 4:02 PM
407	80	6/2/2015 4:02 PM
408	40	6/2/2015 4:01 PM
409	50	6/2/2015 4:01 PM
410	70	6/2/2015 4:00 PM
411	5	6/2/2015 3:59 PM
412	99%	6/2/2015 3:58 PM
413	90	6/2/2015 3:57 PM
414	50% approximately	6/2/2015 3:56 PM
415	70	6/2/2015 3:56 PM
416	50%	6/2/2015 3:56 PM
417	85	6/2/2015 3:55 PM
418	100	6/2/2015 3:54 PM
419	90	6/2/2015 3:54 PM
420	100	6/2/2015 3:52 PM
421	100	6/2/2015 3:50 PM
422	99%	6/2/2015 3:50 PM
423	100	6/2/2015 3:50 PM
424	20	6/2/2015 3:49 PM
425	75	6/2/2015 3:48 PM
426	100	6/2/2015 3:46 PM
427	90	6/2/2015 3:45 PM
428	100	6/2/2015 3:42 PM
429	100	6/2/2015 3:41 PM
430	95	6/2/2015 3:41 PM
431	50	6/2/2015 3:40 PM
432	100	6/2/2015 3:40 PM
433	100	6/2/2015 3:38 PM
434	50	6/2/2015 3:37 PM
435	50	6/2/2015 3:37 PM
436	100%	6/2/2015 3:37 PM
437	25	6/2/2015 3:35 PM

438	90+	6/2/2015 3:35 PM
439	90	6/2/2015 3:35 PM
440	33	6/2/2015 3:34 PM
441	100	6/2/2015 3:31 PM
442	100	6/2/2015 3:31 PM
443	50	6/2/2015 3:31 PM
444	80%	6/2/2015 3:30 PM
445	0	6/2/2015 3:30 PM
446	100	6/2/2015 3:30 PM
447	50	6/2/2015 3:29 PM
448	50	6/2/2015 3:29 PM
449	100	6/2/2015 3:28 PM
450	100	6/2/2015 3:28 PM
451	70	6/2/2015 3:27 PM
452	50	6/2/2015 3:27 PM
453	90	6/2/2015 3:27 PM
454	100	6/2/2015 3:26 PM
455	25	6/2/2015 3:25 PM
456	100	6/2/2015 3:25 PM
457	20	6/2/2015 3:24 PM
458	60	6/2/2015 3:23 PM
459	80	6/2/2015 3:23 PM
460	90	6/2/2015 3:23 PM
461	100	6/2/2015 3:23 PM
462	30	6/2/2015 3:23 PM
463	75	6/2/2015 3:23 PM
464	20	6/2/2015 3:22 PM
465	50	6/2/2015 3:21 PM

#	New patients w/ history of asthma to assess initial asthma control:	Date
1	45	6/19/2015 4:48 PM
2	60	6/19/2015 12:50 PM
3	50	6/19/2015 12:32 PM
4	100	6/18/2015 5:24 PM
5	30	6/18/2015 4:49 PM
6	10	6/18/2015 12:03 PM
7	5	6/18/2015 9:56 AM
8	100	6/17/2015 4:53 PM
9	100	6/17/2015 4:01 PM

10	10	6/17/2015 2:37 PM
11	20	6/17/2015 10:54 AM
12	90	6/17/2015 9:43 AM
13	25	6/16/2015 9:38 PM
14	10	6/16/2015 9:36 PM
15	5	6/16/2015 9:00 PM
16	10	6/16/2015 8:33 PM
17	100%	6/16/2015 8:00 PM
18	100 %	6/16/2015 7:01 PM
19	25	6/16/2015 6:00 PM
20	50	6/16/2015 5:43 PM
21	20	6/16/2015 4:09 PM
22	90	6/16/2015 3:37 PM
23	100	6/16/2015 2:42 PM
24	40	6/16/2015 2:24 PM
25	80	6/16/2015 2:10 PM
26	100	6/16/2015 2:00 PM
27	30	6/16/2015 1:36 PM
28	25	6/16/2015 1:22 PM
29	90	6/16/2015 1:01 PM
30	75	6/16/2015 12:55 PM
31	0	6/16/2015 12:42 PM
32	100	6/16/2015 12:33 PM
33	30	6/16/2015 12:22 PM
34	90	6/16/2015 11:55 AM
35	0	6/16/2015 11:36 AM
36	90	6/16/2015 11:33 AM
37	25	6/16/2015 11:33 AM
38	20	6/16/2015 11:18 AM
39	100	6/16/2015 11:00 AM
40	90	6/16/2015 10:58 AM
41	1	6/16/2015 10:38 AM
42	85	6/16/2015 10:12 AM
43	40	6/16/2015 10:05 AM
44	80%	6/16/2015 10:05 AM
45	100	6/16/2015 9:56 AM
46	95	6/16/2015 9:30 AM
47	85	6/16/2015 9:29 AM

48	15	6/16/2015 9:23 AM
49	70	6/16/2015 9:15 AM
50	100	6/16/2015 9:14 AM
51	0	6/16/2015 9:12 AM
52	0	6/16/2015 9:12 AM
53	100	6/16/2015 9:10 AM
54	100	6/16/2015 9:10 AM
55	80	6/16/2015 9:09 AM
56	50	6/16/2015 9:09 AM
57	50	6/16/2015 9:06 AM
58	90	6/16/2015 9:05 AM
59	100%	6/15/2015 8:48 PM
60	100	6/14/2015 9:41 PM
61	5	6/14/2015 8:35 PM
62	80%	6/14/2015 7:25 PM
63	30	6/14/2015 4:52 PM
64	100	6/14/2015 1:43 AM
65	40	6/13/2015 8:39 PM
66	50%	6/12/2015 12:27 PM
67	38%	6/12/2015 10:34 AM
68	40	6/12/2015 7:51 AM
69	10	6/12/2015 6:19 AM
70	-	6/11/2015 11:34 AM
71	10	6/11/2015 9:23 AM
72	80%	6/11/2015 12:00 AM
73	80	6/10/2015 5:29 PM
74	30	6/10/2015 3:17 PM
75	15%	6/10/2015 2:53 PM
76	5	6/10/2015 2:43 PM
77	15	6/10/2015 2:19 PM
78	40	6/10/2015 2:00 PM
79	50	6/10/2015 1:29 PM
80	10	6/10/2015 1:19 PM
81	50	6/10/2015 1:13 PM
82	60	6/10/2015 12:50 PM
83	15	6/10/2015 11:58 AM
84	100	6/10/2015 11:41 AM
85	80	6/10/2015 11:32 AM

86	50	6/10/2015 11:26 AM
87	25	6/10/2015 10:26 AM
88	100	6/10/2015 9:48 AM
89	100	6/10/2015 9:14 AM
90	80	6/10/2015 8:58 AM
91	20%	6/10/2015 8:37 AM
92	100	6/10/2015 8:08 AM
93	8	6/10/2015 7:53 AM
94	70	6/10/2015 6:16 AM
95	0	6/10/2015 6:04 AM
96	0	6/10/2015 5:55 AM
97	100%	6/10/2015 1:54 AM
98	75	6/10/2015 12:39 AM
99	80	6/9/2015 10:57 PM
100	100%	6/9/2015 10:28 PM
101	100	6/9/2015 9:56 PM
102	40	6/9/2015 9:26 PM
103	10	6/9/2015 8:46 PM
104	100	6/9/2015 8:03 PM
105	10	6/9/2015 7:51 PM
106	100	6/9/2015 7:48 PM
107	60	6/9/2015 7:48 PM
108	100	6/9/2015 7:17 PM
109	100	6/9/2015 6:57 PM
110	5	6/9/2015 6:55 PM
111	50	6/9/2015 6:27 PM
112	40	6/9/2015 6:13 PM
113	30	6/9/2015 6:00 PM
114	100	6/9/2015 5:25 PM
115	100	6/9/2015 5:23 PM
116	100	6/9/2015 5:21 PM
117	100	6/9/2015 5:20 PM
118	100	6/9/2015 5:08 PM
119	0	6/9/2015 5:03 PM
120	20	6/9/2015 5:00 PM
121	10	6/9/2015 4:55 PM
122	80	6/9/2015 4:53 PM
123	100	6/9/2015 4:47 PM

124	50	6/9/2015 4:41 PM
125	50	6/9/2015 4:24 PM
126	95	6/9/2015 4:06 PM
127	50%	6/9/2015 4:03 PM
128	100%	6/9/2015 3:56 PM
129	25	6/9/2015 3:44 PM
130	30	6/9/2015 3:41 PM
131	80-90%	6/9/2015 3:38 PM
132	50	6/9/2015 3:32 PM
133	100	6/9/2015 3:27 PM
134	100	6/9/2015 3:25 PM
135	100	6/9/2015 3:22 PM
136	100	6/9/2015 3:16 PM
137	25	6/9/2015 3:10 PM
138	50	6/9/2015 3:08 PM
139	50	6/9/2015 2:55 PM
140	0	6/9/2015 2:50 PM
141	20	6/9/2015 2:49 PM
142	70%	6/9/2015 2:39 PM
143	10	6/9/2015 2:36 PM
144	100%	6/9/2015 2:32 PM
145	0	6/9/2015 2:31 PM
146	90	6/9/2015 2:29 PM
147	100	6/9/2015 2:25 PM
148	40	6/9/2015 2:23 PM
149	70	6/9/2015 2:22 PM
150	5	6/9/2015 2:15 PM
151	100	6/9/2015 1:58 PM
152	20	6/9/2015 1:58 PM
153	50	6/9/2015 1:44 PM
154	20	6/9/2015 1:40 PM
155	50	6/9/2015 1:29 PM
156	100	6/9/2015 1:27 PM
157	100	6/9/2015 1:27 PM
158	100	6/9/2015 1:19 PM
159	100	6/9/2015 1:13 PM
160	75	6/9/2015 1:12 PM
161	90%	6/9/2015 1:12 PM

162	10	6/9/2015 1:11 PM
163	45	6/9/2015 1:11 PM
164	0	6/9/2015 1:10 PM
165	99	6/9/2015 1:07 PM
166	15	6/9/2015 7:41 AM
167	10	6/9/2015 7:24 AM
168	20	6/9/2015 4:56 AM
169	100	6/9/2015 1:59 AM
170	100	6/8/2015 8:34 PM
171	100	6/8/2015 1:36 PM
172	30	6/8/2015 12:10 PM
173	30%	6/8/2015 11:34 AM
174	80	6/8/2015 7:42 AM
175	100	6/7/2015 9:04 PM
176	100	6/7/2015 4:35 PM
177	100	6/7/2015 3:19 PM
178	100	6/7/2015 2:48 PM
179	0	6/7/2015 2:37 PM
180	40	6/7/2015 1:33 PM
181	100	6/7/2015 12:01 PM
182	100	6/6/2015 9:35 PM
183	100	6/6/2015 12:32 PM
184	100%	6/6/2015 12:16 AM
185	10	6/5/2015 8:41 PM
186	0	6/5/2015 3:32 PM
187	100	6/5/2015 2:51 PM
188	80	6/5/2015 2:26 PM
189	100	6/5/2015 1:51 PM
190	90%	6/5/2015 12:29 PM
191	100	6/5/2015 10:34 AM
192	100%	6/5/2015 4:40 AM
193	50	6/4/2015 9:32 PM
194	90	6/4/2015 6:33 PM
195	20	6/4/2015 5:46 PM
196	90	6/4/2015 4:30 PM
197	100%	6/4/2015 4:28 PM
198	99	6/4/2015 3:21 PM
199	50	6/4/2015 3:15 PM

200	90	6/4/2015 2:55 PM
201	95	6/4/2015 1:17 PM
202	100	6/4/2015 1:17 PM
203	40	6/4/2015 12:40 PM
204	75	6/4/2015 11:59 AM
205	50	6/4/2015 11:40 AM
206	100	6/4/2015 10:38 AM
207	50	6/4/2015 8:46 AM
208	100%	6/4/2015 8:42 AM
209	5	6/4/2015 8:01 AM
210	100	6/4/2015 7:34 AM
211	75	6/4/2015 7:33 AM
212	95	6/4/2015 7:17 AM
213	20	6/4/2015 7:15 AM
214	50	6/4/2015 5:50 AM
215	85	6/3/2015 10:11 PM
216	100	6/3/2015 9:43 PM
217	60+	6/3/2015 9:37 PM
218	50	6/3/2015 8:23 PM
219	10	6/3/2015 8:17 PM
220	50	6/3/2015 7:56 PM
221	50	6/3/2015 7:45 PM
222	60	6/3/2015 7:35 PM
223	70	6/3/2015 6:48 PM
224	0%	6/3/2015 5:09 PM
225	50	6/3/2015 4:55 PM
226	25	6/3/2015 4:54 PM
227	90	6/3/2015 4:42 PM
228	4	6/3/2015 4:37 PM
229	10	6/3/2015 4:14 PM
230	95	6/3/2015 3:37 PM
231	25	6/3/2015 3:06 PM
232	10	6/3/2015 2:43 PM
233	100	6/3/2015 2:16 PM
234	60	6/3/2015 2:08 PM
235	100	6/3/2015 1:59 PM
236	75	6/3/2015 1:56 PM
237	90	6/3/2015 1:10 PM

238	100 if able	6/3/2015 1:10 PM
239	10	6/3/2015 1:06 PM
240	90%	6/3/2015 12:56 PM
241	50	6/3/2015 12:49 PM
242	25	6/3/2015 12:07 PM
243	100	6/3/2015 11:46 AM
244	100	6/3/2015 11:45 AM
245	50	6/3/2015 11:27 AM
246	25	6/3/2015 11:15 AM
247	15	6/3/2015 11:06 AM
248	90	6/3/2015 11:03 AM
249	100	6/3/2015 10:50 AM
250	20	6/3/2015 10:35 AM
251	100	6/3/2015 10:34 AM
252	0	6/3/2015 10:26 AM
253	0	6/3/2015 10:18 AM
254	90%	6/3/2015 10:17 AM
255	75	6/3/2015 10:11 AM
256	80	6/3/2015 10:10 AM
257	100%	6/3/2015 10:08 AM
258	100%	6/3/2015 9:59 AM
259	100	6/3/2015 9:48 AM
260	99	6/3/2015 9:31 AM
261	0	6/3/2015 9:28 AM
262	20%	6/3/2015 9:21 AM
263	98%	6/3/2015 9:11 AM
264	100	6/3/2015 8:58 AM
265	80	6/3/2015 8:43 AM
266	50	6/3/2015 8:40 AM
267	100	6/3/2015 8:38 AM
268	75%	6/3/2015 8:19 AM
269	75	6/3/2015 8:14 AM
270	5	6/3/2015 8:13 AM
271	50	6/3/2015 8:11 AM
272	0	6/3/2015 8:10 AM
273	15	6/3/2015 8:09 AM
274	2	6/3/2015 8:01 AM
275	20	6/3/2015 8:01 AM

276	33	6/3/2015 7:53 AM
277	100	6/3/2015 7:50 AM
278	100	6/3/2015 7:45 AM
279	30	6/3/2015 7:33 AM
280	0	6/3/2015 6:31 AM
281	50	6/3/2015 6:23 AM
282	30	6/3/2015 6:05 AM
283	100	6/3/2015 5:49 AM
284	60	6/3/2015 4:09 AM
285	60	6/3/2015 3:47 AM
286	100	6/3/2015 2:45 AM
287	85	6/2/2015 11:59 PM
288	5	6/2/2015 11:11 PM
289	0	6/2/2015 10:51 PM
290	100	6/2/2015 10:43 PM
291	80	6/2/2015 10:37 PM
292	50	6/2/2015 10:24 PM
293	0	6/2/2015 10:18 PM
294	50	6/2/2015 9:59 PM
295	60	6/2/2015 9:59 PM
296	100	6/2/2015 9:52 PM
297	0	6/2/2015 9:49 PM
298	95	6/2/2015 9:39 PM
299	95%	6/2/2015 9:39 PM
300	75	6/2/2015 9:30 PM
301	30	6/2/2015 9:26 PM
302	75	6/2/2015 9:20 PM
303	10-0	6/2/2015 9:16 PM
304	Your percent 100%	6/2/2015 9:12 PM
305	60	6/2/2015 9:04 PM
306	20	6/2/2015 9:03 PM
307	100	6/2/2015 9:01 PM
308	30	6/2/2015 8:58 PM
309	55	6/2/2015 8:51 PM
310	75%	6/2/2015 8:47 PM
311	0	6/2/2015 8:45 PM
312	100	6/2/2015 8:43 PM
313	20	6/2/2015 8:42 PM

314	50	6/2/2015 8:38 PM
315	0	6/2/2015 8:36 PM
316	100	6/2/2015 8:19 PM
317	50%	6/2/2015 8:19 PM
318	0	6/2/2015 8:07 PM
319	50%	6/2/2015 8:07 PM
320	40	6/2/2015 8:06 PM
321	10%	6/2/2015 8:03 PM
322	30	6/2/2015 7:59 PM
323	0	6/2/2015 7:57 PM
324	20	6/2/2015 7:56 PM
325	100	6/2/2015 7:47 PM
326	5	6/2/2015 7:42 PM
327	100	6/2/2015 7:31 PM
328	20	6/2/2015 7:25 PM
329	80	6/2/2015 7:13 PM
330	100	6/2/2015 7:10 PM
331	95	6/2/2015 7:07 PM
332	100	6/2/2015 7:07 PM
333	100	6/2/2015 7:04 PM
334	O	6/2/2015 7:03 PM
335	100%	6/2/2015 6:56 PM
336	75	6/2/2015 6:42 PM
337	50	6/2/2015 6:42 PM
338	100	6/2/2015 6:30 PM
339	25	6/2/2015 6:28 PM
340	100%	6/2/2015 6:22 PM
341	100	6/2/2015 6:18 PM
342	5	6/2/2015 6:14 PM
343	50%	6/2/2015 6:13 PM
344	75	6/2/2015 6:11 PM
345	100	6/2/2015 6:10 PM
346	100	6/2/2015 6:03 PM
347	60	6/2/2015 5:54 PM
348	5	6/2/2015 5:49 PM
349	50	6/2/2015 5:47 PM
350	100	6/2/2015 5:47 PM
351	20%	6/2/2015 5:40 PM

352	85	6/2/2015 5:36 PM
353	80	6/2/2015 5:35 PM
354	5	6/2/2015 5:32 PM
355	95	6/2/2015 5:31 PM
356	100%	6/2/2015 5:30 PM
357	100%	6/2/2015 5:29 PM
358	80	6/2/2015 5:19 PM
359	10	6/2/2015 5:09 PM
360	50	6/2/2015 5:09 PM
361	0	6/2/2015 5:09 PM
362	75	6/2/2015 5:06 PM
363	20	6/2/2015 5:05 PM
364	100	6/2/2015 5:00 PM
365	100	6/2/2015 4:54 PM
366	100	6/2/2015 4:54 PM
367	100	6/2/2015 4:49 PM
368	100	6/2/2015 4:46 PM
369	95	6/2/2015 4:43 PM
370	75	6/2/2015 4:42 PM
371	65	6/2/2015 4:37 PM
372	100	6/2/2015 4:32 PM
373	10%	6/2/2015 4:30 PM
374	90	6/2/2015 4:24 PM
375	100	6/2/2015 4:23 PM
376	100	6/2/2015 4:18 PM
377	0	6/2/2015 4:18 PM
378	26	6/2/2015 4:17 PM
379	10	6/2/2015 4:16 PM
380	50	6/2/2015 4:16 PM
381	0	6/2/2015 4:10 PM
382	100	6/2/2015 4:09 PM
383	25	6/2/2015 4:08 PM
384	50	6/2/2015 4:08 PM
385	100	6/2/2015 4:08 PM
386	100	6/2/2015 4:08 PM
387	55	6/2/2015 4:05 PM
388	0	6/2/2015 4:05 PM
389	100	6/2/2015 4:04 PM

390	95%	6/2/2015 4:02 PM
391	50	6/2/2015 4:02 PM
392	40	6/2/2015 4:01 PM
393	50	6/2/2015 4:01 PM
394	15	6/2/2015 4:00 PM
395	5	6/2/2015 3:59 PM
396	95%	6/2/2015 3:58 PM
397	75	6/2/2015 3:57 PM
398	5	6/2/2015 3:56 PM
399	50%	6/2/2015 3:56 PM
400	85	6/2/2015 3:55 PM
401	100	6/2/2015 3:54 PM
402	90	6/2/2015 3:54 PM
403	100	6/2/2015 3:52 PM
404	75	6/2/2015 3:50 PM
405	99%	6/2/2015 3:50 PM
406	100	6/2/2015 3:50 PM
407	30	6/2/2015 3:49 PM
408	25	6/2/2015 3:48 PM
409	100	6/2/2015 3:46 PM
410	5	6/2/2015 3:45 PM
411	100	6/2/2015 3:42 PM
412	100	6/2/2015 3:41 PM
413	25	6/2/2015 3:41 PM
414	50	6/2/2015 3:40 PM
415	0	6/2/2015 3:40 PM
416	100	6/2/2015 3:38 PM
417	50	6/2/2015 3:37 PM
418	25	6/2/2015 3:37 PM
419	100%	6/2/2015 3:37 PM
420	40	6/2/2015 3:35 PM
421	90+	6/2/2015 3:35 PM
422	90	6/2/2015 3:35 PM
423	33	6/2/2015 3:34 PM
424	0	6/2/2015 3:31 PM
425	100	6/2/2015 3:31 PM
426	50	6/2/2015 3:31 PM
427	50%	6/2/2015 3:30 PM

428	0	6/2/2015 3:30 PM
429	100	6/2/2015 3:30 PM
430	50	6/2/2015 3:29 PM
431	50	6/2/2015 3:29 PM
432	100	6/2/2015 3:28 PM
433	100	6/2/2015 3:28 PM
434	40	6/2/2015 3:27 PM
435	50	6/2/2015 3:27 PM
436	90	6/2/2015 3:27 PM
437	100	6/2/2015 3:26 PM
438	100	6/2/2015 3:25 PM
439	5	6/2/2015 3:24 PM
440	60	6/2/2015 3:23 PM
441	80	6/2/2015 3:23 PM
442	90	6/2/2015 3:23 PM
443	100	6/2/2015 3:23 PM
444	10	6/2/2015 3:23 PM
445	80	6/2/2015 3:23 PM
446	10	6/2/2015 3:22 PM
447	30	6/2/2015 3:21 PM

#	Follow-up patients w/ asthma to assess follow-up asthma control:	Date
1	10	6/19/2015 4:48 PM
2	20	6/19/2015 12:50 PM
3	10	6/19/2015 12:32 PM
4	100	6/18/2015 5:24 PM
5	30	6/18/2015 4:49 PM
6	5	6/18/2015 12:03 PM
7	10	6/18/2015 9:56 AM
8	50	6/17/2015 4:53 PM
9	75	6/17/2015 4:01 PM
10	0	6/17/2015 2:37 PM
11	10	6/17/2015 10:54 AM
12	75	6/17/2015 9:43 AM
13	25	6/16/2015 9:38 PM
14	0	6/16/2015 9:36 PM
15	5	6/16/2015 9:00 PM
16	10	6/16/2015 8:33 PM
17	100%	6/16/2015 8:00 PM

18	100 %	6/16/2015 7:01 PM
19	0	6/16/2015 6:00 PM
20	10	6/16/2015 5:43 PM
21	60	6/16/2015 4:09 PM
22	50	6/16/2015 3:37 PM
23	100	6/16/2015 2:42 PM
24	30	6/16/2015 2:24 PM
25	0	6/16/2015 2:10 PM
26	50	6/16/2015 2:00 PM
27	40	6/16/2015 1:36 PM
28	5	6/16/2015 1:22 PM
29	Less than 5	6/16/2015 1:01 PM
30	20	6/16/2015 12:55 PM
31	0	6/16/2015 12:42 PM
32	90	6/16/2015 12:33 PM
33	10	6/16/2015 12:22 PM
34	75	6/16/2015 11:55 AM
35	25	6/16/2015 11:36 AM
36	40	6/16/2015 11:33 AM
37	25	6/16/2015 11:33 AM
38	20	6/16/2015 11:18 AM
39	50	6/16/2015 11:00 AM
40	20	6/16/2015 10:58 AM
41	1	6/16/2015 10:38 AM
42	85	6/16/2015 10:12 AM
43	5	6/16/2015 10:05 AM
44	80%	6/16/2015 10:05 AM
45	70	6/16/2015 9:56 AM
46	0	6/16/2015 9:30 AM
47	10	6/16/2015 9:29 AM
48	20	6/16/2015 9:23 AM
49	30	6/16/2015 9:15 AM
50	>50	6/16/2015 9:14 AM
51	0	6/16/2015 9:12 AM
52	0	6/16/2015 9:12 AM
53	10	6/16/2015 9:10 AM
54	15	6/16/2015 9:10 AM
55	100	6/16/2015 9:10 AM

56	80	6/16/2015 9:09 AM
57	10	6/16/2015 9:09 AM
58	25	6/16/2015 9:06 AM
59	75	6/16/2015 9:05 AM
60	100%	6/15/2015 8:48 PM
61	60	6/14/2015 9:41 PM
62	5	6/14/2015 8:35 PM
63	50%	6/14/2015 7:25 PM
64	5	6/14/2015 4:52 PM
65	20	6/14/2015 1:43 AM
66	20	6/13/2015 8:39 PM
67	30	6/12/2015 9:10 PM
68	0	6/12/2015 10:34 AM
69	10	6/12/2015 7:51 AM
70	50	6/12/2015 6:19 AM
71	90	6/11/2015 11:34 AM
72	30	6/11/2015 9:23 AM
73	50%	6/11/2015 12:00 AM
74	50	6/10/2015 5:29 PM
75	30	6/10/2015 3:17 PM
76	15%	6/10/2015 2:53 PM
77	5	6/10/2015 2:43 PM
78	20	6/10/2015 2:19 PM
79	15	6/10/2015 2:00 PM
80	10	6/10/2015 1:29 PM
81	10	6/10/2015 1:19 PM
82	5	6/10/2015 1:04 PM
83	40	6/10/2015 12:50 PM
84	50	6/10/2015 11:41 AM
85	60	6/10/2015 11:32 AM
86	25	6/10/2015 11:26 AM
87	50	6/10/2015 11:25 AM
88	50	6/10/2015 10:26 AM
89	100	6/10/2015 9:48 AM
90	80	6/10/2015 9:14 AM
91	0	6/10/2015 8:58 AM
92	5%	6/10/2015 8:37 AM
93	30	6/10/2015 8:08 AM

94	5	6/10/2015 7:53 AM
95	25	6/10/2015 6:16 AM
96	0	6/10/2015 6:04 AM
97	0	6/10/2015 5:55 AM
98	50%	6/10/2015 1:54 AM
99	25	6/10/2015 12:39 AM
100	80	6/9/2015 10:57 PM
101	10%	6/9/2015 10:28 PM
102	80	6/9/2015 9:56 PM
103	10	6/9/2015 9:26 PM
104	10	6/9/2015 8:46 PM
105	100	6/9/2015 8:03 PM
106	0	6/9/2015 7:51 PM
107	100	6/9/2015 7:48 PM
108	6-	6/9/2015 7:48 PM
109	50	6/9/2015 7:17 PM
110	50	6/9/2015 6:57 PM
111	5	6/9/2015 6:55 PM
112	10	6/9/2015 6:27 PM
113	1	6/9/2015 6:13 PM
114	30	6/9/2015 6:00 PM
115	20	6/9/2015 5:25 PM
116	15	6/9/2015 5:23 PM
117	50	6/9/2015 5:21 PM
118	100	6/9/2015 5:20 PM
119	100	6/9/2015 5:08 PM
120	0	6/9/2015 5:03 PM
121	0	6/9/2015 5:00 PM
122	0	6/9/2015 4:55 PM
123	Every 6 months	6/9/2015 4:47 PM
124	50	6/9/2015 4:41 PM
125	25	6/9/2015 4:24 PM
126	20	6/9/2015 4:06 PM
127	5%	6/9/2015 4:03 PM
128	100%	6/9/2015 3:56 PM
129	50	6/9/2015 3:44 PM
130	0	6/9/2015 3:41 PM
131	50%	6/9/2015 3:38 PM

132	5	6/9/2015 3:32 PM
133	100	6/9/2015 3:27 PM
134	100	6/9/2015 3:25 PM
135	50	6/9/2015 3:16 PM
136	0	6/9/2015 3:10 PM
137	10	6/9/2015 3:08 PM
138	50	6/9/2015 2:55 PM
139	0	6/9/2015 2:50 PM
140	10	6/9/2015 2:49 PM
141	50%	6/9/2015 2:39 PM
142	15	6/9/2015 2:36 PM
143	50%	6/9/2015 2:32 PM
144	0	6/9/2015 2:31 PM
145	90	6/9/2015 2:29 PM
146	100	6/9/2015 2:25 PM
147	20	6/9/2015 2:23 PM
148	30	6/9/2015 2:22 PM
149	5	6/9/2015 2:15 PM
150	10	6/9/2015 1:58 PM
151	30	6/9/2015 1:58 PM
152	10	6/9/2015 1:44 PM
153	10	6/9/2015 1:40 PM
154	10	6/9/2015 1:29 PM
155	25	6/9/2015 1:27 PM
156	100	6/9/2015 1:27 PM
157	0	6/9/2015 1:19 PM
158	40	6/9/2015 1:13 PM
159	20	6/9/2015 1:12 PM
160	50%	6/9/2015 1:12 PM
161	0	6/9/2015 1:11 PM
162	10	6/9/2015 1:11 PM
163	1	6/9/2015 1:10 PM
164	80	6/9/2015 1:07 PM
165	15	6/9/2015 7:41 AM
166	20	6/9/2015 7:24 AM
167	20	6/9/2015 4:56 AM
168	50	6/9/2015 1:59 AM
169	100	6/8/2015 8:34 PM

170	80	6/8/2015 1:36 PM
171	30	6/8/2015 12:10 PM
172	5%	6/8/2015 11:34 AM
173	10	6/8/2015 7:42 AM
174	30	6/7/2015 9:04 PM
175	100	6/7/2015 4:35 PM
176	20	6/7/2015 3:19 PM
177	100	6/7/2015 2:48 PM
178	0	6/7/2015 2:37 PM
179	5	6/7/2015 1:33 PM
180	50	6/7/2015 12:01 PM
181	50	6/6/2015 9:35 PM
182	0	6/6/2015 12:32 PM
183	25%	6/6/2015 12:16 AM
184	5	6/5/2015 8:41 PM
185	0	6/5/2015 3:32 PM
186	75	6/5/2015 2:51 PM
187	10	6/5/2015 2:26 PM
188	100	6/5/2015 1:51 PM
189	35%	6/5/2015 12:29 PM
190	75	6/5/2015 10:34 AM
191	0	6/4/2015 9:32 PM
192	10	6/4/2015 6:33 PM
193	10	6/4/2015 5:46 PM
194	80	6/4/2015 4:30 PM
195	80%	6/4/2015 4:28 PM
196	99	6/4/2015 3:21 PM
197	80	6/4/2015 2:55 PM
198	5	6/4/2015 1:17 PM
199	100	6/4/2015 1:17 PM
200	20	6/4/2015 12:40 PM
201	10	6/4/2015 11:59 AM
202	20	6/4/2015 11:40 AM
203	100	6/4/2015 10:38 AM
204	10	6/4/2015 8:46 AM
205	10%	6/4/2015 8:42 AM
206	0	6/4/2015 8:01 AM
207	25	6/4/2015 7:34 AM

208	10	6/4/2015 7:33 AM
209	5	6/4/2015 7:17 AM
210	50	6/4/2015 7:15 AM
211	15	6/4/2015 5:50 AM
212	20	6/3/2015 10:11 PM
213	100	6/3/2015 9:43 PM
214	60+	6/3/2015 9:37 PM
215	0	6/3/2015 8:23 PM
216	10	6/3/2015 8:17 PM
217	10	6/3/2015 7:56 PM
218	0	6/3/2015 7:45 PM
219	0	6/3/2015 7:35 PM
220	50	6/3/2015 6:48 PM
221	0%	6/3/2015 5:09 PM
222	25	6/3/2015 4:54 PM
223	75	6/3/2015 4:42 PM
224	1	6/3/2015 4:37 PM
225	5	6/3/2015 4:14 PM
226	25	6/3/2015 3:37 PM
227	10	6/3/2015 3:06 PM
228	10	6/3/2015 2:43 PM
229	100	6/3/2015 2:16 PM
230	20	6/3/2015 2:08 PM
231	100	6/3/2015 1:59 PM
232	5	6/3/2015 1:56 PM
233	90	6/3/2015 1:10 PM
234	100 if able	6/3/2015 1:10 PM
235	0	6/3/2015 1:06 PM
236	0	6/3/2015 12:56 PM
237	0	6/3/2015 12:49 PM
238	10	6/3/2015 12:07 PM
239	60	6/3/2015 11:46 AM
240	98	6/3/2015 11:45 AM
241	0	6/3/2015 11:27 AM
242	25	6/3/2015 11:15 AM
243	0	6/3/2015 11:06 AM
244	5	6/3/2015 11:03 AM
245	100	6/3/2015 10:50 AM

246	30	6/3/2015 10:35 AM
247	25	6/3/2015 10:34 AM
248	0	6/3/2015 10:26 AM
249	30	6/3/2015 10:21 AM
250	0	6/3/2015 10:18 AM
251	75%	6/3/2015 10:17 AM
252	75	6/3/2015 10:11 AM
253	10	6/3/2015 10:10 AM
254	50%	6/3/2015 10:08 AM
255	10%	6/3/2015 9:59 AM
256	20	6/3/2015 9:48 AM
257	30	6/3/2015 9:31 AM
258	0	6/3/2015 9:28 AM
259	50%	6/3/2015 9:21 AM
260	98%	6/3/2015 9:11 AM
261	5	6/3/2015 8:58 AM
262	20	6/3/2015 8:43 AM
263	25	6/3/2015 8:40 AM
264	60	6/3/2015 8:38 AM
265	50%	6/3/2015 8:19 AM
266	25	6/3/2015 8:14 AM
267	10	6/3/2015 8:11 AM
268	0	6/3/2015 8:10 AM
269	0-5	6/3/2015 8:09 AM
270	2	6/3/2015 8:01 AM
271	20	6/3/2015 8:01 AM
272	34	6/3/2015 7:53 AM
273	5	6/3/2015 7:50 AM
274	100	6/3/2015 7:45 AM
275	5	6/3/2015 7:33 AM
276	20	6/3/2015 6:39 AM
277	0	6/3/2015 6:31 AM
278	0	6/3/2015 6:23 AM
279	0	6/3/2015 6:05 AM
280	50	6/3/2015 5:49 AM
281	25	6/3/2015 3:47 AM
282	100	6/3/2015 2:45 AM
283	80	6/2/2015 11:59 PM

284	5	6/2/2015 11:11 PM
285	0	6/2/2015 10:51 PM
286	0	6/2/2015 10:43 PM
287	100	6/2/2015 10:37 PM
288	10	6/2/2015 10:24 PM
289	10	6/2/2015 10:18 PM
290	30	6/2/2015 9:59 PM
291	10	6/2/2015 9:52 PM
292	2	6/2/2015 9:49 PM
293	30	6/2/2015 9:39 PM
294	100%	6/2/2015 9:39 PM
295	25	6/2/2015 9:30 PM
296	40	6/2/2015 9:26 PM
297	10	6/2/2015 9:20 PM
298	60	6/2/2015 9:16 PM
299	Hundred percent	6/2/2015 9:12 PM
300	10	6/2/2015 9:04 PM
301	40	6/2/2015 9:03 PM
302	50	6/2/2015 9:01 PM
303	15	6/2/2015 8:58 PM
304	10	6/2/2015 8:51 PM
305	15%	6/2/2015 8:47 PM
306	0	6/2/2015 8:45 PM
307	25	6/2/2015 8:43 PM
308	0	6/2/2015 8:42 PM
309	0	6/2/2015 8:38 PM
310	0	6/2/2015 8:36 PM
311	100	6/2/2015 8:19 PM
312	10%	6/2/2015 8:19 PM
313	0	6/2/2015 8:07 PM
314	25%	6/2/2015 8:07 PM
315	25%	6/2/2015 8:03 PM
316	10	6/2/2015 7:59 PM
317	5	6/2/2015 7:57 PM
318	0	6/2/2015 7:56 PM
319	0	6/2/2015 7:47 PM
320	5	6/2/2015 7:42 PM
321	100	6/2/2015 7:31 PM

322	15	6/2/2015 7:25 PM
323	100	6/2/2015 7:10 PM
324	50	6/2/2015 7:07 PM
325	0	6/2/2015 7:07 PM
326	100	6/2/2015 7:04 PM
327	O	6/2/2015 7:03 PM
328	10%	6/2/2015 6:56 PM
329	75	6/2/2015 6:42 PM
330	25	6/2/2015 6:42 PM
331	100	6/2/2015 6:30 PM
332	25	6/2/2015 6:28 PM
333	100%	6/2/2015 6:22 PM
334	100	6/2/2015 6:18 PM
335	5	6/2/2015 6:14 PM
336	25%	6/2/2015 6:13 PM
337	5	6/2/2015 6:11 PM
338	100	6/2/2015 6:10 PM
339	10	6/2/2015 6:03 PM
340	5	6/2/2015 5:54 PM
341	0	6/2/2015 5:49 PM
342	5	6/2/2015 5:47 PM
343	50	6/2/2015 5:47 PM
344	18%	6/2/2015 5:40 PM
345	80	6/2/2015 5:36 PM
346	90	6/2/2015 5:35 PM
347	0	6/2/2015 5:32 PM
348	5	6/2/2015 5:31 PM
349	50%	6/2/2015 5:30 PM
350	70	6/2/2015 5:19 PM
351	5	6/2/2015 5:09 PM
352	0	6/2/2015 5:09 PM
353	100	6/2/2015 5:06 PM
354	5	6/2/2015 5:05 PM
355	1	6/2/2015 5:00 PM
356	100	6/2/2015 4:54 PM
357	70	6/2/2015 4:49 PM
358	100	6/2/2015 4:46 PM
359	10	6/2/2015 4:43 PM

360	75	6/2/2015 4:42 PM
361	100	6/2/2015 4:32 PM
362	1%	6/2/2015 4:30 PM
363	5	6/2/2015 4:24 PM
364	10	6/2/2015 4:23 PM
365	100	6/2/2015 4:18 PM
366	0	6/2/2015 4:18 PM
367	2	6/2/2015 4:17 PM
368	90	6/2/2015 4:16 PM
369	25	6/2/2015 4:16 PM
370	0	6/2/2015 4:10 PM
371	5	6/2/2015 4:09 PM
372	10	6/2/2015 4:08 PM
373	20	6/2/2015 4:08 PM
374	100	6/2/2015 4:08 PM
375	5	6/2/2015 4:08 PM
376	5	6/2/2015 4:05 PM
377	0	6/2/2015 4:05 PM
378	5	6/2/2015 4:04 PM
379	50%	6/2/2015 4:02 PM
380	30	6/2/2015 4:02 PM
381	20	6/2/2015 4:01 PM
382	0	6/2/2015 4:01 PM
383	5	6/2/2015 4:00 PM
384	5	6/2/2015 3:59 PM
385	25%	6/2/2015 3:58 PM
386	40	6/2/2015 3:57 PM
387	25	6/2/2015 3:56 PM
388	50%	6/2/2015 3:56 PM
389	40	6/2/2015 3:55 PM
390	50	6/2/2015 3:54 PM
391	10	6/2/2015 3:54 PM
392	10	6/2/2015 3:52 PM
393	5%	6/2/2015 3:50 PM
394	100	6/2/2015 3:50 PM
395	5	6/2/2015 3:49 PM
396	5	6/2/2015 3:48 PM
397	100	6/2/2015 3:46 PM

398	5	6/2/2015 3:45 PM
399	60	6/2/2015 3:42 PM
400	10	6/2/2015 3:41 PM
401	15	6/2/2015 3:41 PM
402	20	6/2/2015 3:40 PM
403	0	6/2/2015 3:40 PM
404	25	6/2/2015 3:37 PM
405	75-100%	6/2/2015 3:37 PM
406	35	6/2/2015 3:35 PM
407	75	6/2/2015 3:35 PM
408	50	6/2/2015 3:35 PM
409	10	6/2/2015 3:34 PM
410	0	6/2/2015 3:31 PM
411	50	6/2/2015 3:31 PM
412	0	6/2/2015 3:31 PM
413	10%	6/2/2015 3:30 PM
414	0	6/2/2015 3:30 PM
415	100	6/2/2015 3:30 PM
416	20	6/2/2015 3:29 PM
417	10	6/2/2015 3:28 PM
418	100	6/2/2015 3:28 PM
419	5	6/2/2015 3:27 PM
420	50	6/2/2015 3:27 PM
421	50	6/2/2015 3:27 PM
422	100	6/2/2015 3:26 PM
423	30	6/2/2015 3:25 PM
424	10	6/2/2015 3:24 PM
425	10	6/2/2015 3:23 PM
426	30	6/2/2015 3:23 PM
427	60	6/2/2015 3:23 PM
428	100	6/2/2015 3:23 PM
429	30	6/2/2015 3:23 PM
430	65	6/2/2015 3:23 PM
431	0	6/2/2015 3:22 PM
432	20	6/2/2015 3:21 PM

Q14 What is the earliest patient age you start testing:

Answered: 481 Skipped: 15

#	Responses	Date
1	8 yearws	6/19/2015 4:48 PM
2	5 years	6/19/2015 12:50 PM
3	8	6/19/2015 12:32 PM
4	Peak flow3.5-4y. Spirometry 4-6y	6/18/2015 5:24 PM
5	5	6/18/2015 4:49 PM
6	4-6 years (varies depending on the childs ability to do the test)	6/18/2015 12:03 PM
7	5 years old	6/18/2015 9:56 AM
8	5	6/17/2015 4:53 PM
9	7 years	6/17/2015 4:01 PM
10	3 to 3.5 years old	6/17/2015 2:37 PM
11	8 or ten	6/17/2015 10:54 AM
12	3-4y	6/17/2015 9:43 AM
13	5	6/16/2015 9:38 PM
14	6	6/16/2015 9:36 PM
15	5 years of age	6/16/2015 9:00 PM
16	4-5 yrs	6/16/2015 8:33 PM
17	6 YRS IS WHERE THE SPIROMETER STARTS	6/16/2015 8:00 PM
18	12	6/16/2015 7:01 PM
19	10	6/16/2015 6:00 PM
20	5	6/16/2015 5:43 PM
21	5 - 6	6/16/2015 4:09 PM
22	5 years	6/16/2015 3:37 PM
23	3 or 4	6/16/2015 2:42 PM
24	5	6/16/2015 2:24 PM
25	4	6/16/2015 2:10 PM
26	6 y.o	6/16/2015 2:00 PM
27	Seven	6/16/2015 1:36 PM
28	4	6/16/2015 1:22 PM
29	5 years old	6/16/2015 1:01 PM
30	4	6/16/2015 12:55 PM
31	6 years	6/16/2015 12:42 PM
32	4	6/16/2015 12:33 PM

33	5-7	6/16/2015 12:22 PM
34	5-6 years	6/16/2015 12:12 PM
35	6	6/16/2015 11:55 AM
36	5	6/16/2015 11:36 AM
37	Age 4-5 but most can't do it until 6-7- with reliable responses. We still try in the young ones but don't bill if they can't do it properly.	6/16/2015 11:33 AM
38	8 years	6/16/2015 11:33 AM
39	5	6/16/2015 11:18 AM
40	Whenever pateint is capable of consistent repeat testing ususally age 5 but have some 4 year olds	6/16/2015 11:00 AM
41	5	6/16/2015 10:58 AM
42	Teenagers.	6/16/2015 10:38 AM
43	8 years	6/16/2015 10:14 AM
44	10	6/16/2015 10:12 AM
45	5	6/16/2015 10:05 AM
46	6 and above	6/16/2015 10:05 AM
47	6 years	6/16/2015 9:56 AM
48	5	6/16/2015 9:45 AM
49	5 years	6/16/2015 9:30 AM
50	5	6/16/2015 9:29 AM
51	age 6	6/16/2015 9:23 AM
52	9	6/16/2015 9:15 AM
53	Do not see pediatric patients, so age 18	6/16/2015 9:14 AM
54	pre teen	6/16/2015 9:12 AM
55	4 or 5	6/16/2015 9:12 AM
56	4	6/16/2015 9:10 AM
57	5-6	6/16/2015 9:10 AM
58	depends on child	6/16/2015 9:10 AM
59	6 years old	6/16/2015 9:09 AM
60	4 years	6/16/2015 9:09 AM
61	5-7 years	6/16/2015 9:06 AM
62	7	6/16/2015 9:05 AM
63	6 or 7 years of age.	6/15/2015 8:48 PM
64	5yo	6/15/2015 8:44 PM
65	5 years	6/14/2015 9:41 PM
66	6yrs	6/14/2015 8:35 PM
67	about 5 y --a few can do it then.	6/14/2015 7:25 PM
68	depends on patients ability to do test not necessarily age	6/14/2015 4:52 PM
69	4yo	6/14/2015 1:43 AM

70	7 year	6/13/2015 8:39 PM
71	5	6/12/2015 9:10 PM
72	7	6/12/2015 12:27 PM
73	5	6/12/2015 10:34 AM
74	5	6/12/2015 9:05 AM
75	5 or 6 years old	6/12/2015 7:51 AM
76	4	6/12/2015 6:19 AM
77	5 yrs	6/11/2015 11:34 AM
78	Typically age 5-6 for girls, 6-7 for boys	6/11/2015 9:23 AM
79	4or5	6/11/2015 12:00 AM
80	5	6/10/2015 5:29 PM
81	5 years	6/10/2015 3:17 PM
82	6 years old if the child is able to perform the test properly	6/10/2015 2:53 PM
83	5-6 years	6/10/2015 2:43 PM
84	when able to comply	6/10/2015 2:19 PM
85	5	6/10/2015 2:14 PM
86	6	6/10/2015 2:00 PM
87	6	6/10/2015 1:29 PM
88	6 years	6/10/2015 1:19 PM
89	6	6/10/2015 1:13 PM
90	9	6/10/2015 1:04 PM
91	5	6/10/2015 12:50 PM
92	8	6/10/2015 11:58 AM
93	5 years	6/10/2015 11:41 AM
94	6	6/10/2015 11:32 AM
95	depends on the capability of the child, typically 5 or 6	6/10/2015 11:26 AM
96	6	6/10/2015 11:25 AM
97	6	6/10/2015 10:26 AM
98	5	6/10/2015 9:48 AM
99	5 y/o	6/10/2015 9:14 AM
100	4	6/10/2015 9:06 AM
101	n/a	6/10/2015 9:03 AM
102	4	6/10/2015 8:58 AM
103	4	6/10/2015 8:37 AM
104	16 (I only see adults)	6/10/2015 8:08 AM
105	5	6/10/2015 7:53 AM
106	age 5	6/10/2015 6:16 AM
107	6	6/10/2015 6:04 AM

108	6	6/10/2015 5:55 AM
109	8 yrs old	6/10/2015 1:54 AM
110	5-6 years of age; depends on the child and their ability to perform spirometry	6/10/2015 12:39 AM
111	6 months- food, 3 yr aeros	6/9/2015 10:57 PM
112	4 to 5 years old	6/9/2015 10:28 PM
113	5 years of age	6/9/2015 9:56 PM
114	depends on how well they can do spirometry. Generally if over 8 yo, they can do spirometry.	6/9/2015 9:26 PM
115	5	6/9/2015 8:46 PM
116	5-6 yo depending on the individual	6/9/2015 8:03 PM
117	5	6/9/2015 7:51 PM
118	4	6/9/2015 7:48 PM
119	6 years	6/9/2015 7:48 PM
120	3-5	6/9/2015 7:17 PM
121	5 - 6 years	6/9/2015 7:10 PM
122	5	6/9/2015 6:57 PM
123	3	6/9/2015 6:55 PM
124	5 y/o	6/9/2015 6:27 PM
125	3 y.o. if cooperative and coordinated. I only accept these data if the F/V loops and numbers are reproducable.	6/9/2015 6:13 PM
126	4	6/9/2015 6:00 PM
127	4 yrs	6/9/2015 5:25 PM
128	4	6/9/2015 5:23 PM
129	4 years old	6/9/2015 5:21 PM
130	5 years	6/9/2015 5:20 PM
131	4	6/9/2015 5:08 PM
132	6 years	6/9/2015 5:03 PM
133	4	6/9/2015 5:00 PM
134	6	6/9/2015 4:55 PM
135	5 years	6/9/2015 4:53 PM
136	5 or 6	6/9/2015 4:47 PM
137	5 years of age	6/9/2015 4:41 PM
138	age 5 with spirometry; age 4 with oscillometry	6/9/2015 4:24 PM
139	5-6 years	6/9/2015 4:06 PM
140	6	6/9/2015 4:03 PM
141	4-5 years old	6/9/2015 3:56 PM
142	5-6 depending on developmental level	6/9/2015 3:44 PM
143	5	6/9/2015 3:41 PM
144	9-10 y.o.	6/9/2015 3:38 PM
145	5	6/9/2015 3:32 PM

146	age 6	6/9/2015 3:27 PM
147	3 if cooperative per CAMP study	6/9/2015 3:25 PM
148	4-5 years	6/9/2015 3:22 PM
149	5 years	6/9/2015 3:16 PM
150	About 6 YO	6/9/2015 3:10 PM
151	6	6/9/2015 3:08 PM
152	6	6/9/2015 3:01 PM
153	12	6/9/2015 2:55 PM
154	4	6/9/2015 2:50 PM
155	6	6/9/2015 2:49 PM
156	5 years of age	6/9/2015 2:43 PM
157	spirometry: 2 yrs; reversibility tests 3-4 yrs if able to perform consistent PFTs	6/9/2015 2:39 PM
158	4 if can perform initial spirometry	6/9/2015 2:36 PM
159	12	6/9/2015 2:32 PM
160	7 years	6/9/2015 2:31 PM
161	5	6/9/2015 2:29 PM
162	7	6/9/2015 2:25 PM
163	7	6/9/2015 2:23 PM
164	4-6 years old	6/9/2015 2:22 PM
165	5	6/9/2015 2:15 PM
166	About age 5	6/9/2015 1:58 PM
167	4 years old or 6 years old	6/9/2015 1:58 PM
168	12	6/9/2015 1:48 PM
169	5	6/9/2015 1:44 PM
170	adult clinic	6/9/2015 1:40 PM
171	6 or 7	6/9/2015 1:38 PM
172	5yo	6/9/2015 1:29 PM
173	VERY SMART 4 YEAR OLDS; MOST KIDS AT AGE 6 AND ABOVE	6/9/2015 1:27 PM
174	4-5y	6/9/2015 1:27 PM
175	5 years	6/9/2015 1:19 PM
176	above age 10..depends on skill	6/9/2015 1:13 PM
177	5	6/9/2015 1:12 PM
178	5 years	6/9/2015 1:12 PM
179	5	6/9/2015 1:11 PM
180	5	6/9/2015 1:11 PM
181	%	6/9/2015 1:10 PM
182	age 4	6/9/2015 1:07 PM
183	5	6/9/2015 1:07 PM

184	5	6/9/2015 7:41 AM
185	7 or 8	6/9/2015 7:24 AM
186	6	6/9/2015 4:56 AM
187	4 yo	6/9/2015 1:59 AM
188	5 y/o	6/8/2015 8:34 PM
189	6 years	6/8/2015 1:36 PM
190	5	6/8/2015 12:10 PM
191	8-10 y/o	6/8/2015 11:34 AM
192	5	6/8/2015 7:42 AM
193	try to do it in 4 year olds	6/7/2015 9:04 PM
194	ag 6-7	6/7/2015 4:35 PM
195	6	6/7/2015 3:19 PM
196	4 years of age	6/7/2015 2:48 PM
197	6-7	6/7/2015 2:37 PM
198	Age 5. But it depends more on the patient's ability to do the test than age.	6/7/2015 1:33 PM
199	4	6/7/2015 12:01 PM
200	Usually 5-6, but I caution the parents about the reliability of testing at younger ages.	6/6/2015 9:35 PM
201	5 if they can do it	6/6/2015 12:32 PM
202	5.	6/6/2015 12:16 AM
203	5	6/5/2015 8:41 PM
204	approximately 5 yo	6/5/2015 6:30 PM
205	5	6/5/2015 3:32 PM
206	5 years	6/5/2015 2:51 PM
207	6	6/5/2015 2:26 PM
208	6 yo	6/5/2015 1:51 PM
209	9	6/5/2015 12:29 PM
210	4 yrs .	6/5/2015 12:03 PM
211	4	6/5/2015 10:34 AM
212	7 years	6/5/2015 4:40 AM
213	7	6/4/2015 9:32 PM
214	It is completely based on child's ability to perform the test. Some 4 year olds can do it.	6/4/2015 6:33 PM
215	I treat adults	6/4/2015 5:46 PM
216	5	6/4/2015 4:30 PM
217	5 years	6/4/2015 4:28 PM
218	2	6/4/2015 3:21 PM
219	8	6/4/2015 3:15 PM
220	4-5 yeras old	6/4/2015 2:55 PM
221	4 yrs	6/4/2015 1:17 PM

222	Age 5	6/4/2015 1:17 PM
223	4-5	6/4/2015 12:40 PM
224	na	6/4/2015 11:59 AM
225	adult practice	6/4/2015 11:40 AM
226	4 yr old	6/4/2015 10:38 AM
227	5 yrs old	6/4/2015 8:46 AM
228	5	6/4/2015 8:42 AM
229	6	6/4/2015 8:01 AM
230	At any age the patient can demonstrate ability to perform the maneuver satisfactorily	6/4/2015 7:34 AM
231	4	6/4/2015 7:33 AM
232	4 yrs	6/4/2015 7:17 AM
233	4	6/4/2015 7:15 AM
234	3	6/4/2015 5:50 AM
235	5 years	6/3/2015 10:11 PM
236	4 years	6/3/2015 9:43 PM
237	5	6/3/2015 9:37 PM
238	5 y/o	6/3/2015 8:23 PM
239	5 years	6/3/2015 8:17 PM
240	18	6/3/2015 7:56 PM
241	7	6/3/2015 7:45 PM
242	5	6/3/2015 7:35 PM
243	4	6/3/2015 6:48 PM
244	7 years	6/3/2015 5:09 PM
245	6 years	6/3/2015 4:55 PM
246	3 years	6/3/2015 4:54 PM
247	6 years	6/3/2015 4:42 PM
248	6 years	6/3/2015 4:37 PM
249	5 years	6/3/2015 4:14 PM
250	5	6/3/2015 3:37 PM
251	7	6/3/2015 3:33 PM
252	5-6 yo	6/3/2015 3:06 PM
253	7 years. Younger (5yo) depending on child.	6/3/2015 2:43 PM
254	3 years of age, we usually can get reproducible results in most	6/3/2015 2:16 PM
255	6	6/3/2015 2:08 PM
256	6	6/3/2015 1:59 PM
257	6	6/3/2015 1:56 PM
258	6 years	6/3/2015 1:10 PM
259	4-5 years	6/3/2015 1:10 PM

260	12 yr	6/3/2015 1:06 PM
261	5	6/3/2015 12:56 PM
262	5	6/3/2015 12:49 PM
263	age 12	6/3/2015 12:13 PM
264	6	6/3/2015 12:07 PM
265	6	6/3/2015 11:46 AM
266	Age 5	6/3/2015 11:45 AM
267	6	6/3/2015 11:27 AM
268	5	6/3/2015 11:17 AM
269	5	6/3/2015 11:15 AM
270	6	6/3/2015 11:06 AM
271	Down to 6 years old if able to get good cooperation and reliable results.	6/3/2015 11:03 AM
272	5	6/3/2015 10:50 AM
273	5 years	6/3/2015 10:35 AM
274	4 years old	6/3/2015 10:34 AM
275	? PST	6/3/2015 10:26 AM
276	age 8	6/3/2015 10:21 AM
277	Age 5 if mature enough	6/3/2015 10:18 AM
278	4 years	6/3/2015 10:17 AM
279	5 years old	6/3/2015 10:11 AM
280	4 yo	6/3/2015 10:10 AM
281	4 or 5 yo	6/3/2015 9:59 AM
282	6	6/3/2015 9:48 AM
283	7	6/3/2015 9:31 AM
284	10	6/3/2015 9:28 AM
285	Around age 7 depending on the patient.	6/3/2015 9:21 AM
286	4	6/3/2015 9:11 AM
287	6	6/3/2015 8:58 AM
288	7	6/3/2015 8:43 AM
289	3	6/3/2015 8:40 AM
290	5-6 yrs	6/3/2015 8:38 AM
291	6 yo	6/3/2015 8:19 AM
292	6-8 years	6/3/2015 8:14 AM
293	10 years	6/3/2015 8:13 AM
294	6	6/3/2015 8:11 AM
295	4	6/3/2015 8:10 AM
296	5	6/3/2015 8:09 AM
297	5	6/3/2015 8:01 AM

298	4-5	6/3/2015 7:53 AM
299	5	6/3/2015 7:50 AM
300	5	6/3/2015 7:45 AM
301	4 year old	6/3/2015 7:33 AM
302	4	6/3/2015 6:39 AM
303	18. Which is the earliest patient age I see.	6/3/2015 6:31 AM
304	5 years	6/3/2015 6:23 AM
305	6	6/3/2015 6:05 AM
306	10	6/3/2015 5:49 AM
307	5	6/3/2015 4:09 AM
308	5	6/3/2015 3:47 AM
309	6	6/3/2015 2:45 AM
310	5 years	6/3/2015 1:04 AM
311	5 yrs	6/2/2015 11:59 PM
312	6 years	6/2/2015 11:11 PM
313	4	6/2/2015 10:51 PM
314	5 yo	6/2/2015 10:43 PM
315	5	6/2/2015 10:37 PM
316	5	6/2/2015 10:24 PM
317	Age 6	6/2/2015 10:18 PM
318	6 years	6/2/2015 9:59 PM
319	4	6/2/2015 9:59 PM
320	5	6/2/2015 9:52 PM
321	5	6/2/2015 9:49 PM
322	5	6/2/2015 9:39 PM
323	4 years	6/2/2015 9:39 PM
324	6	6/2/2015 9:30 PM
325	20	6/2/2015 9:26 PM
326	Four	6/2/2015 9:20 PM
327	3-4 yrs	6/2/2015 9:16 PM
328	Five	6/2/2015 9:12 PM
329	4 yrs	6/2/2015 9:04 PM
330	4	6/2/2015 9:03 PM
331	5	6/2/2015 9:01 PM
332	3 years.	6/2/2015 8:58 PM
333	4 years	6/2/2015 8:51 PM
334	10yrs	6/2/2015 8:47 PM
335	5	6/2/2015 8:45 PM

336	5	6/2/2015 8:43 PM
337	5 years	6/2/2015 8:42 PM
338	When they are able to perform spirometry	6/2/2015 8:38 PM
339	12	6/2/2015 8:36 PM
340	6	6/2/2015 8:19 PM
341	4	6/2/2015 8:19 PM
342	5 years	6/2/2015 8:07 PM
343	5 years	6/2/2015 8:07 PM
344	four	6/2/2015 8:06 PM
345	6 depending on there ability	6/2/2015 8:03 PM
346	4	6/2/2015 7:59 PM
347	6	6/2/2015 7:57 PM
348	6	6/2/2015 7:56 PM
349	5 years	6/2/2015 7:47 PM
350	5	6/2/2015 7:43 PM
351	5	6/2/2015 7:42 PM
352	4-7 depending upton weight & maturity	6/2/2015 7:31 PM
353	5	6/2/2015 7:25 PM
354	5 yr	6/2/2015 7:13 PM
355	2	6/2/2015 7:10 PM
356	4 years old if cooperative	6/2/2015 7:07 PM
357	4.5-5 years	6/2/2015 7:07 PM
358	4 if possible or whenever they can do the spirometry.	6/2/2015 7:04 PM
359	5 if able to do spirometer. Sometime do peak flow	6/2/2015 7:03 PM
360	5 years	6/2/2015 7:03 PM
361	7	6/2/2015 6:57 PM
362	4y	6/2/2015 6:56 PM
363	4	6/2/2015 6:42 PM
364	probably about 2 years of age but then only if they have sx and we do not try to do PFTs.	6/2/2015 6:42 PM
365	5 years	6/2/2015 6:30 PM
366	6	6/2/2015 6:28 PM
367	6 yo	6/2/2015 6:22 PM
368	6 yrs	6/2/2015 6:18 PM
369	usually 7-8 years old	6/2/2015 6:14 PM
370	4	6/2/2015 6:13 PM
371	5	6/2/2015 6:11 PM
372	6 years old if able	6/2/2015 6:10 PM
373	6	6/2/2015 6:03 PM

374	5 years (depending on ability)	6/2/2015 5:54 PM
375	6	6/2/2015 5:51 PM
376	age 4 depending upon the parent, child interaction	6/2/2015 5:49 PM
377	8	6/2/2015 5:47 PM
378	5 years but in some studies could train to do younger, but not younger than 3 years old.	6/2/2015 5:47 PM
379	usually 7or 8, but my colleagues in peds pulm think they get reliable results in age 6.	6/2/2015 5:40 PM
380	as soon as they can do reproducible spirometry	6/2/2015 5:38 PM
381	I only treat adult patients 18 years of age or older	6/2/2015 5:36 PM
382	5	6/2/2015 5:35 PM
383	5	6/2/2015 5:32 PM
384	5 years old	6/2/2015 5:31 PM
385	I see only adults.	6/2/2015 5:30 PM
386	5 y/o	6/2/2015 5:29 PM
387	12	6/2/2015 5:22 PM
388	4	6/2/2015 5:19 PM
389	10	6/2/2015 5:09 PM
390	4-5 years but typically do not perform reversibility tesing until 9-10 years	6/2/2015 5:09 PM
391	3 years	6/2/2015 5:09 PM
392	i try around age 5	6/2/2015 5:06 PM
393	5y	6/2/2015 5:05 PM
394	girls 6, boys 7	6/2/2015 5:00 PM
395	depends on the patient 4 years and above	6/2/2015 4:54 PM
396	5	6/2/2015 4:54 PM
397	6 yrs old	6/2/2015 4:49 PM
398	5 yrs old	6/2/2015 4:46 PM
399	4	6/2/2015 4:43 PM
400	6 yrs	6/2/2015 4:42 PM
401	5 yo	6/2/2015 4:37 PM
402	14-15	6/2/2015 4:32 PM
403	6-7 years	6/2/2015 4:30 PM
404	5	6/2/2015 4:24 PM
405	6	6/2/2015 4:23 PM
406	5-6 depending on child	6/2/2015 4:18 PM
407	5 years	6/2/2015 4:18 PM
408	6	6/2/2015 4:17 PM
409	6	6/2/2015 4:16 PM
410	6	6/2/2015 4:16 PM
411	6	6/2/2015 4:15 PM

412	6 yr	6/2/2015 4:10 PM
413	4 years	6/2/2015 4:09 PM
414	5	6/2/2015 4:08 PM
415	between 6 and 7---must have maturity	6/2/2015 4:08 PM
416	4	6/2/2015 4:08 PM
417	5	6/2/2015 4:08 PM
418	7	6/2/2015 4:05 PM
419	Typically 6 or older (when they can reliably perform the test)	6/2/2015 4:05 PM
420	4-5 depending on maturity of child and their ability to perform maneuver	6/2/2015 4:04 PM
421	4 or 5 yrs depending on cooperatively & maturity	6/2/2015 4:02 PM
422	5	6/2/2015 4:02 PM
423	5	6/2/2015 4:01 PM
424	6 years	6/2/2015 4:00 PM
425	3	6/2/2015 3:59 PM
426	5-6 yrs old	6/2/2015 3:58 PM
427	5-6	6/2/2015 3:57 PM
428	6	6/2/2015 3:56 PM
429	7	6/2/2015 3:56 PM
430	6	6/2/2015 3:56 PM
431	5	6/2/2015 3:55 PM
432	8	6/2/2015 3:54 PM
433	5	6/2/2015 3:54 PM
434	4	6/2/2015 3:52 PM
435	5	6/2/2015 3:50 PM
436	4 years	6/2/2015 3:50 PM
437	4-6 y/o	6/2/2015 3:50 PM
438	5-6	6/2/2015 3:49 PM
439	4 years old	6/2/2015 3:48 PM
440	5 years	6/2/2015 3:46 PM
441	Seven	6/2/2015 3:45 PM
442	4	6/2/2015 3:42 PM
443	5	6/2/2015 3:41 PM
444	4	6/2/2015 3:41 PM
445	4 years old	6/2/2015 3:40 PM
446	I have tried at as low as 4 years old, depending on the patient's cooperativeness. If I cannot get good-looking, consistent readings on a child, I do not charge for the test.	6/2/2015 3:38 PM
447	5	6/2/2015 3:37 PM
448	as early as they can cooperate, often 6 y/o	6/2/2015 3:37 PM

449	around age 5	6/2/2015 3:37 PM
450	6	6/2/2015 3:35 PM
451	5 yrs	6/2/2015 3:35 PM
452	7 (some girls can perform reliable tests around 5-6)	6/2/2015 3:35 PM
453	5	6/2/2015 3:34 PM
454	around 5. every once in a while a 4.5 year old will do okay. many times, you have to wait until 6.	6/2/2015 3:32 PM
455	5	6/2/2015 3:31 PM
456	5-6	6/2/2015 3:31 PM
457	6 yo	6/2/2015 3:31 PM
458	6	6/2/2015 3:31 PM
459	5	6/2/2015 3:30 PM
460	4	6/2/2015 3:30 PM
461	6 years or after completing kindergarten	6/2/2015 3:30 PM
462	5	6/2/2015 3:29 PM
463	5	6/2/2015 3:29 PM
464	5	6/2/2015 3:28 PM
465	5	6/2/2015 3:28 PM
466	6	6/2/2015 3:27 PM
467	5 years	6/2/2015 3:27 PM
468	a rare 4 year old, some 5 year old, most by age 6	6/2/2015 3:27 PM
469	5 yrs	6/2/2015 3:26 PM
470	12	6/2/2015 3:25 PM
471	Only see adults	6/2/2015 3:25 PM
472	4	6/2/2015 3:24 PM
473	4	6/2/2015 3:23 PM
474	4	6/2/2015 3:23 PM
475	5 years	6/2/2015 3:23 PM
476	10	6/2/2015 3:23 PM
477	5	6/2/2015 3:23 PM
478	6	6/2/2015 3:23 PM
479	7	6/2/2015 3:23 PM
480	8	6/2/2015 3:22 PM
481	5 years	6/2/2015 3:21 PM

Q15 If insurance only allows a certain number of tests (code 94060) per year, but your patient has exceeded this amount, what do you do?

Answered: 446 Skipped: 50

#	Responses	Date
1	Still do it when deemed necessary, at times without charging	6/19/2015 4:48 PM
2	whatever is best for the patient	6/19/2015 12:50 PM
3	Do not test	6/19/2015 12:32 PM
4	All insurances that I deal with allows q6 mo visits, I will do especially if the pt is sick.Pt with poorly controlled asthma seen 2-4 weeks after their first visit and then q6mo.	6/18/2015 5:24 PM
5	n/a - but would not likely do it	6/18/2015 4:49 PM
6	I don't look at that. Im not sure what the code is (Bronchospasm eval bundle- I only do one a year- and usually only do once ever) If it is for PFT with flow, volume loop- if I need it clinically I order it. The biller can usually justify the additional based on increased symptoms of ned to assess results with a medication change.	6/18/2015 12:03 PM
7	Do it if iclinically indicated	6/18/2015 9:56 AM
8	explain to the patient the need for the test and have them pay a reasonable amount	6/17/2015 4:53 PM
9	Eat up the cost	6/17/2015 4:01 PM
10	I work for an HMO, who has never restricted me from doing tests. If I were to be restricted, I would use a peak flow meter and the patient's albuterol HFA inhaler to do a pre- and post-BD assessment.	6/17/2015 2:37 PM
11	I do not know	6/17/2015 10:54 AM
12	do as needed to make proper assessment	6/16/2015 9:38 PM
13	We haven't faced this yet	6/16/2015 9:36 PM
14	We may do the test and not charge	6/16/2015 9:00 PM
15	do the test and don't charge the patient	6/16/2015 8:33 PM
16	NEVER EVER HAS HAPPENED IN 20 YEARS 94060 94375 94010	6/16/2015 8:00 PM
17	Do as needed	6/16/2015 7:01 PM
18	I do it anyway	6/16/2015 6:00 PM
19	do them anyway	6/16/2015 5:43 PM
20	still do test	6/16/2015 4:09 PM
21	still perform test	6/16/2015 3:37 PM
22	EAT it	6/16/2015 2:42 PM
23	test if needed	6/16/2015 2:24 PM
24	Unsure	6/16/2015 2:10 PM
25	Still do it	6/16/2015 2:00 PM
26	No charge if needed	6/16/2015 1:36 PM
27	I do the test anyway and I never make the patient pay	6/16/2015 1:22 PM
28	Depends on the patient and medical necessity of the test	6/16/2015 1:01 PM

29	Order a methacholine, and if they don't pay in the office, order at a hospital. Do insurance companies limit blood sugars on a patients with diabetes??	6/16/2015 12:55 PM
30	advise patient of out of pocket cost prior to testing	6/16/2015 12:42 PM
31	i have not had this situation yet	6/16/2015 12:33 PM
32	Uncommon needing to do this frequently	6/16/2015 12:22 PM
33	It has never come up.	6/16/2015 11:33 AM
34	n/a to my office	6/16/2015 11:33 AM
35	I do what is clinically necessary and not charge	6/16/2015 11:18 AM
36	Test if indicated at no cost	6/16/2015 11:00 AM
37	It has never been a problem in my practice.	6/16/2015 10:38 AM
38	N/A	6/16/2015 10:14 AM
39	Appeal	6/16/2015 10:12 AM
40	i have never run into this situation	6/16/2015 10:05 AM
41	tes anyway if indicated	6/16/2015 9:56 AM
42	this has not occurred in our practice. PFTs have not been limited for us so far.	6/16/2015 9:30 AM
43	Skip the test. Fortunately this is not a frequent issue for us.	6/16/2015 9:29 AM
44	send letter of medical neccesity	6/16/2015 9:23 AM
45	Still perform to reflect accuracy and direction of treatment.	6/16/2015 9:15 AM
46	Haven't had this problem	6/16/2015 9:14 AM
47	Never had this problem. Then again I don't do excessive number of reversibility testing	6/16/2015 9:12 AM
48	Have not encountered this problem	6/16/2015 9:12 AM
49	Test only if absolutely NECESSARY	6/16/2015 9:10 AM
50	I have not encountered this problem.	6/16/2015 9:10 AM
51	Perform study with obtaining ABN signature	6/16/2015 9:10 AM
52	ask them if they would like to proceed & pay out of pocket.	6/16/2015 9:09 AM
53	No bill	6/16/2015 9:09 AM
54	do the test and get shafted.	6/16/2015 9:06 AM
55	base on clinical need and explain that the test may not be covered.	6/16/2015 9:05 AM
56	not charge the patient	6/15/2015 8:48 PM
57	Have not had this problem	6/15/2015 8:44 PM
58	If I need the information, I do not charge for the test	6/14/2015 9:41 PM
59	tricare military insurance have no limitations.	6/14/2015 8:35 PM
60	haven't run into that problem--rarely do more than 2 per year unless patient is unstable and the information is important for management	6/14/2015 7:25 PM
61	have never been presented with this issue; if a patient refuses a spirometry I ask why and obviously abide by their wishes after explaining why I believe the test is necessary	6/14/2015 4:52 PM
62	Do it for free	6/14/2015 1:43 AM
63	Perform PFT and wright off charge	6/13/2015 8:39 PM
64	Perform spirometry, but expect no payment	6/12/2015 9:10 PM

65	do the test at no charge	6/12/2015 12:27 PM
66	Test without charge	6/12/2015 10:34 AM
67	has not been a problem	6/12/2015 9:05 AM
68	not applicable as I do not participate in any insurance plans	6/12/2015 7:51 AM
69	Ask patient if they are willing to pay	6/12/2015 6:19 AM
70	not applicable in my practice	6/11/2015 11:34 AM
71	Don't do the test, rely on ambulatory peak flows, symptoms ACT, other tools to assess for control	6/11/2015 9:23 AM
72	was not aware of this	6/11/2015 12:00 AM
73	if needed, test and don't get reimbursed	6/10/2015 5:29 PM
74	write off amount	6/10/2015 3:17 PM
75	If I need to do the test I do it and write off the charges.	6/10/2015 2:53 PM
76	Still do it if necessary	6/10/2015 2:43 PM
77	continue to test	6/10/2015 2:19 PM
78	do the test and not get paid for it	6/10/2015 2:14 PM
79	we don't keep track	6/10/2015 1:29 PM
80	I do the test and write off the cost.	6/10/2015 1:19 PM
81	n/a	6/10/2015 1:13 PM
82	not test	6/10/2015 1:04 PM
83	Assess the need for such frequent testing and perform testing based on clinical need.	6/10/2015 12:50 PM
84	depends on clinical scenario	6/10/2015 11:58 AM
85	charge the patient	6/10/2015 11:41 AM
86	Still do it and not charge, unfortunately	6/10/2015 11:32 AM
87	still test if indicated	6/10/2015 11:26 AM
88	Has never occured	6/10/2015 11:25 AM
89	I am not aware of any limit	6/10/2015 10:26 AM
90	Has not happened to date.	6/10/2015 9:48 AM
91	Still do it.	6/10/2015 9:14 AM
92	?	6/10/2015 9:06 AM
93	n/a	6/10/2015 9:03 AM
94	Do the test anyway, whether we get paid or not. I order the test for clinical management, and that need doesn't disappear just because I don't get paid.	6/10/2015 8:58 AM
95	haven't encountered this.	6/10/2015 8:37 AM
96	Dont charge	6/10/2015 8:08 AM
97	Perform test and not charge for it.	6/10/2015 6:16 AM
98	Do it appeal chrage	6/10/2015 5:55 AM
99	I still proceed with study despite no reimbursement	6/10/2015 1:54 AM
100	We would traditionally only do this once a year and a follow up FEV1; we typically monitor thereafter with PEFRs and get an annual spirometry to ensure lung function is stable.	6/10/2015 12:39 AM
101	Flow via peak flow Provide cash cost to patient Do 94010 instead	6/9/2015 10:57 PM

102	Nothing	6/9/2015 10:28 PM
103	Do the test if symptomatic at follow-up and don't bill. If not symptomatic do not do the test.	6/9/2015 9:56 PM
104	Never been an problem in my practice.	6/9/2015 9:26 PM
105	Don't charge for the test	6/9/2015 8:46 PM
106	Not applicable in Canada	6/9/2015 8:03 PM
107	N/a	6/9/2015 7:51 PM
108	do it anyway at no charge	6/9/2015 7:48 PM
109	n/a	6/9/2015 7:48 PM
110	TEST ANYHOW WITH OUT CHARGE	6/9/2015 7:17 PM
111	test	6/9/2015 7:10 PM
112	perform the test without charging the patient.	6/9/2015 6:57 PM
113	I do not have an awareness of this limitation	6/9/2015 6:55 PM
114	Do PFT the minimum required times	6/9/2015 6:27 PM
115	Never had that issue, but I would still do it. It's better than having to answer a page from a decompensating asthmatic at 3AM.	6/9/2015 6:13 PM
116	will test if clinical necessary	6/9/2015 6:00 PM
117	Billing more than 1 pre-post study in a year has never been done in this office.	6/9/2015 5:23 PM
118	I make a judgment about whether to get by without this test but it is rare in my practice to exceed that number allowed.	6/9/2015 5:21 PM
119	do the tests needed and write it off	6/9/2015 5:20 PM
120	Explain to them that their insurance plan does not pay for the service before they exceed the amount.	6/9/2015 5:08 PM
121	perform test, submit charge and write it off if not reimbursed	6/9/2015 5:03 PM
122	do it and eat the bill	6/9/2015 5:00 PM
123	Has not happened to me	6/9/2015 4:55 PM
124	Not applicable	6/9/2015 4:53 PM
125	Haven't yet but would tell them to let the insurance company CEO manage their Asthma when they end up in ER	6/9/2015 4:47 PM
126	dont do test, as cost becomes prohibitive for family	6/9/2015 4:41 PM
127	charge pt the additional but will sometimes forgo charges.	6/9/2015 4:24 PM
128	test anyway	6/9/2015 4:06 PM
129	do it anyway and pay for it myself to an extent	6/9/2015 4:03 PM
130	Since this a an important test, especially for poor perceivers of symptoms and for medication titration, I would still do the test and not charge the patient.	6/9/2015 3:56 PM
131	Hospital eats cost	6/9/2015 3:44 PM
132	usually only perform once on a patient	6/9/2015 3:41 PM
133	Do the test and not charge for it	6/9/2015 3:38 PM
134	continue to do the test	6/9/2015 3:32 PM
135	NA (Canada)	6/9/2015 3:27 PM
136	bill 94010	6/9/2015 3:25 PM
137	perform test if clinically indicated	6/9/2015 3:22 PM

138	Work with insurance to explain the need for the test or defer.	6/9/2015 3:16 PM
139	Immunocap	6/9/2015 3:10 PM
140	N/A	6/9/2015 3:08 PM
141	I never exceed the number allowed per year.	6/9/2015 2:55 PM
142	I have not had this happen.	6/9/2015 2:50 PM
143	?	6/9/2015 2:49 PM
144	never happened	6/9/2015 2:43 PM
145	N/A	6/9/2015 2:39 PM
146	Test anyway	6/9/2015 2:32 PM
147	test and not get paid	6/9/2015 2:31 PM
148	test them anyway	6/9/2015 2:29 PM
149	Delay testing	6/9/2015 2:25 PM
150	Not bill patient.	6/9/2015 2:23 PM
151	Patient gets charged.	6/9/2015 2:22 PM
152	Work with patient but so far is a non issue	6/9/2015 1:58 PM
153	?	6/9/2015 1:58 PM
154	perform test if clinically indicated	6/9/2015 1:48 PM
155	Do for free	6/9/2015 1:44 PM
156	not applicable - we don't perform that many	6/9/2015 1:40 PM
157	do w/o charge	6/9/2015 1:38 PM
158	If I feel it will aid in management, I will still do test.	6/9/2015 1:29 PM
159	DO THEM AND NOT GET PAID!	6/9/2015 1:27 PM
160	still test, do not charge but to be honest I'm not sure that occurs?	6/9/2015 1:27 PM
161	never happened	6/9/2015 1:19 PM
162	do it gratis	6/9/2015 1:13 PM
163	Base testing only on perception of clinical need	6/9/2015 1:12 PM
164	Haven't tested enough to have this problem. Maybe providers should ask themselves why they're doing so much spiro.	6/9/2015 1:11 PM
165	I do the test if it can help with diagnosis and control and eat the cost.	6/9/2015 1:10 PM
166	Test anyway	6/9/2015 1:07 PM
167	explain rationale for testing and have patient sign ABN form	6/9/2015 7:41 AM
168	n/a Most of my patients are capitated.	6/9/2015 7:24 AM
169	Perform the test anyway	6/9/2015 1:59 AM
170	do testing, bill only 94010	6/8/2015 8:34 PM
171	I have not come across the problem as yet	6/8/2015 1:36 PM
172	this has not come up in our office	6/8/2015 12:10 PM
173	Eat the cost or use peak flow measurements	6/8/2015 11:34 AM
174	test anyway (not much incremental cost)	6/8/2015 7:42 AM

175	bill the patient if they are willing to pay and their insurance allow it or do the test free of charge	6/7/2015 9:04 PM
176	Still do and may not charge patient	6/7/2015 4:35 PM
177	I would still perform the test if patient needs it. My office will write off a lot of these tests if something like that happens.	6/7/2015 3:19 PM
178	administer the test and discount charge 90% or do it for free	6/7/2015 2:48 PM
179	Depends on situation.	6/7/2015 2:37 PM
180	Never had this problem.	6/7/2015 1:33 PM
181	Test	6/7/2015 12:01 PM
182	I do not think that this has been a big problem. If a patient is reluctant to get the test because they have a high deductible, I will not charge for it. If the insurance company limits the number of tests that I need, I would do the same, because I am not willing to compromise patient care.	6/6/2015 9:35 PM
183	do the tests free	6/6/2015 12:32 PM
184	I do not believe that has been an issue. Honestly, I would do it infrequently or never if I knew I would not get paid for it. I would, perhaps, tell my patient that I would like to test and would charge them for it. Even more honestly, I have never done it.	6/6/2015 12:16 AM
185	has not come up yet	6/5/2015 8:41 PM
186	we do not have any insurance companies with that limitation that I am aware of	6/5/2015 6:30 PM
187	Never happened.	6/5/2015 3:32 PM
188	Don't charge the patient	6/5/2015 2:51 PM
189	N/A	6/5/2015 2:26 PM
190	test	6/5/2015 1:51 PM
191	Perform the test for no cost	6/5/2015 12:29 PM
192	get prior approval	6/5/2015 12:03 PM
193	do it anyway and waive the charge	6/5/2015 10:34 AM
194	Not charge them	6/4/2015 9:32 PM
195	If I Think it is important - then we do it anyway and dont bill for it	6/4/2015 6:33 PM
196	Never happens	6/4/2015 5:46 PM
197	Perform PFT when needed or defer to a peak flow meter	6/4/2015 4:30 PM
198	Still order it	6/4/2015 4:28 PM
199	do the test and write-off the cost	6/4/2015 3:21 PM
200	ask if they would pay for materials cost	6/4/2015 2:55 PM
201	Have not had that happen.	6/4/2015 1:17 PM
202	Do it anyway. We need to be reimbursed.	6/4/2015 1:17 PM
203	perform the test if clinically indicated	6/4/2015 12:40 PM
204	na	6/4/2015 11:59 AM
205	not applicable	6/4/2015 11:40 AM
206	still perform test	6/4/2015 10:38 AM
207	do what is best for the pt, if more is needed, then more is done	6/4/2015 8:46 AM
208	Try to do without if possible	6/4/2015 8:42 AM
209	do it for free	6/4/2015 8:01 AM

210	I haven't encountered that problem	6/4/2015 7:34 AM
211	not charge pt	6/4/2015 7:33 AM
212	Defer the test	6/4/2015 7:17 AM
213	N/A for me	6/4/2015 7:15 AM
214	Do the test	6/4/2015 5:50 AM
215	Still perform test if needed.	6/3/2015 10:11 PM
216	Include this test anyway as part of follow up visit if indicated	6/3/2015 9:43 PM
217	Offer a test at a fixed discounted price or if need be at no charge NOTE: Many patients are referred to me whose asthma is managed by a pulmonologist or primary care Dr. who contracts with a respiratory therapy service for asthma management. The numbers in question 14 would be much higher if I were managing the asthma in all of the patients in my allergy practice.	6/3/2015 9:37 PM
218	just use history and exam to assess	6/3/2015 8:23 PM
219	No charge	6/3/2015 8:17 PM
220	Still do it	6/3/2015 7:45 PM
221	continue testing	6/3/2015 7:35 PM
222	don't charge	6/3/2015 6:48 PM
223	Not do the test	6/3/2015 5:09 PM
224	Has not been a problem	6/3/2015 4:55 PM
225	Never had denial	6/3/2015 4:54 PM
226	PEF monitoring at home	6/3/2015 4:42 PM
227	So far I don't think we have exceeded the set limit	6/3/2015 4:37 PM
228	Do only if changes therapy I would choose	6/3/2015 4:14 PM
229	Ask the patient to pay out of pocket.	6/3/2015 3:37 PM
230	Be more judicious about use - only when I believe results would significantly change my management.	6/3/2015 2:43 PM
231	test	6/3/2015 2:16 PM
232	do it anyway	6/3/2015 2:08 PM
233	discuss cost with patient. If unable to pay and very strong need for test will write off, if able to skip testing this visit due to good control, etc then will skip	6/3/2015 1:59 PM
234	Would still test	6/3/2015 1:56 PM
235	"eat" the cost	6/3/2015 1:10 PM
236	I eat it.	6/3/2015 1:10 PM
237	n/a	6/3/2015 1:06 PM
238	Eat it.	6/3/2015 12:56 PM
239	not charge	6/3/2015 12:49 PM
240	We do what is appropriate for patient care.	6/3/2015 12:13 PM
241	Nothing	6/3/2015 11:46 AM
242	If the test is clincally indicated I do the test.	6/3/2015 11:45 AM
243	do the test; bill insurance and hope for the best; appeal if needed	6/3/2015 11:27 AM
244	Not sure yet, new to clinical practice	6/3/2015 11:15 AM
245	peak flow	6/3/2015 11:06 AM

246	Do the test if needed to make clinical decisions and just write off the cost. If the patient needs the test, they need the test. I won't let the insurance company dictate what I do in the patient's best interest and care. If you decide not to do the test just because the insurance won't pay, you probably didn't need to do the test anyway.	6/3/2015 11:03 AM
247	rarely exceed it	6/3/2015 10:50 AM
248	n/a	6/3/2015 10:35 AM
249	Do the test anyway	6/3/2015 10:34 AM
250	Waive the balance	6/3/2015 10:26 AM
251	use once a year	6/3/2015 10:21 AM
252	Do it anyway.	6/3/2015 10:18 AM
253	n/a	6/3/2015 10:17 AM
254	N/A. This has never come up	6/3/2015 10:11 AM
255	still do the test.	6/3/2015 10:10 AM
256	I still use the PFT test if indicated because it is the right thing to do for good patient care.	6/3/2015 9:59 AM
257	dont do it	6/3/2015 9:48 AM
258	Do the test but write off the charge	6/3/2015 9:31 AM
259	Treat and monitor when symptomatic.	6/3/2015 9:28 AM
260	Do them anyway.	6/3/2015 9:21 AM
261	Do it and don't bill them	6/3/2015 9:11 AM
262	If testing necessary, will do test and write-off	6/3/2015 8:58 AM
263	Do not perform	6/3/2015 8:43 AM
264	We test when needed	6/3/2015 8:40 AM
265	Sometimes do peak flow measurements instead of spirometry.	6/3/2015 8:38 AM
266	have not encountered this issue as of yet	6/3/2015 8:19 AM
267	Do spiro only if necessary, I.e. Worsened symptoms	6/3/2015 8:14 AM
268	I don't typically perform reversibility more than once on a given patient. However, if I thought the test was needed, I would perform it and risk not being paid.	6/3/2015 8:13 AM
269	Don't know that at time of testing. Proceed anyway unless patient knows it is not covered, then we bypass it until next year or use pre-/-post peak flows instead.	6/3/2015 8:11 AM
270	Continue to check spirometry but write it off	6/3/2015 8:10 AM
271	n/a	6/3/2015 8:09 AM
272	It has never happened in over 25 years. We don't need monthly spirometry! If it happens, I will need to talk with the patient and explain why and what financial implications that may have.	6/3/2015 8:01 AM
273	We do whatever is clinicly warranted for the care and benfit of the patient regardless of insurance coverage	6/3/2015 7:53 AM
274	No further testing. Guess.	6/3/2015 7:50 AM
275	N/A	6/3/2015 7:45 AM
276	Perform test and try to get it covered by insurance	6/3/2015 7:33 AM
277	?	6/3/2015 6:39 AM
278	Nothing. Don't bill.	6/3/2015 6:31 AM
279	still test, write off charge	6/3/2015 6:23 AM
280	write it off	6/3/2015 6:05 AM

281	N/A	6/3/2015 5:49 AM
282	adjust cost	6/3/2015 4:09 AM
283	Have not come across this issue	6/3/2015 3:47 AM
284	Write off	6/3/2015 2:45 AM
285	do it anyway gratis	6/3/2015 1:04 AM
286	If I feel it is seriously needed to guide treatment in a patient who is doing poorly, I will "no charge" it and use it for my benefit in decision making regardless. Otherwise, rely on peak flow and reported symptoms and use of SABA	6/2/2015 11:59 PM
287	Do it anyway and don't charge, but rarely comes up	6/2/2015 11:11 PM
288	Not applicable	6/2/2015 10:51 PM
289	No charge	6/2/2015 10:43 PM
290	do it anyway	6/2/2015 10:37 PM
291	Do test since I am not aware of this info prior to doing test	6/2/2015 10:24 PM
292	Perform it anyway if needed. Rare problem	6/2/2015 10:18 PM
293	Still perform test regardless	6/2/2015 9:59 PM
294	I don't know that this has happened in my practice yet. Likely write off the cost	6/2/2015 9:59 PM
295	I only do it if I really need it.	6/2/2015 9:52 PM
296	treat patient appropriately regardless of coding	6/2/2015 9:49 PM
297	has not happened that i am aware but would likely due test	6/2/2015 9:39 PM
298	do the test and absorb the cost	6/2/2015 9:39 PM
299	n/a	6/2/2015 9:30 PM
300	N/A	6/2/2015 9:26 PM
301	I have never had a denial due to excessive testing; but I have never used this code more than 3 times in a year.	6/2/2015 9:20 PM
302	do free	6/2/2015 9:16 PM
303	Do the test if needed after discussion with the patient and if not covered do it gratis	6/2/2015 9:12 PM
304	Test and do not bill	6/2/2015 9:04 PM
305	write letter to insurance company explaining why this patients needs testing and why it is extrely cost effective	6/2/2015 9:03 PM
306	Do it for free. I want the best for my patients	6/2/2015 9:01 PM
307	Talk to the pt and ask how they would like to proceed.	6/2/2015 8:58 PM
308	n/a	6/2/2015 8:51 PM
309	Not encounterd this problem	6/2/2015 8:47 PM
310	That scenario hasn't occurred	6/2/2015 8:45 PM
311	Have not come across this problem	6/2/2015 8:43 PM
312	I only do spirometry once a year and occasionally have to do do it again within the year. Usually I would not do bronchodilator challenge in a stable asthmatic controlled on current controllers. I use 94010, in the established patients.	6/2/2015 8:42 PM
313	No answer	6/2/2015 8:38 PM
314	Use clinical judgement only	6/2/2015 8:36 PM
315	has not been a problem	6/2/2015 8:19 PM
316	Not charge	6/2/2015 8:19 PM

317	Don't know	6/2/2015 8:07 PM
318	I do the test without charging	6/2/2015 8:07 PM
319	eat the cost. I don't look at it during visits, only quarterly at billing review	6/2/2015 8:06 PM
320	unsure	6/2/2015 8:03 PM
321	If necessary get it and write of the cost. If discretionary, then only annual at most	6/2/2015 7:59 PM
322	Do it anyway	6/2/2015 7:57 PM
323	Do it anyway	6/2/2015 7:56 PM
324	perform the teat anyway	6/2/2015 7:47 PM
325	N/a	6/2/2015 7:42 PM
326	Still do it for patient care	6/2/2015 7:31 PM
327	Unaware of limit	6/2/2015 7:25 PM
328	Never encountered this issue.	6/2/2015 7:13 PM
329	have not had this issue so far	6/2/2015 7:10 PM
330	Do what is necessary and don't charge if not covered.	6/2/2015 7:07 PM
331	Repeat only if absolutely indicated, medically necessity	6/2/2015 7:07 PM
332	I will have to write off the charges.	6/2/2015 7:04 PM
333	Don't have to charge or. If needed will do it!	6/2/2015 7:03 PM
334	Na	6/2/2015 7:03 PM
335	Perform test, but bill 94010	6/2/2015 6:56 PM
336	Still do it to aid management or diagnosis	6/2/2015 6:42 PM
337	We do the test if we need it. Same for FeNO since only about 1/2 the insurance companies we deal with will reimburse for FeNO and then not very well.	6/2/2015 6:42 PM
338	NA	6/2/2015 6:30 PM
339	will use 94010	6/2/2015 6:28 PM
340	hmm- never knew this was an issue.	6/2/2015 6:22 PM
341	appeal	6/2/2015 6:18 PM
342	I don't think we've ever had that problem	6/2/2015 6:14 PM
343	I do it anyway and eat the cost	6/2/2015 6:13 PM
344	do test anyway and not bill	6/2/2015 6:11 PM
345	I usually don't exceed the amount. We do every 6 months at the most and obtain insurance benefits prior to 1st visit. It has never been an issue. If not covered at all we give discount cash pricing.	6/2/2015 6:10 PM
346	free for pt. Perform it anyway.	6/2/2015 6:03 PM
347	not sure we have encountered that	6/2/2015 5:54 PM
348	n/a	6/2/2015 5:51 PM
349	do not charge for the test. It is an important "vital signs" and important to help determine control. The stethoscope is nowhere near as accurate as the spirometer.	6/2/2015 5:49 PM
350	n/a	6/2/2015 5:47 PM
351	Cover the cost.	6/2/2015 5:47 PM
352	very clever, of course, then I would use FeNO! But the military doesn't have quotas (yet) and allergy doesn't have FeNo yet.	6/2/2015 5:40 PM

353	Don't get to see people that often	6/2/2015 5:38 PM
354	this has not been an issue for me (yet?)	6/2/2015 5:36 PM
355	perform only spirometry with no bronchodilator	6/2/2015 5:35 PM
356	i do the test whether covered or not if the result will help with medical decision making	6/2/2015 5:32 PM
357	quit performing the tests	6/2/2015 5:31 PM
358	N/A	6/2/2015 5:30 PM
359	Rarely a problem. Will do test w/o. Charge	6/2/2015 5:29 PM
360	Adjust charges	6/2/2015 5:22 PM
361	Appeal the decision. Send records to support why this was done.	6/2/2015 5:19 PM
362	still do test but not bill	6/2/2015 5:09 PM
363	not sure this is issue	6/2/2015 5:09 PM
364	I am not involved with the business side of the practice. I think the staff discusses the issue with the patients. However, I don't recall doing this test more than once per patient.	6/2/2015 5:09 PM
365	Perform and write off charge	6/2/2015 5:06 PM
366	hasn't happened	6/2/2015 5:05 PM
367	have not run into the problem as I a light tester	6/2/2015 5:00 PM
368	write off the amount, i still do the test to help me in assessing the patient	6/2/2015 4:54 PM
369	offer free testing	6/2/2015 4:54 PM
370	recommend it to patient and then let patient determine if they want to pay for the test	6/2/2015 4:49 PM
371	I do PFTs at baseline (unless recently done elsewhere - in which case, I get those results). I usually only do PFTs once a year on patients	6/2/2015 4:46 PM
372	Do the test but do not charge for it - this rarely occurs as I typically only do this once or twice a year.	6/2/2015 4:43 PM
373	have not had this arise	6/2/2015 4:42 PM
374	do the test and eat it if indicated.	6/2/2015 4:37 PM
375	don't bill	6/2/2015 4:32 PM
376	Not apply. I never do more than one.	6/2/2015 4:30 PM
377	seldom do more than 1 test per year	6/2/2015 4:24 PM
378	not test	6/2/2015 4:23 PM
379	None of insurance companies do this.	6/2/2015 4:18 PM
380	curse under my breath and write of the charge	6/2/2015 4:18 PM
381	n/a	6/2/2015 4:17 PM
382	If this happened I would have to eat it	6/2/2015 4:16 PM
383	I do the test anyway	6/2/2015 4:16 PM
384	94010	6/2/2015 4:15 PM
385	Do it anyway if indicated.	6/2/2015 4:09 PM
386	HAve not come across that but would still do the test if indicated	6/2/2015 4:08 PM
387	never had this happen	6/2/2015 4:08 PM
388	have not had that happen yet but it would not change what we do	6/2/2015 4:08 PM
389	waive the fee	6/2/2015 4:08 PM

390	Don't do test or charge patient if they agree to do test anyway?	6/2/2015 4:05 PM
391	Have never faced this problem.	6/2/2015 4:05 PM
392	The test is done if needed and the cost is absorbed by the parctice	6/2/2015 4:04 PM
393	If having exacerbation or increase sx, do test & don't get paid	6/2/2015 4:02 PM
394	will do test if I really want it	6/2/2015 4:02 PM
395	never had this denied	6/2/2015 4:01 PM
396	Never encounter this	6/2/2015 4:00 PM
397	Monitor closely with PFTs.	6/2/2015 3:58 PM
398	If clinically indicated perform free of charge.	6/2/2015 3:57 PM
399	no-bill if necessary though this has never happened	6/2/2015 3:56 PM
400	lose money and test if indicated	6/2/2015 3:56 PM
401	never happened	6/2/2015 3:55 PM
402	Patient signs an advanced beneficiary notice and is responsible for payment	6/2/2015 3:54 PM
403	Don't charge	6/2/2015 3:54 PM
404	We do not encounter this issue.	6/2/2015 3:52 PM
405	ask patient before obtaining spirometry	6/2/2015 3:50 PM
406	May provide for free	6/2/2015 3:50 PM
407	I rarely do more than one 94060 and so I am not sure, I would probably request they reconsider	6/2/2015 3:50 PM
408	Hasn't been an issue	6/2/2015 3:49 PM
409	Discuss the situation with the patient. Proceed and bill patient, if agreeable.	6/2/2015 3:48 PM
410	Hasn't been an issue	6/2/2015 3:45 PM
411	Have not run into this scenario, but would still do the test (cost is minimal, information is important)	6/2/2015 3:42 PM
412	do the test	6/2/2015 3:41 PM
413	Still test and pt responsible for payment if not covered	6/2/2015 3:41 PM
414	I don't keep track	6/2/2015 3:40 PM
415	absorb cost	6/2/2015 3:40 PM
416	I do what is medically needed.	6/2/2015 3:38 PM
417	This hasn't come up for me.	6/2/2015 3:37 PM
418	military do not worry about payment	6/2/2015 3:37 PM
419	I work in Kaiser and I don't have a limit of tests	6/2/2015 3:37 PM
420	still do test if clinically indicated	6/2/2015 3:35 PM
421	If needed we do test and write off charge	6/2/2015 3:35 PM
422	not sure, I don't think I have been in this situation	6/2/2015 3:35 PM
423	I've not encountered this problem	6/2/2015 3:34 PM
424	haven't encountered this	6/2/2015 3:32 PM
425	I didn't know that.	6/2/2015 3:31 PM
426	Eat it.	6/2/2015 3:31 PM

427	Do it anyway if necessary and won't charge for procedure. However, can't imagine having to do bronchodilator reversibility testing multiple times a year.	6/2/2015 3:31 PM
428	Ask billing or write it off	6/2/2015 3:30 PM
429	they never exceed the amount allowed	6/2/2015 3:30 PM
430	I would not do it at that point	6/2/2015 3:30 PM
431	review	6/2/2015 3:29 PM
432	not sure	6/2/2015 3:29 PM
433	Do tests anyway	6/2/2015 3:28 PM
434	nc	6/2/2015 3:28 PM
435	If I need it, do it anyway.	6/2/2015 3:27 PM
436	Perform the spirometry anyway	6/2/2015 3:27 PM
437	perform at n/c	6/2/2015 3:27 PM
438	eat it	6/2/2015 3:26 PM
439	Not applicable	6/2/2015 3:25 PM
440	I wouldn't do the test if I won't get compensated for nursing and doctor time unless I thought it was critical to the assessment.	6/2/2015 3:24 PM
441	I haven't been aware of this problem.	6/2/2015 3:23 PM
442	never occurred	6/2/2015 3:23 PM
443	N/A	6/2/2015 3:23 PM
444	N/A In Canada	6/2/2015 3:23 PM
445	have not come across this problem	6/2/2015 3:23 PM
446	do the tests and not get paid	6/2/2015 3:22 PM
